# Mouse Models of Inherited Retinal Degeneration with Photoreceptor Cell Loss

**DOI:** 10.3390/cells9040931

**Published:** 2020-04-10

**Authors:** Gayle B. Collin, Navdeep Gogna, Bo Chang, Nattaya Damkham, Jai Pinkney, Lillian F. Hyde, Lisa Stone, Jürgen K. Naggert, Patsy M. Nishina, Mark P. Krebs

**Affiliations:** 1 The Jackson Laboratory, Bar Harbor, Maine, ME 04609, USA; gayle.collin@jax.org (G.B.C.); navdeep.gogna@jax.org (N.G.); bo.chang@jax.org (B.C.); nattaya.damkham@jax.org (N.D.); jai.pinkney@jax.org (J.P.); Lillian.Hyde@jax.org (L.F.H.); lisa.stone@jax.org (L.S.); juergen.naggert@jax.org (J.K.N.); 2 Department of Immunology, Faculty of Medicine Siriraj Hospital, Mahidol University, Bangkok 10700, Thailand; 3 Siriraj Center of Excellence for Stem Cell Research, Faculty of Medicine Siriraj Hospital, Mahidol University, Bangkok 10700, Thailand

**Keywords:** visual photoreceptor cell loss, mouse genetic models, retinitis pigmentosa, Leber congenital amaurosis, ciliopathies

## Abstract

Inherited retinal degeneration (RD) leads to the impairment or loss of vision in millions of individuals worldwide, most frequently due to the loss of photoreceptor (PR) cells. Animal models, particularly the laboratory mouse, have been used to understand the pathogenic mechanisms that underlie PR cell loss and to explore therapies that may prevent, delay, or reverse RD. Here, we reviewed entries in the Mouse Genome Informatics and PubMed databases to compile a comprehensive list of monogenic mouse models in which PR cell loss is demonstrated. The progression of PR cell loss with postnatal age was documented in mutant alleles of genes grouped by biological function. As anticipated, a wide range in the onset and rate of cell loss was observed among the reported models. The analysis underscored relationships between RD genes and ciliary function, transcription-coupled DNA damage repair, and cellular chloride homeostasis. Comparing the mouse gene list to human RD genes identified in the RetNet database revealed that mouse models are available for 40% of the known human diseases, suggesting opportunities for future research. This work may provide insight into the molecular players and pathways through which PR degenerative disease occurs and may be useful for planning translational studies.

## 1. Introduction

Inherited forms of retinal degeneration (RD) encompass a genetically and clinically heterogeneous group of disorders estimated to cause vision impairment and loss in more than 5.5 million individuals worldwide [1,2], with 282 mapped and identified retinal degenerative disease genes documented in the RetNet human database [3]. Animal models, such as non-human primates [4], dogs [5], mice [6,7], zebrafish [8], and fruit flies [9], have been used to identify candidates for human retinal disease genes, to elucidate pathological mechanisms, and to serve as a resource for exploring therapeutic approaches. As potential therapies for retinal diseases are investigated, the need for animal models increases. Information about the disease onset and rate of progression, the pathogenic pathways involved, and the genetic background in which the disrupted genes are situated are all factors that must be considered when selecting appropriate models for testing therapeutics. These factors will also play a role in interpreting the outcome of treatment studies.

The purpose of this review is to compile a searchable list of mouse models of inherited retinal diseases caused by single gene mutations that specifically lead to the post-developmental rod and/or cone photoreceptor (PR) cell loss. To identify these models, we reviewed mouse-specific data available in the Mouse Genome Informatics (MGI) and National Center for Biotechnology Information (NCBI) databases at The Jackson Laboratory (JAX) and National Institutes of Health (NIH), respectively. We recorded, when available, PR cell loss data from publications describing mutant alleles of the genes identified. We also included representative fundus photographs and optical coherence tomography (OCT) images of selected mouse models from the Eye Mutant Resource (EMR) and the Translational Vision Research Models (TVRM) programs at JAX as examples of the retinal phenotypes found among mouse models that fit our criteria. We attempted to cluster genes based on the function and then compared the progression of PR cell loss among these clusters to provide potential insights into disease mechanisms. We also compared our list of mouse genes associated with PR cell loss with the RetNet gene list, to highlight mouse models for specific retinal diseases, to reveal opportunities to create novel models, and to identify candidate genes within human loci for which a causative gene is currently unknown. Finally, we coordinated with the MGI team to incorporate our annotations into the MGI database, which will allow future analyses using tools available through that platform. It is hoped that our work will be useful as a resource for investigators to assist in the selection of appropriate mouse models within and across functional clusters in new studies to understand and develop treatments for human retinal degenerative disease.

In the three decades since the genes linked to PR loss phenotype were first identified in the mouse and human [10,11,12], rapid progress in understanding the genetic basis of inherited RD has been summarized in many excellent reviews. Many of the topics presented in the current article have been discussed previously in reviews of mouse RD models [6,7,13,14] and in summaries of our work at JAX [15,16,17,18,19,20,21]. Although we have made every effort to acknowledge the many contributions to this field, we note that there is a large body of relevant literature and apologize in advance to authors whose reviews or articles we may have inadvertently overlooked.

## 2. Background

### 2.1. Photoreceptor (PR) Cell Structure

**Figure 1:**
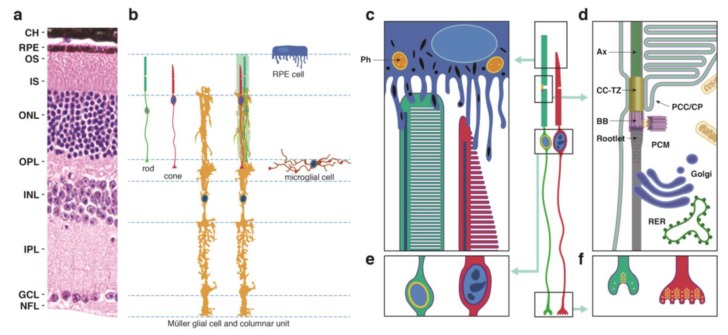
Retinal tissue organization emphasizing cell types and subcellular structures that may be sites of pathological processes in mouse models of photoreceptor (PR) cell loss. (**a**) A radial section of the posterior eye stained with hematoxylin and eosin shows the layered structure of the retina. CH, choroid; RPE, retinal pigment epithelium; IS, inner segment; OS, outer segment; ONL, outer nuclear layer; OPL, outer plexiform layer; INL, inner nuclear layer; IPL, inner plexiform layer; GCL, ganglion cell layer; NFL, nerve fiber layer. (**b**) Two PR cell types (rods and cones) and additional cell types that may be the target of processes implicated in PR cell loss. Dashed lines indicate alignment with retinal layers in (**a**). A columnar unit consisting of one cone and one Müller cell and roughly 20 rod cells is shown. (**c**–**f**) Details of PR and RPE cells. (**c**) PR cell OSs contain flattened discs (rod, left) or incomplete discs (cone, right) where the light sensing apparatus is located. OS tips engulfed by RPE cell apical processes are digested in phagolysosomes (Ph). (**d**) The base of the OS, the connecting cilium, and the apical portion of a rod cell IS (adapted with permission from [24]). The axoneme (Ax) and rootlet provide physical stability to the cilium. BB, basal bodies; CC-TZ, connecting cilium-transition zone; PCM, pericentriolar matrix; PCC/CP, periciliary complex/ciliary pocket; RER, rough endoplasmic reticulum. (**e**) The PR cell soma is largely occupied by the nucleus. (**f**) Rod and cone synaptic termini include presynaptic ribbons and associated neurotransmitter vesicles.

PR cells are sensory neurons within the retina that detect light and signal this event to other cells. Since PR cells are essential for vision, their loss can dramatically and negatively affect the quality of life. PR cells include rod and cone cells (Figure 1a,b) that occupy the outermost layers of the neurosensory retina. Although intrinsically photosensitive retinal ganglion cells have also been described as photoreceptors [22], we did not include them in this review, as their contribution to RD is unknown. Rod and cone photoreceptors possess unique structures that serve to compartmentalize processes that are critical for cell function and maintenance.

- The outer segment (OS), which is cylindrical in rod PR cells and tapered in cones, contains phototransduction proteins that sense light and amplify the ensuing signal, culminating in PR cell hyperpolarization (Figure 1c). Much of the phototransduction apparatus is localized to double-bilayer discs formed by evagination of the plasma membrane at the base of the OS. These discs are largely internalized in rods except at the base of the OS, but remain contiguous with the plasma membrane in cones to yield a highly convoluted OS surface [23].
- The OS is stabilized by a ciliary axoneme, which runs through much of its length (Figure 1d; Ax). At the proximal end of the axoneme, the connecting cilium, analogous to the transition zone in other cilia (Figure 1d; CC-TZ), serves as a conduit through which all membrane and protein components destined for the OS are thought to pass. At the base of the connecting cilium lies the basal body (Figure 1d; BB), a cylindrical organelle derived from the mother centriole. Altogether these structures represent a modified primary cilium that encompasses an extensive network of protein complexes that transport proteins and lipids and shares characteristics with primary cilia in many other cell types. The ciliary networks also function to prevent the flow of OS components to other parts of the cell and may associate with the intracellular trafficking apparatus to ensure the directed movement of needed components to the OS.

- The inner segment (IS) contains the biosynthetic machinery and energy sources needed to produce and assemble newly synthesized phototransduction proteins and their associated membranes (Figure 1d). The capacity of this cellular factory is impressive, as up to 10% of the OS is shed daily and removed via phagocytosis by the retinal pigment epithelium (see below) and must be renewed. Most protein and lipid components are synthesized de novo, but the IS also has an extensive recycling machinery that can reassemble components provided from outside the cell.
- The cell body or soma includes the nucleus, which is highly condensed in rod PR cells, but is larger in cones and includes patches of heterochromatin (Figure 1e). To increase the density of rod and cone OSs in the retina, the somas are stacked in columns within the outer nuclear layer (ONL). This arrangement necessitates thin cell extensions reaching from the soma to the IS or to the synapse. PR cell loss is measured by counting ONL nuclei, which are prominently stained in retinal sections (Figure 1a), or in the case of rods, which are more abundant than cones, by measuring ONL thickness from micrographs or by OCT.
- The PR cell terminus contains ribbon synapses close to the presynaptic membrane loaded with vesicles containing the excitatory neurotransmitter glutamate (Figure 1f). In the dark, a steady-state level of glutamate is released at the synapse, which is reduced when the cells are hyperpolarized in the light. Changes in glutamate levels at the synapse signal postsynaptic secondary neurons in the inner nuclear layer, which communicate with ganglion cells on the vitreal surface of the retina that connect through long axons to the visual cortex of the brain.

### 2.2. Neighboring Cells

Müller glia are radial cells that span much of the neurosensory retina, reaching from the internal limiting membrane at the vitreal surface of the retina to the external limiting membrane on the scleral edge of the ONL [25]. Within the ONL, fine Müller cell extensions appear to ensheath the PR cell soma. As they also interact with vascular layers within the retina, Müller cells may provide essential nutrients to PR cells, which do not directly contact the circulation. They also regulate extracellular volume, ion and water homeostasis, serve to modify neuronal activity through release of neuroactive compounds, and modulate immune and inflammatory responses [26]. At the external limiting membrane, Müller cell endfeet engage rod and cone cell ISs in intercellular adhesion interactions, including tight junctions, which create a diffusion barrier. Notably, the arrangement of an Müller cell, rods, and a cone cell has been proposed to form a columnar unit (Figure 1b), which may result in physiological and functional coordination of these cell types.

RPE cells (Figure 1b,c) constitute an epithelial monolayer that lies between the retina and a capillary bed, the choriocapillaris. The flow of water, ions, small molecules, and metabolites from the blood to the outer retina is thus regulated by RPE cells. Their apical surface features microvilli and microplicae (Figure 1b,c) that contact roughly the outermost third of OSs and play important roles in recycling molecules needed for PR renewal. These apical processes also mediate initial steps in the daily phagocytosis of OS tips. As an epithelium with high-resistance intercellular junctions [27], the RPE performs an important barrier function, disruption of which may cause PR degeneration.

Microglial cells form ramified networks within the same retinal layers as the retinal vasculature, which includes the superficial, intermediate and deep vascular beds. Microglia at the level of the outer plexiform layer in healthy retinas extend dendritic arms into the ONL (Figure 1b), where they contact PR soma as part of a dynamic survey process. During development and in rare events that occur in healthy retinas, these cells engulf and phagocytose PR soma, presumably in response to a defect in PR function.

PRs form synaptic connections in the outer plexiform layer with secondary neurons, including bipolar and horizontal cells. Although these connections are critical for signal transmission, they are not as extensive as the contacts made between PRs and Müller glia, RPE cell apical processes, or the dynamic extensions on microglial cells, and were therefore omitted from the summary diagram in Figure 1. Nevertheless, perturbation of the interactions among these cells may lead to PR degeneration, conceivably due to an alteration of signal transmission.

### 2.3. Inherited Diseases that Cause PR Cell Loss

Major monogenic inherited RDs in which PR cells are lost include: retinitis pigmentosa [28], Leber congenital amaurosis [29], and syndromic disorders that manifest disease in multiple organs, including the eye, particularly ciliopathies, such as Joubert [30], Bardet–Biedl [31], or Usher [32] syndrome. The remarkable success of gene augmentation therapy for a form of Leber congenital amaurosis has invigorated research efforts to treat these diseases [33].

## 3. Methods

### 3.1. Public Database and Literature Searches

**Figure 2:**
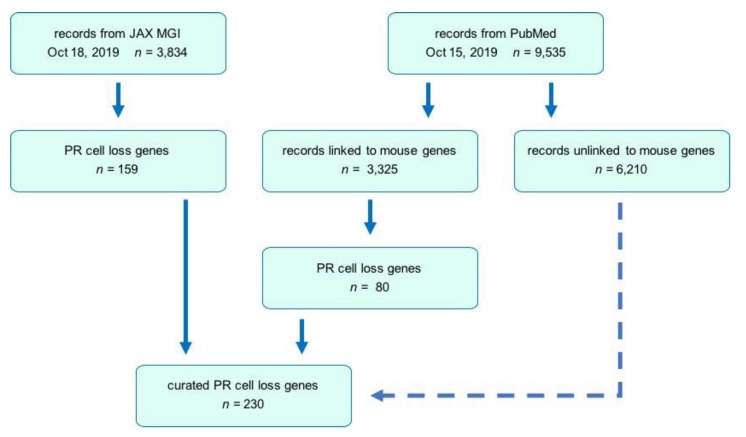
Flow chart depicting the progression of the search strategy utilized in this review. The number of records or genes identified from them is indicated at each stage of the process. The dashed line indicates that some genes were identified from records that remain to be systematically screened.

The search strategy employed in this review is summarized in Figure 2. Initially, the MGI database [34] was queried to identify mutant protein-coding genes associated with a mammalian disease phenotype indicating a loss of PR cells. MGI is a curated database that includes expert annotation based on full-text searching of 148 selected journals, which is limited compared to literature databases, such as PubMed. Typically, only the first paper describing a new allele is fully curated for phenotype data in the database due to resource constraints. Although our analysis yielded 159 mutant genes that were associated with PR cell loss, a number of mutant genes known to cause this phenotype were absent or not annotated, possibly due to these aforementioned limitations.

To expand the search, we used NCBI databases, including PubMed [35] and Gene. We refined a PubMed query by searching with keyword phrases to generate article lists, using the Gene option of Find Related Data to yield mouse genes linked to the articles, and then assessing whether these genes were on our MGI list. The goal was to develop a broad query that included as many genes from the MGI list as possible but also included additional hits. The most successful was: (ONL OR “outer nuclear layer” OR retina* OR PR OR rod OR rods OR cone*) AND (degener* OR loss OR thin* OR thick*) AND (mouse OR mice OR murine), which captured >97% of the MGI list. Restricting this query to entries posted to PubMed on or before October 15, 2019, yielded 9535 articles. To review these articles efficiently we tried two approaches, the first generating a spreadsheet containing hyperlinks in which each linked gene symbol was combined with the Boolean query, and the second using mouse gene identification numbers corresponding to the linked genes and applying an Entrez script that accessed the Gene and PubMed databases to find all articles satisfying the Boolean query for each linked gene.

### 3.2. Search Strategy

Each MGI database-derived entry was curated manually or automatically to identify candidate models that reported PR degeneration as a phenotype, as described above. In the case of PubMed entries, although the automated approaches were useful for quickly identifying genes that satisfied our criteria, neither was comprehensive, and additional candidate models were identified by review of the title and abstract from some of the remaining articles in the full collection of 9535 articles. Subsequently, an independent coauthor identified an original publication for each candidate gene and determined if PR cell loss was reported. If sufficient evidence for PR cell loss was obtained, the gene and mouse model was assigned to one of 11 categories. Genes within each category were curated further by coauthors who identified alternate alleles and extracted information regarding the disease phenotype induced by the disruption of a gene. Each entry in Table S1 is the result of the examination of an original article (indicated by PubMed ID numbers, PMIDs) and data from MGI to capture information such as mutation type, associated human diseases, and disease onset and progression.

### 3.3. Comparative Analysis and Updating the MGI Database

Once our final list was completed, we used tools in Excel to compare it to a list constructed from online tables downloaded on 8 December 2019, from RetNet, a public compilation of human genes linked to inherited RD. We also provided our data to the MGI team at JAX, who assigned allele nomenclature, added strain information for newly described mutants, and updated phenotype data for alleles that were present in the MGI database but not yet annotated with respect to PR cell loss. This review has been referenced at MGI so that the alleles documented in the article can be examined using MGI tools or downloaded in tabular format for analysis with other software. The collaborative approach between mouse phenotyping experts and the MGI team may be attractive for ensuring that this useful resource remains current in the face of limited funding, personnel, and time.

### 3.4. Inclusion/Exclusion Criteria

Monogenic models generated from a variety of sources were included in Table S1. However, in the case of conditional models, only those for which a germline null allele was reported in the MGI database that resulted in embryonic, prenatal, or postnatal lethality were included. We excluded the following models from Table S1: those for which a causative gene had yet to be identified and for which complementation tests were unavailable; those requiring multiple genes for the presentation of the disease phenotype; those based on overexpressing transgenes; and those in which PR degeneration depended on experimental interventions, such as an altered diet, drug treatment, or exposure to bright illumination. Environmental influences on retinal diseases are very important and may affect the progression of PR cell loss, but models that depend on environmental conditions are challenging to compare because of the significant variation among the types of environmental perturbations and the methods used to apply them. We also excluded models that exhibited a reduction in the PR cell number during development but not a progressive loss with age, and those where IS and/or OS dysmorphology or reduction in length was observed without a loss of PR cells, as indicated by a reduction in nuclei number or ONL thickness within the time frame reported in the papers. Although these models were excluded from Table S1, examples are included in the Results.

### 3.5. Heterogeneity of Data

The type and frequency of data gathered varied greatly among the studies reviewed. In some papers, only one figure with one retinal section was offered as evidence for PR degeneration, while other papers showed extensive quantitation of their data. To document potential sources of variability in the data, we indicate the method by which the degree of degeneration was determined, either by measuring ONL thickness or by count of nuclei in the ONL, typically the number of rows of nuclei spanning the ONL but sometimes a total count of ONL nuclei in a fixed area of a retinal micrograph. In some instances, when data was quantified in spider plots or bar graphs, mean values obtained from the central retina were used in estimating the PR loss. We normalized the data among studies by recording the percent degeneration as determined by dividing the mutant values by the corresponding values from age-matched controls as reported in each publication.

### 3.6. Comparison of Progressive PR Cell Loss

To compare progressive PR cell loss among models, we fit normalized data from each article to an exponential decay that includes a delay, or offset [36]. Ranges of either age or photoreceptor numbers, if reported, were averaged. Fitting was performed in Excel Visual Basic using a piecewise equation that modeled the delay with a straight line at 100% and the remaining points with a monoexponential decay to 0%. Two adjustable parameters, the delay and the decay rate constant, were optimized. We calculated the age at which PR cell numbers reached 50% of control values (D_50_) as a measure of progression. Roughly one third of the datasets contained only a single point within the exponential regime, which was insufficient to calculate D_50_. In these cases, D_50_ was calculated at the extremes of zero delay and infinite rate, and the mean of these values was used as a D_50_ estimate.

### 3.7. Generation of Primary Data Using Fundus Imaging and OCT Scans

Fundus photographs of EMR mutants were taken in unanesthetized mice treated with 1% cyclopentolate to dilate or enlarge the pupil with an in vivo bright field retinal imaging microscope equipped with image-guided OCT capabilities (Micron III; Phoenix Laboratories, Inc., Pleasanton, CA, USA) as previously described [20]. This system allows for the visualization of the location of the OCT scan using the real-time Micron III bright-field image. A superimposed line placed directly on the image over the retinal feature being examined delivers precise cross-sectional information, allowing for the assessment of changes in layer thickness and morphological alterations.

Fundus photodocumentation for TVRM mutants and C57BL/6J control mice was performed using a Micron III or IV retinal camera (Phoenix Laboratories, Inc., Pleasanton, CA, USA) as described [37], except that 1% cyclopentolate or 1% atropine was used as a dilating agent, and in some cases, mice were anesthetized with isoflurane. OCT imaging to assess retinal layer thickness in *Nmnat1^tvrm113^*, *Ctnna1^Tvrm5^*, and C57BL/6J control mice was performed using a Bioptigen ultrahigh-resolution (UHR) Envisu R2210 spectral domain OCT (SDOCT) imaging system for volume scanning as described [37,38] with ketamine/xylazine (1.6 mL ketamine (100 mg/mL), 1.6 mL xylazine (20 mg/mL), and 6.8 mL sodium chloride (0.9% *w/v*)) as an anesthetic. A representative B-scan through the optic nerve head was derived from the OCT volume dataset. *Rpgrip1^nmf247^* and *Alms1^Gt(XH152)Byg^* were assessed on the same OCT system by obtaining a linear B-scan with the following parameters: length, 1.9 mm; width, 1.9 mm; angle, 0 degrees; horizontal offset, 0 mm; vertical offset, 0 mm; A-scans/B-scan, 1000 lines; B-scans, 1 line; frames/B-scan, 20 frames; and inactive A-scans/B-scan, 80 lines. Linear scans were registered and averaged in the InVivoVue program to merge the 20 frames into a single image.

## 4. Results

### 4.1. Summary of Studies that Report PR Cell Loss

**Figure 3:**
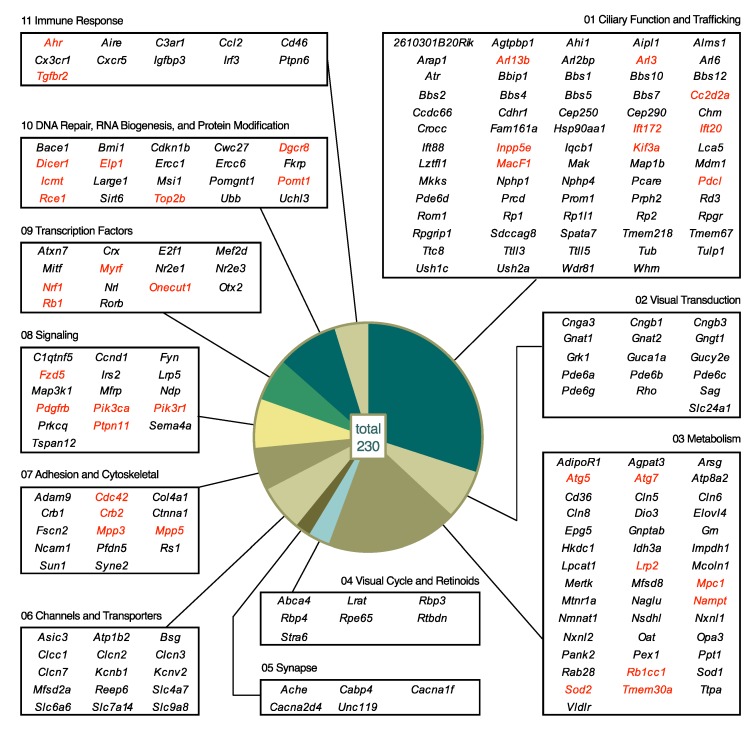
Genes associated with PR cell loss in monogenic mouse models of retinal degeneration (RD). Genes identified by combined review of the Mouse Genome Informatics (MGI) database and articles from a PubMed query were assigned to the indicated functional categories as described in the text. Genes for which mutant alleles are available only in the conditional form are displayed in red. Conditional alleles were included only in instances where germline null alleles resulted in embryonic, prenatal or postnatal lethality. For additional details on inclusion/exclusion criteria, see Section 3.4.

The combined searches of MGI and PubMed databases yielded a total of 230 genes associated with PR cell loss. Ultimately, 3834 reports at MGI and 3325 at PubMed, which most typically characterized one mutant gene but on rare occasions described more than one, were used in the present review. The distribution of retrieved publications sorted by functional categories is summarized in Table S1. The genes identified in these models are summarized in Figure 3. Descriptions of gene and protein symbols used in the text, figures, and Table S1 are provided in Table S2.

#### 4.1.1. PR Cell Loss Models

The mouse models described in Table S1 were either spontaneous (12%) or chemically induced mutants (11%), or those produced through genetic engineering approaches (77%). This latter group, which was by far the largest, utilized standard homologous recombination, gene-traps, nuclease mediated approaches such as CRISPR/Cas9, and conditionals to mediate genomic changes. Additionally, four models of inadvertent transgene insertion into a unique gene, whose disruption led to PR degeneration, were included within this group. Interesting examples of differences in the disease onset or rate of progression were demonstrated in different models of the same gene (e.g., *Aipl1*) that may be related to allelic differences, null versus missense mutations, or genetic background effects [21,39,40]. Most of the genetically engineered models in Table S1 tended to be in mixed, segregating genetic backgrounds that might impact phenotypic expression (discussed below).

Within the genetically engineered category, a relatively large group of models, 16%, were conditional models, representing 39 genes (Figure 3; red). Generation of conditional mutants is based on the Cre-Lox recombination approach, which requires a floxed gene and a cre-driver to excise the targeted genomic region in a spatial (e.g., cell/tissue specific) or temporal manner (e.g., induction by chemicals such as doxycycline). Since 30% of all null mutations lead to embryonic lethality, as they represent genes that are essential during development, conditionals are often used to examine the adult function of genes [41]. This was the case in 92% of the conditional models described here, as standard organism-wide removal of the genes was reported to be embryonic, perinatal, or postnatal lethal. Thus, conditionals allow us to learn the function of a gene post-developmentally. Conditionals are also sometimes used to determine the cellular contributions to a disease phenotype. If a gene is expressed in multiple retinal cell types, by removing them systematically and examining the consequent phenotype, one can learn how the loss of function of the gene within particular cell types affects the disease phenotype. For example, removal of *Arl3* from rod PRs using a Rho-icre driver shows a later and slower rate of degeneration than that found with Six3-cre, a Cre driver that expresses in early retinal development. This suggests that *Arl3* in rods is necessary for PR survival but that *Arl3* function in other retinal cell types also affects PR survival [42]. The most widely used Cre models include: for targeting retinal progenitor cells, Tg(rx3-icre)1Mjam, Tg(Six3-cre)69Frty, Tg(Chx10-EGFP/cre,-ALPP)2Clc, Tg(Crx-cre)1Tfur, and Tg(Pax6-cre,GFP)2Pgr; for targeting rods, Tg(Rho-icre)1Ck, Tg(RHO-cre)8Eap, and *Pde6g^tm1(cre/ERT2)Eye^*; for targeting M-cone PRs, Tg(OPN1LW-cre)4Yzl (also known as HRGP-cre); for targeting PRs, Tg(Rbp3-cre)528Jxm (also known as IRBP-cre); for targeting RPE, Tg(BEST1-cre)1Jdun, Tg(BEST1-rtTA,tetO-cre)1Yzl and *Foxg1^tm1(cre)Skm^*; and for targeting adult tissues using tamoxifen, Tg(CAG-cre/Esr1*)5Amc.

#### 4.1.2. Mouse Models from Phenotyping Programs

The models listed in Table S1 come from many sources. In addition to individual investigator-initiated efforts, currently the largest contributor to ocular models is the International Mouse Phenotyping consortium, in which 19 phenotyping centers from 11 countries participate to systematically characterize knockout mice generated in a standardized manner [41,43]. All centers do some eye phenotyping, thus providing a window into potential models. Although only a few models from this program are included in Table S1, as most are not yet fully characterized, it is anticipated that this consortium will provide a wealth of models for individual laboratories to study. For example, in the MGI database, 39 IMPC models were identified with “reduced retinal thickness” that with further characterization may reveal PR degeneration.

**Figure 4:**
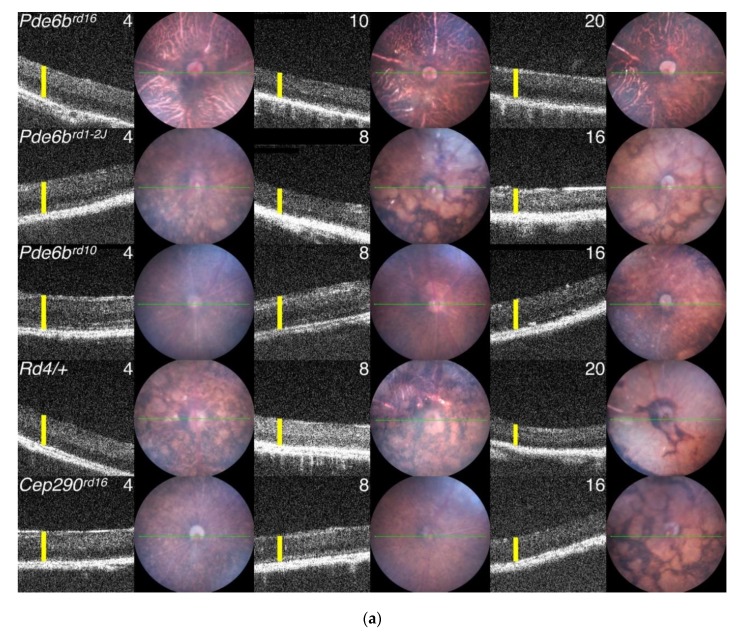

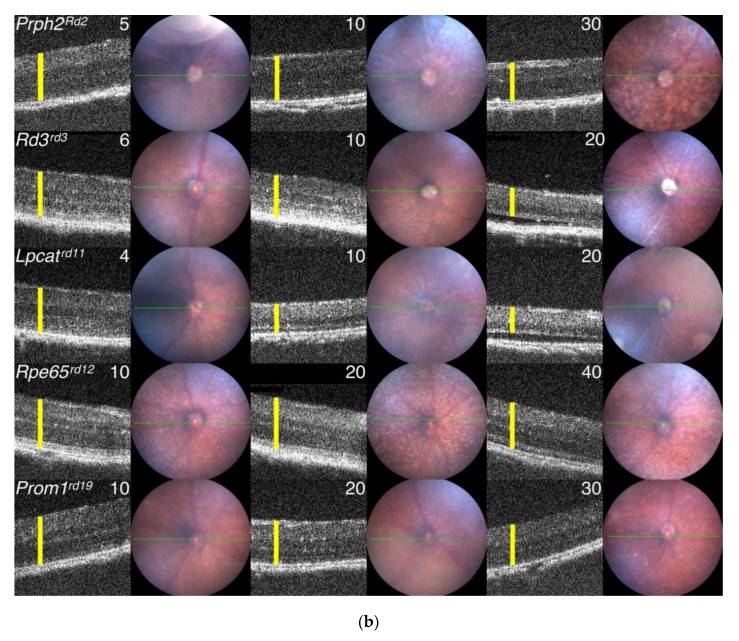

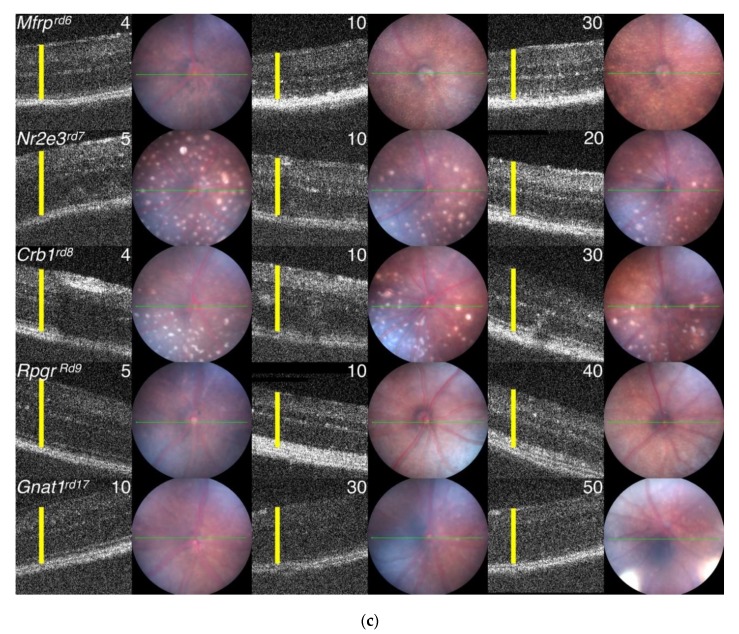
(**a**) Characterization of mouse models from the Eye Mutant Resource (EMR) program at JAX. Example optical coherence tomography (OCT) and fundus images were taken from rapid RD models: *Pde6b^rd1^* (B6.C3-*Pde6b^rd1^ Hps4^le^*/J, Stock No: 000002), *Pde6b^rd1-2J^* (C57BL/6J-*Pde6b^rd1-2J^*/J, Stock No: 004766), *Pde6b^rd10^* (B6.CXB1-*Pde6b^rd10^*/J, Stock No: 004297), *Rd4/+* (STOCK In(4)56Rk/J, Stock No: 001379) and *Cep290^rd16^* (B6.Cg-*Cep290^rd16^*/Boc, Stock No: 012283) (**b**) Example images from slower RD models: *Prph2^Rd2^* (C3A.Cg-*Pde6b*^+^ *Prph2^Rd2^*/J, Stock No: 001979), *Rd3^rd3^* (B6.Cg-*Rd3^rd3^*/Boc, Stock No: 008627), *Lpcat1^rd11^* (B6.Cg-*Lpcat1^rd11^*/Boc, Stock No: 006947), *Rpe65^rd12^* (B6(A)-*Rpe65^rd12^*/J, Stock No: 005379) and *Prom1^rd19^* (B6.BXD83-*Prom1^rd19^*/Boc, Stock No: 026803). (**c**) Examples from slow and very slow RD models: *Mfrp^rd6^* (B6.C3Ga-*Mfrp^rd6^*/J, Stock No: 003684), *Nr2e3^rd7^* (B6.Cg-*Nr2e3^rd7^*/J, Stock No: 004643), *Crb1^rd8^* (STOCK *Crb1^rd8^*/J, Stock No: 003392), *Rpgr^Rd9^* (C57BL/6J-*Rpgr^Rd9^*/Boc, Stock No: 003391), and *Gnat1^rd17^* (B6.Cg-*Gnat1^irdr^*/Boc, Stock No: 008811). *Yellow bars* indicate full retinal thickness. Values correspond to the mouse age at the time of imaging (weeks).

**Figure 5:**
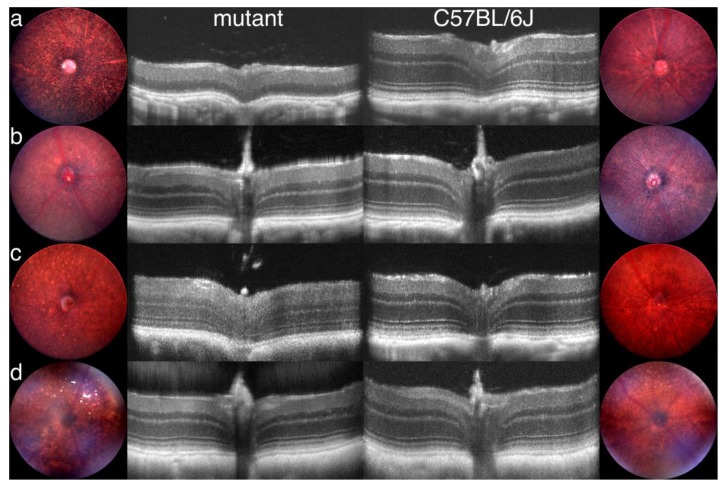
Characterization of mouse models from the Translational Vision Research Models (TVRM) program at JAX. A fundus image (circular panels) and corresponding OCT B-scan are shown for homozygous (**a**) *Rpgrip1^nmf247^*, at one month of age; (**b**) *Nmnat1^tvrm113^*, at two months; (**c**) *Alms1^Gt(XH152)Byg^*, at one year; and (**d**) *Ctnna1^Tvrm5^*, at two years. Age-matched OCT and fundus images for C57BL6/J control mice are shown to the right of the mutant images. PR cell loss is indicated by a decreased ONL thickness. Fundus images were acquired using a Micron III or IV retinal camera. The vertical dimension of OCT images was doubled to emphasize changes in retinal layer thicknesses.

At The Jackson Laboratory, the Eye Mutant Resource (EMR) and the Translational Vision Research Models (TVRM) programs are dedicated to screen for or generate mouse models with ocular diseases. The EMR has been screening retired breeders by slit lamp biomicroscopy, indirect ophthalmoscopy, and electroretinography since 1988. Retired breeders from the production and genetic resources colonies are screened. Heritable mutants are phenotypically and genetically characterized and the spontaneous mutants are distributed worldwide. The TVRM program arose from the JAX Neuromutagenesis Facility. Mice for this program are generated by chemical mutagenesis or genetic engineering. Carefully characterized mutants are also distributed. Examples of mutants from the EMR and TVRM programs are shown in Figure 4 and Figure 5, respectively.

## 5. Analysis

### 5.1. Progression of PR Cell Loss

While a host of effects can occur as a result of disruptions in genes expressed in PRs and ancillary cell types that functionally impair vision, such as night blindness, or color vision defects, the focus of the models described here are those that bear single gene mutations that lead to actual PR cell loss. From a review of the models in Table S1, significant PR cell loss is reported as early as postnatal day 7 (P7) and can extend throughout the lifetime of animals examined. The progression of PR cells loss is also highly variable and includes models that progress rapidly with complete ablation within several weeks to models with extremely slow progression where only <10% PR cell loss is noted over the span of time in which animals were examined. Generally, while rapid to moderate progression led to almost complete PR ablation, slow and very slow progression, or degeneration in models that primarily affect cone PRs left a substantial number of PR cells intact.

**Figure 6:**
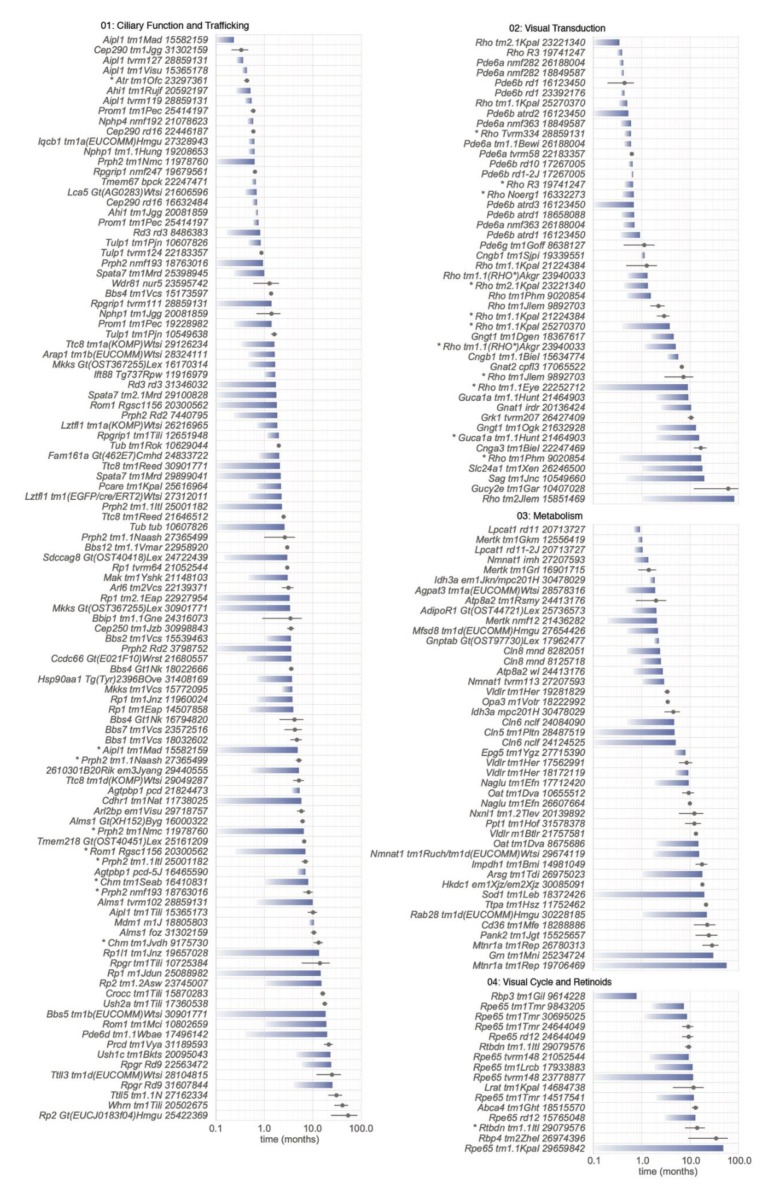

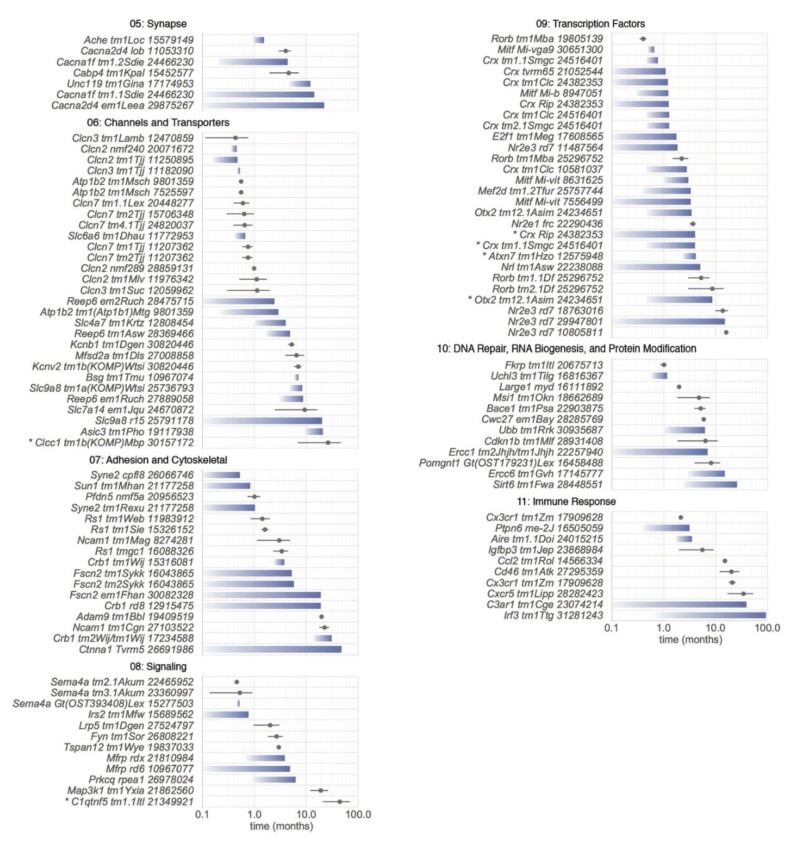
Progression of PR cell loss in mouse models by functional category. Models in each category were sorted by the age at which the estimated number of PR cells reached 50% of wild-type (D_50_) as determined by fitting reported data to an exponential decay function combined with a delay. Models are identified by gene symbol, MGI allele symbol, and PMID. Models are homozygous except as indicated by * (heterozygous) or by the presence of two allele symbols (compound heterozygous). Left- and right-hand margins of shaded bars represent the delay and D_50_ values, respectively. Bars with a delay value of ≤0.1 month derive from datasets in which no values at 100% of wild type were reported. The midpoint and range of estimated D_50_ values for datasets with only one point in the 5–95% range is indicated by closed circles and range lines, respectively. In cases where the dataset consisted of a single point at 50%, no estimate was needed.

To compare cell loss among functionally similar genetic models, models in each category of Table S1 were sorted based on the estimated age at which the PR cell population had degenerated by 50% compared to control values, defined as D_50_ (Figure 6). This quantity represents neither the rate of PR cell loss nor the delay before loss commences, although both parameters are used to calculate it. Rather, D_50_ provides a common measure of progression that allows both complete and sparse datasets to be evaluated. With sufficient data, delay, and exponential decay constants were calculated and are reflected by the shaded bars in Figure 6. Many datasets with fewer measurements, some containing a single point, allowed only an estimate of D_50_ (Figure 6; filled circles, range lines indicate estimated limits). Figure 6 may be used to identify models in which overall PR cell loss progresses at an earlier age (lower D_50_), proceeds at a higher rate once initiated (shorter bar), or is accompanied by a substantial delay (bar starts farther to the right), which may aid in experimental design. Values for D_50_, the exponential decay constant k, and the delay, when available, are also included in Table S1. We relate qualitative descriptions of progression to D_50_ as follows: rapid, <2 months; moderate, 2 to <6 months; slow, 6 to <12 months; and very slow, ≥12 months.

Figure 6 does not include models in which the age of the animal at the time of measurements was not provided, or was reported as “adult”, although these models were included in Table S1. We also omitted models where only cone PR cell degeneration was reported, as limited data and a lack of cone nuclei counts made mathematical modeling of cell loss unreliable. Finally, we omitted all conditional alleles from Figure 6, as the efficiency and specificity of inactivation of genes, as well as the temporal expression of different transgenic Cre drivers utilized varies, making comparison of these alleles difficult.

### 5.2. Biological Processes Affected by Mutations

Categorization of PR cell loss genes (Figure 3) relied on current functional knowledge, similarity to other genes, and localization, if known. Genes are often expressed in multiple cell types and serve different functions within the retina. We placed genes in categories that were most pertinent to their roles in the PR or effect on PR survival. The data in Table S1 can be explored after reassigning models to different categories if desired. In the descriptions below, we provide an overview for the first three categories and then a detailed description of the effect of gene disruptions on biological functions included in these categories. Subsequent categories are described without overview.

#### 5.2.1. Category 01: Ciliary Function and Trafficking

Overview. Disruptions in a vast number of genes associated with PR sensory cilia result in RD [24,44]. The onset and rate of PR cell loss may vary depending on the role of a given gene in maintaining ciliary structural integrity and function during early PR development and maintenance in adulthood. Retinal defects within any one of the key ciliary structures and/or processes such as ciliogenesis, OS morphogenesis, or PR homeostasis may result in the loss of rod and cone PR cells. Understanding the pathophysiological mechanisms involved in PR cell loss requires a detailed examination of the components and functionalities of the PR sensory cilium.

The mouse PR sensory cilium is a specialized structure comprised of a tubulin-rich axoneme with a symmetrical “9+0” arrangement of microtubular doublets [45]. The connecting cilium (CC) is akin to the transition zone (TZ) of primary cilia [46,47] and serves as a passageway for the movement of proteins from the organelle-rich IS to the photosensory OS. The OS is composed of an elongated distal axoneme with adjacent stacked membranous discs decorated with proteins necessary for phototransduction. It has been hypothesized that extension of the PR plasma membrane towards the apical RPE provides a convenient sink in the OS for the storage of a large number of membrane proteins [48]. In the CC-TZ, axonemal microtubule doublets interconnect the distal axoneme and basal body, and also connect to the periciliary membrane via Y-linkers. The CC-TZ harbors membrane-associated and soluble proteins that coordinate as gatekeepers to regulate the entry, retention, and exit of proteins in and out of the OS [47,49,50]. At the axonemal base, the ciliary membrane is anchored by the nine microtubular triplets of the basal body and its associated appendages.

In developing murine PRs, the connecting cilium and OSs assemble through a series of coordinated events. Shortly after cell cycle exit of retinal progenitor cells, the mother centriole docks to a primary ciliary vesicle initiating its expansion and fusion with the plasma membrane. Concurrently, the microtubular axoneme elongates towards the apical end of the neuroblastic layer [51]. In rod PRs, ciliogenesis typically begins shortly after birth with the appearance of a mature centriolar-bound ciliary vesicle at around P4 [52]. Subsequently, OS biogenesis occurs asynchronously between P8 and P14 [52,53,54] while axoneme and PM extension continue until OS maturation around P19–25 [52,53]. During this process, intraflagellar transport (IFT) provides an efficient mechanism for the movement and delivery of crucial proteins to the developing OS [55,56], as discussed in greater detail below.

Over the past several decades, two disparate mechanisms of rod disc morphogenesis have been debated, a vesicular fusion model [57,58], which postulates that membrane discs originate from rhodopsin bearing vesicles that undergo intracellular membrane fusion, and the classic evagination model [59], which proposes that new discs result from the evagination of the plasma membrane at the base of the OS. Recent ultrastructural studies in mouse rod PRs have provided compelling evidence that support the classic evagination model. By high resolution microscopy, several groups demonstrated the plasma membrane origin of the evaginated rod disc membranes [60], which subsequently flatten, elongate, and become enclosed [52,60,61].

OS membranous discs undergo a rapid and continuous turnover with approximately 75 rod discs being shed daily, corresponding to 10% of the OS [53,62]. At the ciliary tip, aged discs are removed by the adjacent RPE through phagocytosis. Consequently, OS renewal requires a continuous flow of new proteins from IS to the OS through the connecting cilium, a process that requires careful regulation. For the CC-TZ gate to function properly, efficient mechanisms are needed to prevent the entry of undesired proteins and to remove the non-OS proteins improperly targeted to the OS. At the base of the CC-TZ lie transition fibers, which act as a barrier in conjunction with other CC-TZ components, such as the membrane-associated Meckel syndrome (MKS) complex. The BBSome, an octameric coat complex, is thought to coordinate the delivery and removal of proteins from the OS during ciliary formation and maturation [63,64].

Finally, protein trafficking through the CC-TZ is important for PR development and maintenance [51,65,66]. The movement of protein cargo from the IS to the OS requires a highly regulated passage of vesicles along microtubules that dock and fuse with the periciliary membrane to deliver their cargo at the CC base. Movement of targeted proteins through the cilia to the OS may be facilitated by IFT transport machinery [65] or by a lipidated protein trafficking system [67,68]. During IFT, protein cargo associate with IFT particles that attach to kinesin and dynein motors and move along the axonemal microtubules in both anterograde and retrograde directions, respectively. The BBSome is known to associate with IFT particles and may provide a mechanism for the removal of non-targeted protein accumulation in the OS [69].

Ciliogenesis. As the ciliary axoneme serves as an important conduit for the IFT movement of signaling molecules, it comes as no surprise that the disruption of ciliogenesis genes may result in significant developmental abnormalities causing early lethality and/or rapid PR degeneration. Genes essential for the elongation of the proximal axoneme (A and B tubules) include *Kif3a* [70], which encodes a subunit of kinesin 2, *Iqcb1* [71], *Arl3* [42], and *Arl13b* [72]. While patients with missense mutations in *ARL13B* present with Joubert-associated features [73], a null mutation, *Arl13b^hnn^*, results in embryonic lethality in mice [74]. Axonemal disturbances and a failure to form OS discs are observed in developing retinas with conditional *Arl13b* disruption [72].

Axonemal and ciliary membrane extension. Disruptions in genes that affect ciliary extension include *Rp1/Rp1l1/Spata7* (distal axoneme)*, Mak,* and *Pcare (C2orf71;* ciliary membrane). Mice with knockout alleles of *Rp1* (*Rp1^tm1Eap^* and *Rp1^tm1Jnz^*) and *Spata7* (*Spata7^tm1Mrd^*) show a progressive, moderate loss in PR cells through the first year of life. *Rp1^m1Jdun^* mice homozygous for a Leu66Pro missense mutation experience a much slower degeneration with 30% of PRs left at 26 months of age. Conditional ablation studies of *Spata7* in PRs and in the RPE have shown that the disruption of SPATA7 in rod and cone PRs, but not in the RPE, is the molecular basis of the retinal degenerative phenotype [75].

Ciliary Gate and the CC-TZ. Sensory/primary cilia and their gatekeepers (CC-TZ) are found abundantly in most cell types [76]. Thus, the disease spectrum of ciliary proteins is extensive given their roles in ciliary trafficking, signaling, and development. Disruptions in CC-TZ genes may result in isolated cases of inherited retinal dystrophies such as Leber congenital amaurosis or in multisystemic, ciliopathies such as Joubert, Meckel, or Senior-Løken Syndrome. Such syndromic ciliopathies may include a multitude of disease phenotypes such as brain malformations, renal cysts, nephronophthisis, and retinal dystrophy.

Within the CC-TZ reside MKS and NPHP modules that closely interact and form multiple distinct protein complexes [47,50,77,78,79]. The MKS complex includes membrane-associated proteins, such as MKS1 and TMEM67, while NPHP complex proteins, such as NPHP1 and NPHP4, associate in closer proximity to the ciliary axoneme. Mice harboring mutations in genes coding for these complex-associated proteins form normal cilia, however, display early abnormalities in OS morphogenesis. After ciliary biogenesis, retinas in these mutant mice quickly degenerate, eliminating most PRs by 3–4 weeks of age. Genes whose disruptions affect the ciliary gate functions of the CC-TZ and cause rapid degeneration include *Nphp1*, *Nphp4*, *Ahi1*, *Iqcb1*, *Tmem67*, and *Cep290*. In humans, mutations in *CEP290* can lead to primarily single-organ diseases such, as retinitis pigmentosa and nephronophthisis, or pleiotropic diseases, such as the Joubert, Meckel, and Bardet–Biedl syndromes. The most studied allele is *rd16,* which harbors a 297 basepair in-frame deletion in *Cep290*. Compared to *Cep290^tm1.1Jgg^* knockout mice, which show a rapid 78% loss at P14, *Cep290^rd16^* homozygotes have a longer disease progression with a 60% ONL loss at three weeks of age.

Basal bodies and associated pericentriolar material (PCM). The basal body is a structure derived from the mother centriole and resides at the base of the cilium along with the daughter centriole, neighboring centriolar satellites and other related PCM. Proteins positioned at the ciliary base can also be seen in centrosomes of dividing cells (ALMS1, CEP250, and C8ORF37). Specifically, ALMS1 and CEP250 (CNAP1) localize in close proximity to each other at the proximal ends of centrioles [80]. *ALMS1* encodes a 460kDa protein that when disrupted results in the Alström syndrome (ALMS) [81,82]. Mice with a gene trap, frameshift, and nonsense mutations recapitulate human ALMS disease features such as obesity, diabetes, and neurosensory deficits [21,83,84]. The proper formation of the connecting cilium and the slow progression of PR cell loss in *Alms1^Gt(XH152)Byg^* [83], *Alms1^foz^* [84], and *Alms1^tvrm102^* [21] models suggests that ALMS1 is not essential for ciliary biogenesis but necessary for overall PR homeostasis.

The ciliary base contains supportive structures necessary for the proper docking of cargo to the ciliary membrane. Targeted *Macf1* null mutants fail to develop the ciliary vesicle needed for basal body docking while conditional ablation of *Macf1* in the developing retina disrupts retinal lamination and maturation [85]. Mutations in *Cdcc66*, which encodes a component of centriolar satellites and *Sdccag8*, which encodes a recruiter of PCM, result in an early onset but slow-moderately progressive disease [86,87]. The slower RD makes these alleles attractive models for therapeutic investigations.

Genetic mutations in *CC2D2A*, which encodes a component of the subdistal appendages of mother centrioles and basal bodies [88], have been observed in patients with Meckel Syndrome [89], Joubert Syndrome [90], and non-syndromic rod-cone dystrophy [91]. Mice with null mutations in *Cc2d2a* experience embryonic lethality due to the absence of subdistal appendages and nodal cilia [88]. The retinas of adult mice with tamoxifen-induced deletion of *Cc2d2a* in PRs have a significantly diminished ONL (2–3 layers) 12 weeks post-injection [92], suggesting that CC2D2A is necessary for ciliary homeostasis.

Periciliary membrane complex. At the periciliary membrane complex of PRs lies an Usher protein interactome complex that provides a scaffold for the anchoring of fibers to the periciliary membrane [93]. Mutations in genes encoding members of this complex, *Ush1c*, *Whrn*, and *Ush2a*, result in Usher syndrome, a disease that results in progressive hearing and vision loss. Multiple forms of Usher syndrome exist resulting in different degrees of the onset and severity of disease symptoms. In the mouse, targeted mutations in Usher genes results in a late-onset and very slow progression of PR degeneration. Homozygous *Ush2a^tm1Tili^* mice have normal retinas at 10 months of age and lose 70% of their PRs by 20 months of age [94]. PR degeneration in *Whrn^tm1Tili^* retinas is protracted with only 30% loss observed at 28 months of age [95].

Disc morphogenesis. Although the molecular mechanisms involved in OS disc morphogenesis are not completely understood, there has been considerable progress within the past decade with the emergence of refined ultrastructural methods. The OS protein, peripherin-2 (PRPH2), localizes to the rims of rod and cone discs and functions to establish and maintain the membrane rim curvature during disc formation and maintenance [96,97]. Recent investigations using the *Prph2^Rd2^* (*rds*) mouse model [10] have suggested another role for PRPH2 during disc morphogenesis [52]. Using transmission electron microscopy, Salinas et al. [52] demonstrated that like other forms of cilia [98,99], PR sensory cilia have an innate ability to spew off ectosomes at the OS base. During normal development of the OS, ectosome release is inhibited and the retained membrane at the CC-TZ is transformed into discs upon membrane evagination. In the homozygous *Prph2^Rd2^* mice, discs fail to form resulting in the accumulation of ectosomes at the OS base. This finding led the authors to propose that PRPH2 may play a role in inhibiting ectosome release during normal rim formation [52].

Knock-in mice carrying heterozygous alleles of *Prph2* (Tyr141Cys [100] and Lys153∆ [101], mimic the dominant RP disease observed in human patients. It is interesting that PR degeneration rates vary among *Prph2* mutant alleles. While homozygous *Prph2^Rd2^* mice gradually lose their PRs within the first year [102,103], mice harboring a homozygous null mutation, *Prph2^tm1Nmc^* undergo a faster degeneration with most PRs lost by 4 months of age [104]. Heterozygous *Prph2^tm1Nmc^* mice also experience PR loss, however the rate of decline is much slower [104]. Comparative studies of *Prph2^Rd2^* with rhodopsin double-knockout mice have suggested that abnormal accumulation of mislocalized rhodopsin may contribute to PR degeneration in *Prph2^Rd2^* [105]. Hence, the zygosity differences in degeneration rates may be a result of varying rhodopsin: PRPH2 ratios. In addition, the onset and severity of the disease may be influenced by the location of the mutation, as different PRPH2 domains have been implicated in dual roles during disc morphogenesis, the tetraspanin core in rim membrane curvature, and the *C*-terminal domain in ectosome release suppression [52].

ROM1 is thought to be involved in the regulation of OS disc formation. PRPH2 and ROM1 are closely associated at the disc rims in the OS. In humans, a double heterozygous disruption in both *ROM1* (a PRPH2-interacting protein) and *PRPH2* results in digenic RP [106]. While it is not clear whether defects in ROM1 alone causes RP in humans, mice with a monogenic *Rom1* disruption show signs of dominant RP. At one month of age, *Rom1* knockout OS discs are visible but appear enlarged and slightly disorganized [107]. PRs slowly degenerated, reducing the ONL by 34% at 1 year of age. In contrast, *Rom1^Rgsc1156^* mice with a heterozygous missense mutation, p.W182R, show a 55% loss of PRs at 35 weeks of age [108]. Furthermore, RD was more pronounced in homozygous mice. The degeneration in *Rom1^Rgsc1156^* mice may be a consequence of an early reduction in endogenous PRPH2 and ROM1 levels, which may interfere with PRPH2-mediated stabilization of disc outer rims.

PRCD, progressive rod-cone degeneration, is a rhodopsin-binding protein [109] that localizes to the OS disc rims [110]. Patients and canines with *PRCD^C2Y^* mutations have a slowly progressive form of rod-cone degeneration [111]. The Cys2Tyr mutation results in mislocalization of PRCD from the OS to the ONL where it is actively degraded [109]. In the PRs of mice with homozygous *Prcd^tm1Vya^* mutations, loss of PRCD results in the formation of bulging discs that do not properly flatten and in the accumulation of extracellular vesicles that originate at the OS base [112]. Interestingly, mutant PRs are able to form membrane discs and the distribution of OS proteins and light response do not appear to be perturbed. While activated microglia infiltrate the interphotoreceptor space to remove extracellular vesicles and debris, removal is insufficient and PRs undergo a very slow degeneration. Homozygous *Prcd^tm1Vya^* mice show only 36% ONL loss at 17 months [112] while in *Prcd^tm1(KOMP)Mbp^* homozygotes a similar loss is observed at 30 weeks of age [110]. Both models are knockout alleles that target the 5′ end of *Prcd* but are on different genetic backgrounds. Further investigations are necessary to determine whether gene modifiers affect progressive PR cell loss in these two models.

IFT trafficking. IFT is essential for ciliogenesis in mammals [113] and disruption of this process often leads to abnormalities in embryonic development. In the mouse, null mutations in genes encoding subunits of the IFT-A (*Ift122* [114], *Ift88* [115], and *Ttc21b* [116]) and IFT-B (*Ift172* [117], *Ift80* [118], and *Traf3ip1* [119]) complexes result in embryonic lethalities, many of which are attributable to ciliary-related disturbances in hedgehog signaling [120,121]. These findings further highlight the integral role of cilia and IFT machinery during embryogenesis.

Hypomorphic and conditional alleles have been useful for elucidating the roles of IFT components in retinal disease. The hypomorphic allele, *Ift88^Tg737Rpw^*, contains a transgenic insertion resulting in a 2.7 kb intronic deletion. Homozygous *Ift88^Tg737Rpw^* mice exhibit disorganized OSs as early as P10 and a progressive degeneration of PRs that reduces the ONL to one layer at P77 [66]. Rod-specific ablation of *Ift172* [122] leads to mislocalization of rhodopsin, RP1, and TTC21B (IFT139) and rapid degeneration of PRs. Conditional depletion of *Ift20* in M cones and mature rods both results in opsin mislocalization suggesting that proper opsin trafficking hinges on functional IFT components [123]. To gain a clearer understanding of the roles that IFT molecules play in both rod and cone PRs, additional studies using conditional models are warranted to elucidate the contributions of impaired IFT components to PR cell loss.

Lipidated protein trafficking. Lipid modification of proteins, such as prenylation or acylation, helps direct intracellular protein targeting and regulates protein activity [124]. These hydrophobic modifications help tether their protein partners to the surface of specific membranes throughout the cell, such as the ER, Golgi, transport vesicles, or plasma membranes. Improper trafficking of lipidated proteins can result in RD. RP2 is a GTPase activating protein that interacts with ARL3 to regulate assembly and movement of membrane-associated protein complexes [125]. Homozygous mice with mutations in the gene encoding RP2 exhibit a slowly progressive rod-cone degeneration [126,127]. ARL3, a small GTPase, traffics lipidated membrane-associated proteins to the rod OS [128]. Although *Arl3* knockout mice exhibit early postnatal lethality and Joubert-like features [42], mice with hypomorphic mutations survive to post-wean and display OS abnormalities as early as P9 [129]. Conditional ablation of *Arl3* in the developing retina results in the absence of cilia, and therefore PR cells are rapidly lost [42]. In contrast, depletion of *Arl3* in mature rods leads to mislocalization of lipidated OS proteins, shortened OS, and a moderate progressive PR loss. These results are consistent with roles for ARL3 in ciliogenesis during development and cargo displacement during lipidated protein trafficking.

The ciliary TZ-associated protein, RPGR, binds and directs the ciliary targeting of INPP5E [130], a phosphoinositide phosphatase that is important for ciliogenesis [131]. Ciliary localization of RPGR itself requires modification with a prenyl group, which interacts with PDE6D [130], a prenyl-binding protein first discovered as a copurifying component of cGMP phosphodiesterase 6 (PDE6) [132]. Like *Rpgr^rd9^* [133] and *Rpgr^tm1Tili^* knockout mice [134], mice with *Pde6d^tm1.1Wbae^* null mutations [135] undergo a very slow degeneration with at least 50% of PR cells remaining at 20 months of age. Rao et al. have demonstrated reduction of INPP5E in RPGR-deficient axonemal OSs [130]. Altogether, these observations validate RPGRs role in ciliary trafficking and homeostasis and suggest that other players may be involved in ciliary targeting of INPP5E.

Mutations in *Aipl1* result in early and rapid loss of PR cells (Table S1). D_50_ < 0.55 months for the four germline alleles shown in Figure 6, *Aipl1^tm1Mad^*, *Aipl1^tvrm127^*, *Aipl1^tm1Visu^*, and *Aipl1^tvrm119^* [21,39,136]. AIPL1 is a protein chaperone that mediates the folding of phosphodiesterase 6 (PDE6), a key component of the visual transduction pathway that regulates cGMP levels (see Section 5.2.2. below) [137]. AIPL1 binding is promoted by prenylation of PDE6 subunits [137]. In *Aipl1* mutants, PDE6 subunits are greatly diminished [136], providing further evidence for the importance of lipid modification in PR viability and vision.

BBSome assembly and regulation. Disruptions in the octameric BBSome complex or associated chaperonins may cause syndromic ciliopathies such as the Bardet–Biedl syndrome and McKusick–Kaufman syndrome. In mice, most gene disruptions that affect the BBSome [138] (BBS1, BBS2, BBS4, BBS7, BBIP1, TTC8, and ARL6), and its regulators (LZTFL1, MKKS, BBS10, and BBS12) result in a moderate degeneration of PRs. For instance, PRs in homozygous mice harboring gene trap or null mutations of *Bbs4*, *Bbs4^Gt1Nk^* [139], and *Bbs4^tm1Vcs^* [140] appear to progressively decline after maturation with >90% loss at 7 months of age. The delay and lack of ciliogenesis defects suggests that there may be some functional redundancy amongst components of the BBSome.

#### 5.2.2. Category 02: Visual Transduction

Overview. Mutant alleles of genes encoding proteins responsible for light detection comprise a second category of models (Category 02: Visual Transduction; Figure 1c, Table S1). The multistep phototransduction process that detects light and amplifies this signal is similar in rod and cone cells, but the specific proteins that catalyze many of the steps are often unique to each cell type [141]. Phototransduction is initiated by the response of opsin-based light-sensitive G protein coupled receptors that are covalently linked to vitamin A retinal as a cofactor. The receptor rhodopsin (RHO) is expressed exclusively in rod cells and is optimized to detect dim green light. Cone pigments that detect short or medium wavelength visible light (OPN1SW and OPN1MW, respectively) are exclusively expressed in cone cells, in some retinal regions coordinately within the same cell. These receptors constitute >90% of OS protein and are localized to the disc membranes.

Light activation of RHO or cone pigments causes the bound retinal to isomerize from an 11-*cis* to an all-*trans* configuration, ultimately leading to its release from the receptor by hydrolysis. Isomerization results in a conformational change in the protein that alters its interaction with a bound heterotrimeric G protein, transducin, activating the exchange of GTP for GDP bound to the α subunit of this protein. In turn, activated α transducin-GTP binds the inhibitory γ subunits of phosphodiesterase 6, releasing it from the α and β subunits of this complex, which are thereby activated to catalyze the conversion of cGMP to GMP. The ensuing reduction in cGMP levels in the OS closes the cGMP-gated cation channel, slowing the influx of Na^+^ and Ca^2+^ ions, which hyperpolarizes the plasma membrane of the OS and, ultimately, the entire cell. Hyperpolarization causes Ca^2+^ channels to close at the cell synapse, which leads to a decrease in the calcium-dependent release of glutamate-containing vesicles into the synapse and activates postsynaptic bipolar neurons.

The process is regulated to ensure the highest sensitivity to illumination. Following its activation, rhodopsin is quenched by the action of arrestin, which binds to bleached opsin molecules that are phosphorylated by rhodopsin kinase. Resetting of the cell following the light flash requires the formation of cGMP from GTP, catalyzed by a membrane-bound guanylate cyclase, the subsequent closing of the cGMP-gated cation channel, and the restoration of electrolyte distribution across the plasma membrane as achieved by ion pumps and transporters. Hydrolyzed retinal is passed from the OS to the RPE as part of the visual cycle (see below), where it is re-isomerized and returned to the PR cell to regenerate bleached opsin. An additional visual cycle involving Müller cells contributes to the regeneration of cone pigments.

Visual pigments. Profound effects on PR viability are observed due to mutations that affect rod cells, which represent 97% of the PR population. Mouse models bearing *Rho* alleles exhibit semidominant and recessive rod cell loss phenotypes that vary greatly in the onset and rate, consistent with the variety of possible disease mechanisms that have been proposed for RHO mutations over decades of study. For example, some missense alleles in Table S1, such as those that encode the Pro23His, Cys110Tyr, Tyr178Cys, and Cys185Arg variants [21,142,143,144,145,146] may support a hypothesis that excessive RHO misfolding in the endoplasmic reticulum induces cellular stress pathways that lead to PR cell loss [147]. Although the pathways linking misfolded RHO to cell death are not fully resolved, recent studies of the Pro23His variant in cultured cells and in rats [148] or mice [145,146] suggest that stress pathways induced by the unfolded protein response are protective, and raise the possibility that increased intracellular calcium due to ER stress may cause cell death [146]. Misfolding may also explain the partial mislocalization of RHO Glu150Lys to the IS [149]. However, in this mutant, much of the protein appears to be correctly exported to the OS, where it leads to irregularly shaped and disorganized discs, possibly due to a defect in higher-order RHO organization [149]. Pro23His RHO also disrupts the orientation of discs during their morphogenesis, possibly through similar effects on higher-order structure [150].

By contrast, the effect of the Gln344Ter variant (Table S1), which is correctly folded but includes sequence extensions at the C-terminus that interfere with export to the OS [151], as well as the graded effect of heterozygous or homozygous knockout alleles *Rho^tm1Jlem^* and *Rho^tm1Phm^* [152,153] or the premature truncation mutant Arg107Ter (Table S1), provides evidence that a steady flow of RHO to the OS is essential for PR cell viability. These observations fit an emerging view that a proteostasis network, incorporating not only cellular stress pathways but also protein trafficking and degradation, regulates the cellular protein balance to ensure viability [147,154]. According to this view, a failure to sort vesicles bearing RHO from the Golgi to the periciliary membrane, or a partial or complete loss of the protein, leads to protein imbalance in the IS. This imbalance may induce cellular stress responses and also affect the trafficking of other molecules destined for the OS, such as other phototransduction proteins, lipids, and vitamin A, resulting in cellular toxicity. Finally, the RHO Asp190Asn variant (Table S1) appears to traffic properly to the OSs but may have structural defects that lead to constitutive signaling [155], which has been linked to PR degeneration [156]. The same mechanism may account for the effect of *Rho* mutants that result in rapid degeneration upon bright illumination [157] but were not included in Table S1 due to the dependence of the mutant phenotype on an environmental perturbation (see Discussion). Future studies of these and other models may resolve or converge the many proposed hypotheses to explain RHO-associated RD.

Based on the often profound effect of *Rho* variants on rod cell viability, it might be expected that cone pigment variants would similarly cause cone PR cell loss. However, cones remain viable for more than 1.5 years in homozygous *Opn1sw^tm1Pugh^* mice, which show a 1000-fold decrease in transcript and produce no detectable OPN1SW by immunoblotting, histochemistry, or single-cell recording of light responses [158]. Likewise, cones are viable for at least 10 months in homozygous *Opn1mw^tm1a(EUCOMM)Wtsi^* knockout mice, despite an absence of OPN1MW in immunoblotting and immunohistochemical studies [159]. These studies suggest fundamental differences in the cellular sensitivity of rod and cone cells to visual pigment deficiency. They also highlight the concern that reactivity to antibodies against cone opsins or other cone cell markers may be abolished even though the cells remain viable, and therefore may not be as reliable as counting cone nuclei [160] to assess cell loss.

Transducins. Rod transducin subunits α, β, and γ (encoded by *Gnat1*, *Gnb1*, and *Gngt1,* respectively) form the heterotrimeric G protein complex that is essential for propagating the signal from light-activated rhodopsin. *Gnat1* knockout mice have attenuated rod responses and model congenital stationary night blindness (CSNB) [161]. Although slow PR loss was reported for this model, our measurement of ONL thickness at four weeks of age based on reported images yielded a value of 90% of wild type, matching the author’s value at 13 weeks [161] and suggesting an early developmental difference rather than progressive cell loss. In support of this finding, others using the same strain reported ONL thickness was 85% of wild type at eight weeks of age with no evidence of significant cell loss up to 52 weeks of age [162]. By contrast, IRD2 mice, which are homozygous for a *Gnat1^irdr^* allele predicted to yield a prematurely truncated polypeptide, exhibit significant rod PR cell loss (Table S1) accompanied by late cone cell loss and reduced rod-specific ERG responses [163]. Homozygous *Gnat1^irdr^* mice may recapitulate recessive rod-cone dystrophy, which has recently been linked to human *GNAT1* variants predicted to encode prematurely truncated proteins [164,165,166]. The *Gnat1^irdr^* allele was discovered independently in *rd17* mice at JAX, suggesting a founder effect [167,168].

*Gnb1* knockout mice have not been studied due to embryonic and perinatal lethality. However knockout alleles of the gene encoding rod γ transducin, *Gngt1^tm1Dgen^* and *Gngt1^tm1Ogk^*, result in PR loss that is more rapid than in *Gnat1* mutants [169,170]. In these strains, GNGT1 deficiency is accompanied by a 6- to 50-fold post-translational reduction of GNAT1 and GNB1, indicating a key role of the transducin γ subunit in complex assembly. *Gngt1^tm1Dgen^*-associated degeneration is rescued by heterozygous *Gnb1^Gt(prvSStrap)4B8Yiw^* mice [171], which express retinal GNB1 at 50% of wild type levels. This result suggests that the toxicity of GNGT1-deficiency is due to an excess of improperly assembled GNB1, which is targeted for degradation but exceeds the capacity of the proteasome [171]. This observation supports the proteostasis network model of PR degeneration [154].

Among genes encoding cone transducin subunits α, β, and γ (*Gnat2, Gnb3,* and *Gngt2*), only *Gnat2* alleles have been reported to cause PR loss. A progressive reduction of cone cell ERG responses and a 27% decrease in PNA-positive cells at 12 months of age in homozygous *Gnat2^tm1Erica^* mice (Table S1) is consistent with cone PR loss [172]. However, cone nuclei were not counted directly, so it is possible that cone cell loss is less pronounced than reported. The predicted GNAT2 Asp173Gly substitution in this model may alter guanine nucleotide binding [172], although how this change might cause cell loss is unresolved. Interestingly, mislocalized cone opsin OPN1MW in this model suggests endoplasmic reticulum stress, which is often associated with PR degeneration. *Gnat2^cpfl3^* mice (Table S1) show no cone cell loss for at least 14 weeks but exhibit a slow loss of rod cells [173]. In contrast to these models, a recently developed *Gnat2* knockout strain abolishes GNAT2 function without PR loss or dysmorphology in the oldest mice examined at 9 months of age [174]. Although human *GNAT1-*variants are a rare cause of achromatopsia [175], a stationary congenital colorblindness, the clinical presentation is variable and some cases are associated with a reduction in visual acuity with age [176] that may suggest progressive cone cell loss. The available mouse alleles may help to identify disease mechanisms that contribute to this phenotypic variability.

Phosphodiesterase 6. Rod phosphodiesterase 6 consists of a catalytic αβ complex encoded by *Pde6a* and *Pde6b* and two inhibitory γ subunits encoded by *Pde6g*. The control of cGMP levels by this enzyme is expected to affect both PR function and viability, as cGMP has a central role in the phototransduction cascade and PR cell metabolism [177], and elevated cGMP levels have been linked to PR cell loss [178]. Indeed, *Pde6a* and *Pde6b* mutants show depressed ERG responses at an early age and rapid PR loss with D_50_ values of 11–30 days (Figure 6, Table S1). A study of *Pde6a* mutations on the same strain background made use of an allelic series that varied in disease severity [179]. The order of disease progression due to the alleles reported in this study, *nmf282* (Val685Met; fastest) > *tm1.1Bewi* (Arg562Trp) > *nmf363* (Asp670Gly; slowest), is the same as assessed by D_50_ (Figure 6). This allelic series led to a correlation of more rapid PR degeneration with an increased number of cGMP-positive PR cells [179]. The same trend in the progression of disease in *Pde6a^nmf282^* and *Pde6a^nmf363^* mice was found earlier [180], but an opposite cGMP result was obtained, possibly due to the assessment of total retinal cGMP rather than a count of cGMP-positive PR cells [179] (a 0.1-month difference in the D_50_ of *Pde6a^nmf363^* mice measured in the two studies may reflect strain differences that might also contribute to the difference in findings). The later study also combined two alleles that matched human *PDE6A* variants to create a compound heterozygote [179], mirroring the more typical situation in human genetic disease. Further, the allelic series highlighted a non-apoptotic cell death mechanism involving calpain rather than the expected caspase-mediated apoptotic process [179]. Both elevated cGMP and calpain activation have been observed in other mouse RD models [181]. Thus, allelic series as used in these studies are informative for assessing disease mechanisms and identifying potential differences in treatment efficacy that may reflect disease severity.

Of the *Pde6b* alleles described, *Pde6b^rd1^* and *Pde6b^rd10^* have been used most extensively as PR degeneration models. *Pde6b^rd10^* disease develops later, providing a longer window of opportunity to test therapeutic efficacy (Figure 6). The *Pde6b^atrd1^* model has an even slower progression (D_50_ = 0.71) than *Pde6b^rd10^* mice (D_50_ = 0.65), which may make it more attractive for assessing the variation in treatment with disease severity (Figure 6, Table S1). Finally, loss of the inhibitory subunit in homozygous *Pde6g^tm1Goff^* mice did not lead to an expected increase in catalytic activity; instead PDE6G was found to be essential for activation and possibly stable assembly of the holoenzyme [182].

Cone phosphodiesterase 6 includes two catalytic α subunits encoded by *Pde6c* and two inhibitory γ subunits encoded by *Pde6h*. The *Pde6c^cpfl1^* mutation leads to severely reduced cone ERG response at three weeks and progressive cone PR loss with age [15] as determined by counting cone nuclei (Bo Chang, unpublished data, presented in Table S1). This model mimics achromatopsia in humans, which is sometimes accompanied by cone PR cell loss [183]. Surprisingly, *Pde6h* knockout mice show no detectable functional cone loss or degeneration, likely due to the expression of the *Pde6g* subunit in mouse cones, which may compensate for PDE6H loss [184]. Variants in human *PDE6H* cause achromatopsia [185,186] but cone cell loss has not been reported.

Cyclic nucleotide gated channels and cation exchanger. The decrease in cGMP levels resulting from PDE6 activation leads to the closing of cyclic nucleotide cation channels in the OS plasma membrane of both rods and cones. Channel closing diminishes the inward flux of Na^+^ and Ca^2+^ ions that maintain the PR cell in a hyperpolarized state. The rod protein encoded by *Cnga1* and *Cngb1* is an α_3_β_1_ heterotetramer, in which the β subunit is a long isoform, CNGB1a [187,188]. *Cnga1* mutations have not yet been described. Rod OSs of homozygous *Cngb1^tm1.1Biel^* mice yield no detectable CNGB1a or CNGA1, and rapid PR loss is observed [189]. Together with evidence that CNGA1, but not CNGB1a, is capable of self-oligomerizing in heterologous expression systems, this result suggests that CNGB1 plays a critical role in stabilizing CNGA1 for channel assembly during synthesis in the secretory pathway and/or subsequent transport to the OS. Although the mechanisms leading to PR cell loss are unknown, low intracellular Ca^2+^ may overactivate guanylyl cyclase and cause toxicity due to elevated cGMP [189].

The cone channel encoded by *Cnga3* and *Cngb3* functions as an α_2_β_2_ tetramer. Due to the absence of downstream synaptic signaling associated with channel defects, mutations in both genes result in a loss of cone ERG responses modeling achromatopsia. In addition, the alleles included in Table S1, *Cnga3^cpfl5^, Cnga3^tm1Biel^*, *Cngb3^cpfl10^,* and *Cngb3^tm1Dgen^* result in cone PR degeneration as assessed by marker analysis, although confirmation of cell loss by a direct nuclear count was lacking in some studies. The mechanism of cell death is unknown in these models, but by analogy may involve elevated cGMP as hypothesized in rods.

A critical component of phototransduction is SLC24A1 (also called NCKX1), which exports sodium and calcium ions in exchange for potassium. This activity is responsible for the decrease in intracellular Ca^2+^ upon closing of the cGMP-gated channels. Homozygous *Slc24a1^tm1Xen^* mice exhibit slow degeneration, possible due to malformation of OS discs [190].

Guanylyl cyclase and activating proteins. Photoreceptor guanylyl cyclases function as homodimers encoded by two genes in mice, *Gucy2e*, and *Gucy2f*. In the homozygous *Gucy2e^tm1Gar^* model, D_50_ was >12 months (Figure 6), indicating very slow rod PR cell loss, while cone cell numbers decreased rapidly to 33% of controls in 5 weeks [191]. Cone loss with rod preservation has been observed in Leber congenital amaurosis cases linked to variants of the human *Gucy2e* ortholog, *GUCY2D* [192]. However, *Gucy2e^tm1Gar^* mice are not considered to model this disease because rod ERG function, though diminished, is still detectable [191]. Although *Gucy2f* knockout did not cause PR cell loss, double knockout of both guanylyl cyclase genes resulted in moderate degeneration [193]. Rod and cone ERG responses were abolished in this model, suggesting that the residual function in *Gucy2e^tm1Gar^* mice was due to compensatory activity expressed from *Gucy2f*. The mechanism of PR cell loss in these models is unlikely to involve elevated cGMP as the enzymes needed for its production are ablated. The post-translational downregulation of other phototransduction proteins in double-knockout mice [193] may indicate a disruption of the proteostasis network that could explain PR cell loss.

Guanylyl cyclase activator proteins provide a feedback loop to restore cGMP levels. When intracellular Ca^2+^ is high, these proteins inhibit guanylyl cyclase; when Ca^2+^ levels are low, they switch to an activating Mg^2+^-bound conformation that promotes cGMP synthesis. This Ca^2+^-sensitive regulation permits PR cells to reestablish cGMP levels following light exposure due to lowered intracellular Ca^2+^, thereby resetting the cell for another stimulus. Double knockout of *Guca1a* and *Guca1b*, which encode the activator proteins in both rods and cones, had no detectable effect on retinal morphology up to eight months of age [194]. However, homozygous *Guca1a^tm1.1Hunt^* mice, which have a Glu155Gly missense substitution identical to one found associated with a severe dominant cone dystrophy [195], result in rapid loss of cones and subsequently rods (Figure 6, Table S1). This mutation, like others associated with the human disease, may constitutively activate guanylyl cyclase due to a defect in calcium sensing [196], leading to cytotoxic accumulation of cGMP.

Recovery from light stimuli. Mechanisms to terminate the phototransduction cascade and recover the PR cell for additional stimuli include the phosphorylation of activated RHO by a *Grk1-*encoded kinase and the binding of *Sag-*encoded arrestin to the phosphorylated RHO. The binding of SAG limits transducin access to RHO and thereby prevents further activation of transducin and downstream processes. Significantly, defects in either gene induce photoreceptor cell loss, likely due to the accumulation of excess cGMP arising from unregulated active RHO. Early studies aimed at elaborating the role of the SAG or GRK1 proteins used mice raised in the dark [197,198], as typical vivarium cyclic light–dark rearing conditions were described as leading to rapid degeneration. Subsequent studies of homozygous *Sag^tm1Jnc^* [199] or homozygous *Grk1^tvrm207^* mice [200] reveal slow PR cell loss with D_50_ > 10 months under normal rearing conditions.

#### 5.2.3. Category 03: Metabolism

Overview. Inborn errors of metabolism constitute a heterogeneous group of disorders that affect metabolic pathways due to underlying genetic defects [201] and result in abnormalities in the synthesis or catabolism of biomolecules [201,202]. Many such inborn errors of metabolism are known to be associated with PR cell loss, manifested either as a primary ocular defect or as part of a systemic disease [201]. PR cells, with their high metabolic activity, are particularly vulnerable to defects in metabolism of biomolecules such as lipids, carbohydrates, nucleotides, and proteins, which provide energy and serve many other functions described below. Additionally, since organelles such as mitochondria and lysosomes are the major sites for cellular energy production and homeostasis, defects in organellar metabolism and function are also known to cause PR degeneration. The PR cell loss associated with different metabolic diseases varies in the age of onset, severity, and rate of progression (Figure 6, Table S1) and the underlying genetic defects can be categorized based on the type of biomolecular metabolism or the subcellular location of the pathways affected.

Biomolecular metabolism: lipids. PRs are extremely rich in lipids, which make up to 15% of their cellular wet weight as compared to 1% in most other cell types [203,204]. Phospholipids and cholesterol represent 90–95% and 4–6% (*w/w*) of total lipids, respectively [205]. The major phospholipids in rod outer segments include phosphatidylethanolamine, phosphatidylcholine, large amounts of phosphatidylserine, along with small amounts of sphingomyelin, phosphatidylinositol, and phosphatidic acid [205]. It has been suggested that the phospholipids in OS membranes are metabolically active and involved in generation of physiological mediators, and changes in metabolism of glycerolipids have been associated with transduction of visual stimuli [205]. Cholesterol has been reported to modulate the function of rhodopsin, a major protein of the OS membranes, by influencing membrane lipid properties [206]. Low-density lipoproteins (LDLs) are reported to be significant suppliers of PR lipids, especially cholesteryl esters [207,208]. The OSs of PRs are particularly rich in very-long-chain polyunsaturated fatty acids (PUFA), such as docasohexaenoic acid (DHA), which is considered to be essential for visual function [209], and phospholipid-containing DHA is suggested to help in isomerization of 11-*cis*-retinal to the all-*trans* form, which is further reduced for its entry into the visual cycle [210]. Recently, DHA has also been implicated in the maintenance of OS homeostasis [211] and mediating PR cell survival [212,213].

Thus, it is not surprising that disorders of lipid metabolism cause inherited PR degeneration. For example, mouse models for mutations in the elongation of very-long-chain fatty acids-like 4 (*Elovl4*) gene are reported to show features resembling Stargardt-like macular dystrophy in humans with cone degeneration preceding that of rods [214,215]. Mutations in genes involved in phospholipid metabolism such as *Lpcat1* cause rapid PR degeneration (90% and 75% degeneration in *Lpcat1^rd11^* and *Lpcat1^rd11-2J^* alleles, respectively by 47 days) [216]. Similarly, mutations in genes involved in cholesterol biosynthesis such as *Nsdhl* [217] or in the biosynthesis and regulation of DHA-containing phospholipids, such as *Agpat3* and *Adipor1,* respectively [210], also cause PR degeneration, confirming the importance of lipids in preserving PR integrity. Since membrane phospholipid asymmetry is critical to performing various biological functions, mutations in genes important for its generation and maintenance, also lead to PR degeneration. For example, mutations in *Atp8a2,* a type of P4-ATPase that translocates and maintains phospholipid asymmetry show a 30–40% PR degeneration by two months of age [218]. Similarly, conditional inactivation of *Tmem30a*, known to be required for folding and transport of several P4-ATPases to their plasma membrane destination [219,220], also results in severe PR degeneration [221]. *Tmem30a* knockout mice exhibit a more severe phenotype compared to *Atp8a2* knockout mice, possibly because *Tmem30a* binds multiple P4-ATPases [221].

Biomolecular metabolism: carbohydrate and nucleotide energy metabolism. The retina, and in particular PRs, have a high metabolic rate [222,223] to support functions that are energetically demanding, such as phototransduction during constant illumination, maintenance of ion gradients in darkness, and performing anabolic metabolism to replace the approximately 10% of OSs that are lost every day to phagocytosis by RPE cells [223]. RPE cells also perform many energy demanding functions, such as maintenance of appropriate ionic and fluid composition in the subretinal space, uptake and conversion of all-*trans*-retinol to 11-*cis*-retinal and its transport back to photoreceptor cells, and OS phagocytosis. This high energy requirement makes the retina and RPE particularly vulnerable to functional deficits induced by deficits in energy metabolism [222]. The retina relies on blood-derived glucose and oxygen for its energy requirements. Additionally, PR cells use excess lactate obtained from Müller glial cells and convert it to pyruvate to provide energy via oxidative phosphorylation [222]. In addition to carbohydrates, the retina uses fatty acids [224] and nucleotides for its energy requirements [223].

Thus, neuronal activity and energy metabolism are tightly coupled and any mutations at the level of glucose, fatty acid or nucleotide biosynthesis can lead to PR degeneration. For example, mice lacking *Hkdc1*, which encodes a kinase found in the IS that phosphorylates glucose to glucose-6-phosphate, show 40% PR degeneration by 17 months [225]. Mice mutant for *Vldlr*, which encodes the receptor facilitating the uptake of triglyceride-derived fatty acids, show reduced cellular uptake and availability of fatty acids for energy production [224]. For some alleles of *Vldlr* (*Vldlr^m1Btlr^* and *Vldlr^tm1Her^*), more than 50% of PRs are lost by 12–14 months [226,227], with cones being affected more significantly than rods [228]. The decrease in net available energy may lead to greater cone loss, as cones have been reported to require three times more energy than rods [222]. Similarly, while in some cases, mutations in genes involved in nucleotide metabolism such as *Nampt*, show embryonic lethality [229], others such as mutation in *Nmnat1*, show severe PR degeneration by 4–6 months [230].

Biomolecular metabolism: hormones. The physiology of eye is also dependent on the action of several hormones [231]. Mouse models mutant for thyroid hormone metabolizing genes, such as *Dio3*, which is important for local amplification of triiodothyronine (T3), show selectively detrimental effects on cone cells [232]. This confirms the proposed role of thyroid hormone signaling in regulating cone viability and cone opsin expression [232,233]. Melatonin, a hormone that plays a role in sleep patterns, is known to have protective role against oxidative stress and apoptosis, and regulates retinal circadian rhythms [234]. A mouse model, mutant for the melatonin hormone receptor *Mtnr1a*, shows very slow PR degeneration (25% in 18 months) [235].

Biomolecular metabolism: oxidative stress. The eye is constantly subjected to oxidative stress due to daily exposure to light, atmospheric oxygen, and high metabolic activities [236]. Reactive oxygen species (ROS) are derived from diatomic oxygen and processes such as mitochondrial respiration that form superoxide anion radicals, toxic bis-retinoids that undergo photo-oxidation, and lipids, such as PUFAs, that undergo peroxidation [237]. Having unpaired electrons confers a great degree of ROS reactivity that can damage biomolecules such as DNA, lipids and proteins, and organelles including mitochondria and lysosomes [238,239], thereby impairing their biological functions [203,236]. Compared to other cells, non-proliferative postmitotic cells such as PRs and RPE cells are particularly sensitive to oxidative damage due to the apparent absence of a DNA damage detection system [240,241,242].

Under physiological conditions, cellular redox homeostasis is maintained by a balance between ROS generation and antioxidant systems [236]. Antioxidant enzymes such as *Sod1, Sod2,* and *Gpx4* are known to play a major role in ROS scavenging and changes in their expression or activity or both are reported to cause increased oxidative stress and are associated with diseases such as age-related macular degeneration (AMD) [243]. For example, mutations in the *Sod1* gene, encoding a cytosolic Cu-Zn superoxide dismutase that catalyzes the conversion of superoxide to hydrogen peroxide, are known to cause PR degeneration [244]. *Sod2,* which encodes a mitochondrial Mn superoxide dismutase, is required for survival and mutations in this gene lead to embryonic lethality [244,245]. Genes such as *Nxnl1* and *Nxnl2*, known as rod-derived cone viability factors are also suggested to have antioxidant function and show cone degeneration when mutated [246,247], with *Nxnl1* also showing a progressive rod cell loss [246]. Similarly, a mouse model for loss of *Ttpa*, coding for a protein that transports vitamin E, which is known to have antioxidant function, also shows 40% PR degeneration by 20 months [248].

Organellar metabolism: lysosomes. The lysosome, a subcellular organelle is critical for performing several vital functions such as degradation of extracellular and intracellular material, nutrient sensing, energy metabolism, and maintaining cellular homeostasis [249]. Lysosomes contain a wide variety of hydrolytic enzymes that enzymatically degrade biomolecules such as polysaccharides, lipids, etc. [250]. Defects in lysosomal function results in lysosomal storage disorders, a group of inherited metabolic disorders sharing a common biochemical feature of accumulating incompletely degraded metabolites within the lysosomes. Lysosomal storage disorders are generally classified by the composition of the material accumulated within them and often differ depending on the lysosomal proteins affected, which reflect different cell biological processes that are affected but terminating in a similar pathology of reduced clearance of metabolic aggregates.

RD is an early consequence of lysosomal storage diseases, especially in neuronal ceroid lipofuscinoses (NCL) [251], also called Batten disease, an early-onset neurodegenerative disease with other systemic features such as dementia and epilepsy [252]. NCL may be caused by disruption of genes encoding lysosomal enzymes (*Ppt1* and *Cln5*) and membrane proteins (*Mfsd8*) as well as ER membrane (*Cln6* and *Cln8*) and secretory pathway (*Grn*) proteins, and is characterized by a common lysosomal accumulation of ceroid. Similar to the early retinal phenotype reported for most human NCLs, most mouse models for NCL disease show an early onset of PR degeneration, beginning at 1 month of age and showing greater than 60% degeneration by 6–9 months [253,254,255,256]. Additionally, similar to the adult-onset reported for mutations in human GRN, the mouse model for loss of *Grn* also shows a late onset PR degeneration by 12 months [257].

Mouse models for other lysosomal disorders, namely, mucopolysaccharidosis and mucolipidosis due to mutations in lysosomal proteins required for the breakdown of glycosaminoglycans and enzymes required for phosphorylation of glycoproteins, respectively, also develop PR degeneration. For example, mouse models for mucopolysaccharidosis with a mutation in *Naglu* present with a slowly progressive rod-cone degeneration [258], and for mucolipidosis with a mutation in *Gnptab* develop a severe PR degeneration with complete PR loss by 10 months [259].

The lysosome receives materials for degradation via two major pathways, autophagy and phagocytosis. Phagocytosis has an important function in maintaining retinal health since 10% of the OSs are phagocytosed daily by the RPE cells to dispose of waste such as photo-oxidative products while retaining and recycling useful contents back to the PR cells [260]. Phagocytosis by RPE requires its own machinery for processes such as recognition (e.g., *Cd36*), engulfment (e.g., *Mertk*), and degradation (lysosomal enzymes) of the extracellular material. Disruption of the phagocytic machinery due to absence/mutations in proteins involved in the phagocytic pathway, therefore, have severe consequences for PRs and can lead to PR cell death. Mouse models for mutations in genes involved in phagocytosis such as *Mertk*, *Cd36,* and *Rab28* show PR degeneration with the loss of *Mertk* showing a more severe phenotype (>80% degeneration by 60 days for *Mertk^tm1Grl^* and *Mertk^tm1Gkm^*) [261,262] than loss of *Cd36* (17% degeneration at 12 month) [263], and the model for *Rab28* loss showing a more cone-specific response [264].

Autophagy is another lysosome-mediated degradation process essential for maintaining cellular homeostasis [265]. Autophagic flux, the complete dynamic process of autophagy, includes multiple steps involving the formation of phagosomes and autophagosomes, autophagosome fusion with lysosomes, the degradation of the intra-autophagosomal contents, and recycling [266]. Thus, both lysosomal function and autophagy are interconnected wherein disruption of the hydrolytic functions of lysosomes impairs autophagic flux and, conversely, lysosomal function requires normal flux through autophagy [267,268]. In the retina, autophagy plays a dual role: promoting cell survival against harmful stress, and cell death. High basal autophagic levels are maintained in RPE and PR cells. RPE cells being post-mitotic phagocytes are not self-renewing; the autophagy of intracellular components is therefore essential for a normal cellular function of the RPE [265]. In PR cells, autophagy occurs during various cellular activities such as OS degeneration [269], rhodopsin protein expression [270], visual cycle function, and PR apoptosis [271]. Mouse models of conditional inactivation of autophagy genes such as *Atg5*, *Atg7*, and *Rb1cc1* in RPE cells show that these genes are indeed important for survival of the animal and show PR degeneration.

Organellar metabolism: mitochondria. Mitochondria, often referred to as “the powerhouse of the cell”, are the major site for cellular energy production in the form of ATP via oxidative phosphorylation. They also perform other important functions such as ROS generation and scavenging, calcium regulation, steroid, and nucleotide metabolism, regulation of intermediary metabolism, and initiation of apoptosis [272]. Oxidative phosphorylation is carried out by the mitochondrial respiratory chain, which consists of five complexes located along the inner mitochondrial membrane. These complexes, in an intricately organized series of biochemical events, synthesize ATP from ADP in response to cellular energy demands. A large number of mitochondria are present in the rod and cone IS and in RPE cells. The total surface area of the inner mitochondrial membrane in cones is 3-fold greater than in rods, presumably accommodating more respiratory chain enzymes to generate more ATP. Cones require more ATP than rods as they do not saturate in bright light and use more ATP/sec for light transduction and phosphorylation [222].

Defective cellular energy production due to abnormal oxidative phosphorylation in mitochondria can therefore lead to PR degeneration. A mouse model for the Leu122Pro mutation of OPA3, a protein hypothesized to be important for maintaining the inner mitochondrial membrane, is reported to cause a multisystemic disease characterized by severely reduced vision, loss of ganglion cells and PR degeneration (by 50%) at 3–4 months of age, a much more severe progression than observed in humans [273]. Similarly, a mouse model for a mutation in the gene for NAD-specific mitochondrial enzyme isocitrate dehydrogenase 3 (*Idh3a*), catalyzing the rate limiting step of TCA cycle, also causes an early and severe PR degeneration (more than 90%) by 90 days [274].

Extra-mitochondrial components of the tricarboxylic acid cycle and oxidative phosphorylation machinery have been localized to the rod OS [275]. It has been hypothesized that perturbation of this machinery results in excess ROS production, leading to PR cell death due to oxidative stress [275,276,277]. Mutations in a subset of mouse RD models in Table S1 alter genes (*Mpc1, Opa3, Idh3a, Impdh1,* and *Oat*) that encode mouse homologs of mitochondria-associated proteins identified in bovine rod OS [275]. Of these, only IDH3A is directly involved in cellular energy production [274]; the others may influence oxidative phosphorylation or the TCA cycle indirectly, possibly altering the generation of ROS. It may be of interest to determine whether PR cell loss in these mouse models correlates with an altered distribution of extra-mitochondrial oxidative phosphorylation proteins in the rod OS [278], or an increased ROS production, which can be measured in retinal explants [279].

Organellar metabolism: peroxisomes. Peroxisomes are subcellular organelles with various catabolic and anabolic functions such as catabolism of long chain fatty acids and biosynthesis of DHA and bile acids [280]. Several childhood multisystem disorders with prominent ophthalmological manifestations have been ascribed to the malfunction of the peroxisomes, either at the level of peroxisomal biogenesis (PBD) or single enzyme deficiencies [281]. While little is known about the metabolic role of these organelles in retina, studies have shown the presence of peroxisomes in nearly all layers of retina and RPE, albeit with differential expression of lipid metabolizing enzymes, suggesting different functions in different cell types [282]. For example, Zellweger spectrum disorder (ZSD) is a disease continuum known to result from inherited defects in *Pex* genes essential for normal peroxisome assembly. Mice homozygous for the G844D point mutation in *Pex1* show a decreased ERG response and loss of cone PRs (up to 80%) by 22 weeks, recapitulating the abnormal retinal function phenotype in ZSD patients with mild disease [283]. The retinal pathology in such disorders suggests the importance of peroxisomes in maintaining retinal homeostasis and function.

#### 5.2.4. Category 04: Visual Cycle and Retinoids

The visual cycle reisomerizes vitamin A retinal that has been released from visual pigments in PR cells, allowing regeneration of the bleached pigments and the subsequent detection of additional light stimuli. The process is catalyzed by enzymes located in PR and RPE cells, so the retinoid intermediates in the process must be transported between them. Mutation of genes involved in the visual cycle pathway cause PR degeneration, in most instances with a moderate to slow progression depending on the allele and the genetic background. Most *Rpe65* mutant alleles show moderately slow PR cell loss (D_50_ = 7–11 months) [284,285,286,287,288]. Allelic effects are observed in models bearing missense mutations, *Rpe65^tm1Lrcb^* [289] or *Rpe65^tm1.1Kpal^* [290], which cause slower progression than observed in *Rpe65^tm1Tmr^* knockout mice [285,286,287,288]. *Abca4^tm1Ght^* on the BALB/c strain, which also carries a homozygous *Rpe65* Leu450Met mutation, show a late-onset PR degeneration with 40% loss by 11 months of age [291]. By contrast, the same *Abca4^tm1Ght^* mutation on a 129S4/SvJae background results in abnormal thickening of Bruch’s membrane but normal ONL nuclei count and thickness [292]. Several visual cycle mutant alleles have other retinal abnormalities but normal ONL nuclei/thickness. For example, *Abca4^tm1.1Rsmy^* causes only autofluorescence and A2E accumulation [293] and *Abca4^tm2.1Kpal^* on C57BL/6*129Sv leads to a RPE defect but normal ONL nuclei count and thickness [294]. In addition, PR degeneration in *Abca4* mutants can be induced by light exposure [295] or through interaction with other genes such as *Rdh8* [296,297,298]. The *Lrat^tm1Kpal^* mutation on a 129S6/SvEvTac*C57BL/6J background results in mild PR degeneration, with <10% loss at 4–5 months [299]. However, a 35% decrease in rod OS length was also reported in this model, indicating the importance of the visual cycle for OS maintenance. Another allele, *Lrat^tm1.1Bok^*, showed a similar loss of rod OS length and 18% PR degeneration at 6 months of age [300]. The *Rbp3^tmGil^* mutation results in the most rapid PR cell loss in this category (D_50_ = 0.79 months), possibly attributable to an early developmental role of the protein [301]. The *Rbp4^tm2Zhel^* congenic mutation on C57BL/6J showed 20% PR cell loss in some peripheral areas and 10% in the central retina an age of 40 weeks [302]. Mutations in two genes that play a role in retinoid uptake in the eye also result in PR cell loss. The *Rtbdn^tm1.1Itl^* allele causes a slow degeneration with a 20% and 37% loss of PR nuclei at 240 days of age in heterozygotes and homozygotes, respectively. *Stra6^tm1Nbg^* mice exhibit a normal number of rod PR nuclei but significant cone PR cell loss as detected by the cone-specific marker peanut agglutinin [303]. PR cell loss in *Stra6^tm1.1Jvil^* mice was more pronounced with vitamin A restriction [304].

#### 5.2.5. Category 05: Synapse

PRs absorb light that passes through the anterior portion of the eye and convert the light to electrochemical signals that are transmitted through the neuroretina via synaptic connections to the optic nerve and visual cortex [305]. Thus, synapses, necessary for proper cell-to-cell communication, are critical for vision. Discussion of the complexity of PR synaptic development and function is reviewed in [306,307,308], and is beyond the scope of this review. Suffice to say that mutations in many of the components of synapses, such as presynaptic exocytotic proteins, endocytic proteins, calcium channels, postsynaptic receptors, and associated elements, must be properly organized to mediate transmission of signals, or can lead to visual problems [307]. It is interesting to note that disruption of some synaptic components of the secondary neurons (e.g., GRM6, GPR179, TRPM1, NYX, GNAO1, GNB5, and GNB3), while affecting function as assessed by ERG response, does not normally lead to PR degeneration [309]. This is also true of some presynaptic proteins, such as dystrophin [310] or dystroglycan [311]. However, disruption of some synaptic genes such as *Ache, Cabp4, Cacna1f, Cacna2d4,* and *Unc119* does lead to PR degeneration. For example, a null allele of *Ache* [312], causes a 50% loss of PR nuclei between 1.5 and 2 months and >80% by 6–8 months. Although it was initially determined that the ACHE protein played an important role in hydrolyzing acetylcholine at synapses, its isoforms are now recognized to have far reaching structural functions [313]. Additionally, it has been shown that the loss of secondary neurons in the null allele model is likely to cause secondary PR cell loss [312]. Null or spontaneous alleles of synaptic genes that encode subunits of calcium channels that regulate the release of neurotransmitters, and the development and maturation of exocytic function of PR ribbon synapses, *Cacna1f* [314] and *Cacna2d4* [315,316], respectively, show a slower rate of degeneration. By two months, there are approximately 10–25% of PR nuclei that have degenerated. CABP4, a protein that regulates calcium levels and neurotransmitter release at PR synapses, and modulates CACNA1F and other calcium channel activity shows a similar rate of PR degeneration of 10–25% loss at 2 months [317]. UNC119, which localizes to PR synapses (and IS) and is hypothesized to play a role in neurotransmitter release, also leads to a relatively late onset, slower rate of PR degeneration [318]. Interestingly, Haeseleer has described an interaction between synaptic genes *Cabp4* and *Unc119* [319]. It is likely that other synaptic proteins will also lead to PR degeneration through either a primary or secondary effect and that the interactions among the synaptic proteins will play a significant role in determining the relative rate of the degenerative process.

#### 5.2.6. Category 06: Channels and Transporters

Ions such as sodium, potassium, and chloride, play important roles in the visual circuitry [320]. Their intracellular concentrations and movements within the cell, and between cells and the environment are exquisitely regulated by channels and transporters. Due to the importance of maintaining appropriate levels of these ions for proper function and maintenance of PRs, it is not surprising that disruption of these genes can lead to PR degeneration. Members of the ClC family of chloride channels, such as *Clcn2, Clcn3,* and *Clcn7,* show particularly early and significant PR degeneration. Compared to other channels in this section, they appear to have an enriched expression in the RPE. A 50% PR cell loss can be seen as early as 14–16 days in certain models with disruptions in these genes [231,321,322,323]. Indeed, rapid progression of PR cell loss is observed in mice carrying any of the following alleles: *Clcn2^nmf240^*, *Clcn2^tm1Tjj^*, *Clcn3^tm1Lamb^*, *Clcn3^tm1Tjj^*, *Clcn7^tm1Tjj^*, *Clcn7^tm2Tjj^*, and *Clcn7^tm4.1Tjj^* [231,321,322,324,325,326,327]. A similar rapid and complete loss of PRs is seen when *Atp1b2*, a Na^+^/K^+^-ATPase thought to play a role in cell adhesion, is inactivated [328]. A targeted mutation in *Slc6a6,* which encodes a taurine/beta-alanine transporter, also leads to rapid, complete degeneration [329]. In contrast, inactivation of the bicarbonate, amino acid transporters, and Na^+^/H^+^ exchangers, *Slc4a7, Slc7a14,* and *Slc9a8* results in a later onset, but still severe PR degeneration [330,331,332]. Mouse models bearing mutations affecting BSG, a protein that has a role in targeting monocarboxylate transporters such as SLC16A1 to the plasma membrane, or REEP6, which mediates trafficking of clathrin-coated vesicles from the ER to the plasma membrane at outer plexiform layer sites enriched for synaptic ribbon protein STX3, also fall in this latter late onset/severe category [333,334]. *Asic3*, an acid sensing Na^+^ channel, *Clcc1*, an intracellular chloride channel, and *Slc7a14*, an intracellular arginine transporter, all cause moderately slow degeneration when mutated [331,335,336]. Slowly progressive PR cell loss is also caused by mutation of *Mfsd2a*, which encodes a sodium-dependent lipid transporter responsible for maintaining a high DHA concentration in the retina is important for OS homeostasis, as discussed in Category 03 [337]. More data are needed to see if sensing and intracellular channels/transporters generally have milder phenotypes.

#### 5.2.7. Category 07: Adhesion and Cytoskeletal

Proper structure of the retina is developed through protein interactions between cells and within cells. The spatial and laminar organization of the retina is maintained through junctional interactions between cells that impart mechanical support to maintain retinal architecture, a means for bidirectional communication (e.g., extracellular changes to the cell and from the cell to its environment), and together can form diffusion barriers. Within cells, cytoskeletal architecture is maintained through interactions of proteins with actin, intermediate filaments, and microtubules that serve to maintain cell morphology and polarity, and as discussed elsewhere, intracellular trafficking, contractility, motility, and cell division. Examples of disrupted proteins that lead to gaps between cell layers, presumably through aberrant adhesion, are mutations in *Adam9* and *Rs1*. The null allele of *Adam9*, a single pass transmembrane protein with disintegrin and metalloprotease domains that has been shown to interact with a number of integrins [338], leads to aberrant adhesion between the apical processes of the RPE and OS and to late onset PR degeneration [339]. Likewise, mutations in RS1, a protein with a discoid domain, which has been implicated in cell adhesion and cell–cell interactions [340] lead to a splitting of the inner retinal layer and progressive PR loss [341,342,343]. RS1 binding to phospholipids on the membrane surface, together with other proteins [340] may provide a stabilizing scaffold that is important in cell–matrix, cell–cell, and cytoskeletal organization.

CRB1 and its interacting partners, such MPP3, MPP5, and PARD6A, have been shown to be important in establishing proper retinal lamination presumably through their essential roles in establishing cellular apical basal polarity [344]. A primary defect of a disruption in CRB1 is the fragmentation of the outer limiting membrane [345,346]. As reviewed previously [344,347], the outer limiting membrane consists of adherens/tight junctions formed in part by the CRB1 complexes between Müller glia and the rod or cone IS that form a diffusion barrier. Loss of components of the CRB1 complexes (CRB1-MPP5-PATJ, CRB1-MPP5-MPDZ, and CRB1-PARD6A-MPP5-MPP3/MPP4) leads to lamination defects with formation of rosettes and a progressive loss of PRs. Although yet to be reported, it is likely that mutations in PATJ, PARD3, MPDZ, and MPP4 will lead to similar disease phenotypes, as a reduction in ERG response in MPDZ mutants [348] and a reduced ERG with abnormal retinal morphology have been indicated for PARD3 mutants [41,43].

Equally important to the function and maintenance of the retina are the intracellular components that make up the cytoskeletal cell structure. Disruption of proteins that interact with actin intracellularly have been shown to lead to PR degeneration. For example, CDC42, a small GTPase that is a key regulator of actin dynamics [349] leads to an early onset, progressive PR degeneration when disrupted. Models caused by mutations in FSCN2, an actin crosslinking protein [350,351], and by a hypomorphic variant of CTNNA1, a protein that coordinates cell surface cadherins with the intracellular actin filament network [352], show slow-paced PR cell loss. The proper localization of organelles within the cell is also mediated by the cytoskeletal architecture and can have an untoward effect when disrupted. For example, SYNE2, a nuclear outer membrane protein that binds to F-actin, tethers the nucleus to the cytoskeleton and is necessary for the structural integrity of the nucleus [353,354]. Without it, early onset, moderately paced PR cell loss occurs.

#### 5.2.8. Category 08: Signaling

Molecules such as growth factors/cytokines, hormones, neurotransmitters, and extracellular matrix proteins, or alternatively, mechanical stimuli, are examples of signals used to communicate environmental changes to the cell. Surface or intracellular (e.g., nuclear) receptors recognize the signals and effect changes within the cell, often setting in motion amplifying transduction cascades that mediate responses such as activation or inhibition of protein activity or migration to different cellular localizations. Further, signals can also be transmitted from the cell to other cells, for example, through neurotransmitters. Since intra- and intercellular communication is crucial for the proper development or function of cells, it is not surprising that a large number of mutations in cellular signaling lead to defects in retinal development, which in turn affects PR survival. For example, vascular development is affected in mutants bearing disruptions in *Fzd5, Lrp5, Ndp,* and *Tspan12*—all components of the Wnt signaling pathway. Integral membrane frizzled receptors, of which they are 10, together with coreceptors, LRP5 and LRP6, mediate canonical Wnt signaling [355]. Thus, conditional *Fzd5* null mutants develop microphthalmia, coloboma and persistent fetal vasculature, and late-onset progressive RD [356] and *Lrp5* mutants exhibit similar vascular and retinal phenotypes [357]. Mice that are null for NDP, a ligand for FZD4, exhibit delayed retinal vasculature development, retrolental masses, disorganization of the ganglion cell layer, and occasionally focal areas of ONL absence at later stages of the disease [358]. TSPAN12, mediates NDP-FZD4-LRP5 signaling in the retinal vasculature, where it localizes, and a mutation leads to vascular defects that phenocopies disruptions in *Ndp*, *Fzd4,* and *Lrp5*, and at 3 months exhibits a 50% loss of PRs [359]. In all of these models, it is likely that the loss of PRs is caused by the aberrant retinal vasculature having secondary effects on PRs. A review by Hackam suggests that Wnt signaling may affect the apoptotic pathway and neurotrophin release, dysregulation of which may affect PR survival [360]. MFRP, which bears a CRD domain shared by all frizzled proteins, also leads to PR degeneration when disrupted [361,362], as does the human knock-in allele, p.S163R, of its bicistronic partner, CTRP5 [363]. The exact role or function of either protein has yet to be fully elucidated.

Like the frizzled-associated proteins whose pathological effects on PRs are likely to be mediated through an aberrant retinal vasculature, other signaling molecules, *Ptpn11* and *Fyn*, appear to mediate their effects on PRs through another cell type as well, in this case, Müller glia cells, and PRKQ through the RPE. A *Six3-cre* mediated conditional knockout of *Ptnp11* [364] leads to altered ERK and MAPK signaling in Müller glia and alteration in their adhesive capabilities. FYN, a Src-kinase membrane associated tyrosine kinase, localizes to Müller glia cells, and FYN deficiency leads to altered adhesion properties of Müller cells and retinal dysmorphology [365]. PRKCQ, a serine threonine protein kinase, which localizes to the lateral surface of the RPE cells, causes a reduction in adhesion between the apical processes of the RPE and OSs when it is disrupted. The reduction in adhesion may be responsible for the retinal detachment and subsequent PR loss observed in this model [366].

The family of PI3Ks or phosphoinositide 3-kinases, made up of catalytic and regulatory subunits, function to phosphorylate the inositol ring of phosphatidylinositol and thereby regulate growth, proliferation, differentiation, motility, survival, and intracellular trafficking. For example, it mediates insulin-stimulated increase in glucose uptake and glycogen synthesis and responds to signals such as FGFRs and PDGFRs. Conditional knockouts of *Pik3cb*, encoding a catalytic subunit [367] and *Pik3r1*, encoding a regulatory subunit [368], using the cone-specific CRE, Tg(OPN1LW-cre)4Yzl, lead to progressive cone PR loss. IRS2, necessary for the integration of signals from insulin and IGF1 receptors, causes an early-onset, moderately paced PR loss [369]. Additionally, a targeted conditional allele of PDGFRB developed diabetic retinopathy like features with angiogenesis, proliferative DR-like lesions, pericyte drop out, and eventual PR loss [370]. Disruption of MAP3K1, a serine/threonine kinase, which participates in the ERK, JNK, and NF-κB signaling pathways, leads to retinal laminar and vascular defects, aberrant RPE, and PR cell death [371]. SEMA4A, a transmembrane protein, also causes PR loss, most probably through its effects on endosomal sorting [372].

### 5.2.9. Category 09: Transcription Factors

In mice, cone and rod PRs are born and develop between approximately E12 and P0, and approximately E13.5 and P7, respectively, from the same multipotent retinal progenitor cell (RPC) pool [373]. PR development, orchestrated by a network of transcription factors, is divided into five phases: proliferation of multipotent RPCs, restriction of RPC competence, cell fate specification, expression of genes important for PR function, and finally, PR structural maturation [374,375]. RB1 and E2F1 function by controlling the G1 to S phase transition in the cell cycle; RB1 plays an inhibitory role until activated by phosphorylation, balancing cell proliferation and cell fate specification [376]. OTX2 is critical for fate determination, while CRX is necessary for terminal PR differentiation and acts at different steps in PR development. Transcriptional factors important for rod PR subtype specification include RORβ, NRL, and NR2E3, and for generation of the cone subtypes, TRβ2 and RXRγ [374,375]. Further, transcription factors regulate the expression of other transcription factors in the network (e.g., CRX interacts with *Nrl*, *Rorb,* and *Mef2d*, to name a few, to mediate rod differentiation, cone differentiation, and proteins necessary for the maturation of the PR, respectively).

The importance of transcription factors in retinal development has been explored in many studies resulting in a number of mouse models with different disease phenotypes (MGI JAX). In many cases, disruption of transcription factors, especially those affecting earlier phases of PR development lead to a reduction in the total number of retinal cells generated. We have only included within this category those disrupted transcription factors that eventually lead to PR degeneration. Interestingly, the onset of degeneration of the PR transcription factor models is highly variable—14 days to 2 months of age—and appears to be dependent upon the method used to generate the model and possibly background strain, as variation of severity and onset differs among different models of the same gene. Interestingly, the *Crx* models provide a series that recapitulate the clinical diagnoses of autosomal dominant cone-rod dystrophy, Leber congenital amaurosis, and late-onset dominant retinitis pigmentosa. *Crx^Rip^* heterozygotes showed 34% degeneration at five weeks, compared to mice homozygous for the mutation, which reached 55% degeneration at the same age [377]. *Crx^tm1.1Smgc^* was also noted to have a heterozygous disease presentation more similar to a cone-rod dystrophy, while the homozygous mutant presented with a disease phenotype similar to Leber congenital amaurosis with 70% loss of PRs at one month of age [378].

Other transcriptional factors necessary for the proper maturation of the PRs, such as, MEF2D, shown to be important in regulating transcription of OS and synaptic proteins [379], or NRF1 [380], important in mitochondrial biogenesis also develop PR degeneration when disrupted. Finally, there are transcriptional factors that are important in the development or function of supporting cells such as ONECUT1 for horizontal cells [381] and MITF for RPE and/or choroidal melanocytes [382], which affect PR survival when disrupted.

### 5.2.10. Category 10: DNA Repair, RNA Biogenesis, and Protein Modification

Among the many disrupted genes that lead to PR degeneration, several instances have been documented in genes necessary for producing fully functional proteins, from transcription through post-translational modification. Defects in these genes are likely to impact the function of many other genes that they act upon, and hence, have a greater effect. Since they play a central and basic role, when disrupted they often lead to prenatal lethality in mice, and the adult phenotype is unknown unless a conditional knockout or hypomorphic allele is generated. For example, disruption of DNA repair genes such as *Ercc1*, RNA splicing genes such as *Prpf3, Prpf6, Prpf8, Prpf31*, and *Bnc2*, and miRNA processing genes, *Dicer1* and *Dgcr8* are prenatal lethal in a homozygous state [34,383]. In contrast, homozygous null alleles of *Bmi1* [384] and *Msi1* [385], both involved in repression of regulatory genes in embryonic development, are viable, suggesting potential compensatory mechanisms for the functional loss of these genes. Thus, germline, conditional or hypomorphic models were considered in this category.

Review of genes in this category suggested that a DNA damage response network to ensure transcription in the face of DNA lesions might be required for PR cell maintenance. DNA lesions, such as pyrimidine dimers, interstrand crosslinks, or double-strand breaks (DSBs), are induced by many mechanisms that include UV radiation or free radicals. Repair of such damage is essential for DNA replication and, of particular importance for long-lived post-mitotic neuronal cells, transcription [386,387,388]. Proteins encoded by *Bmi1, Dgcr8, Dicer1, Elp1, Ercc1, Ercc6, Msi1, Sirt6, Top2b, Ubb,* and *Uchl3* are known to participate in the DNA damage response [387,389,390,391,392,393,394,395,396,397,398], some in transcription-coupled DNA repair. For example, BMI1 represses transcription at sites of UV-induced DNA damage to allow repair [389]; ELP1 is a required component of the Elongator complex [399], which couples RNA polymerase II to an alkyladenine glycosylase that initiates base excision repair [392]; ERCC6 promotes DSB repair in actively transcribed regions by displacing RNA polymerase from the lesion site [387], and DGCR8 interacts with both RNA polymerase II and ERCC6 to mediate transcription-coupled nucleotide excision repair of UV-induced DNA lesions [390]. Intriguingly, topoisomerase TOP2B, which creates DSBs during transcriptional activation [396], has been identified as a key regulator of transcription during the last stages of postnatal PR development [400]. Thus, DSBs in PR cells may arise in part from transcriptional activation of genes that encode components destined for the OS. Additionally supporting the importance of DNA repair to PR maintenance, Category 01 gene *Atr* encodes a master regulator of the DNA damage response that has surprisingly been linked to retinal degenerative disease and localized to the cilium [401]. Further, Category 03 gene *Nmnat1* encodes an enzyme that synthesizes nicotinamide adenine dinucleotide in the nucleus, which may regulate the large-scale polyADP-ribosylation of protein targets at sites of DNA damage [402]. Mutations in the genes encoding these proteins all result in PR cell loss [230,384,385,400,401,403,404,405,406,407,408,409,410,411]. Mutations in five of these genes as included in Figure 6 (*Cwc27, Ercc1, Ercc6, Sirt6,* and *Ubb*) caused moderate to slow progression of PR cell loss (D_50_ ≥ 2 months), consistent with a steady accumulation of unresolved DNA damage with age. The rapid PR cell loss observed in *Atr^tm1Ofc^* mice (D_50_ = 13 days) may reflect its direct involvement in OS development [401] in addition to the DNA damage response.

Due to the high percentage of alternatively spliced genes in the human retina [412,413], it is not surprising that mutations in mRNA splicing genes: *PRPF3, PRPF4, PRPF6, PRPF8, PRPF31, PDAP1,* and *BNC2* have been shown to lead to PR degeneration in humans [3]. In fact, in human retinal disease, 14% of disease genes are categorized as playing a role in RNA metabolism [383]. Interestingly, heterozygous humanized alleles of *PRPF3* and *PRPF8* and the null allele of *Prpf31* in mice do not recapitulate PR degeneration observed in humans but rather exhibit late-onset RPE degeneration [414]. In contrast, a hypomorphic allele of the mRNA splicing gene, *Cwc27*, with reduced viability, does lead to moderate onset PR degeneration [415]. The differences observed among species require additional studies to unravel the complexities that govern genetic interactions.

Post-translational modification, which occurs by adding modifying molecules to amino acids or removing or altering these modified amino acids, is important for proper folding, transport/trafficking, localization, function, regulation, and/or degradation of proteins. Examples of post-translational modifications include phosphorylation, glycosylation, acetylation, ubiquitination, sumoylation, methylation, and lipidation [416]. Kinases that affect activity by mediating phosphorylation states are described elsewhere, however, post-translational modification genes affecting glycosylation and lipidation/prenylation are prominent among those that lead to PR degeneration. For example, the encoded proteins of *Fkrp*, *Large1, Pomt1,* and *PomgnT1*, necessary for the glycosylation of alpha-dystroglycan, essential for formation of the dystroglycan complex and for proper retinal lamination, lead to moderate rates of PR degeneration when disrupted. Prenylation is critical for proper trafficking and localization of retinal proteins. Of the three genes important in the prenylation and postprenylation processes, conditional loss of *Rce1* leads to an absence of phosphodiesterase subunits PDE6A, PDE6B, and PDE6C from the rod OS, probably due to a failure to prenylate one or more of these proteins [417]. By contrast, ablation of *Icmt* does not appear to affect phosphodiesterase transport but rather results in lowered levels of prenylated proteins GNAT1, PDE6G, and GRK1 [418], which are essential PR proteins. The null mutation of farnesyl-diphosphate farnesyltransferase 1, which adds a farnesyl group to the cysteine of the CAAX amino acid motif is prenatal lethal, but as a conditional tissue specific knockout may result in the same PR effects.

Two additional types of post-translational modification involve glycylation and glutamylation of proteins essential for normal connecting cilia function. Disruption of *Ttll3*, encoding a protein-glycine ligase necessary for glycylation of tubulin, results in an absence of glycylation in PR cells, shortening of the connecting cilia, and slow PR cell loss [419]. Interestingly, PR tubulin glutamylation increased in *Ttll3* mutant mice. TTLL5, tubulin tyrosine ligase like 5, adds glutamate residues on proteins. Sun et al. [420] reported that *Ttll5* disruption leads to late onset, slowly progressive PR cell loss that phenocopied retinal disease observed in *Rpgr* mutants. Perhaps this is not surprising as these investigators determined that TTLL5 glutamylates RPGR, a modification that is necessary for normal RPGR function in the PR cilium. *Agtpbp1* encodes a metallocarboxypeptidase that deglutamylates target proteins. Its disruption in *pcd* mutants leads to abnormal tubulin glutamylation [419] and an accumulation of vesicles in the interphotoreceptor space [421], indicating the importance of proper post-translational modification for PR survival.

### 5.2.11. Category 11: Immune Response

As resident immune cells, microglia survey the retina constantly, presumably with the goal of removing unwanted debris and responding to damage arising from environmental and/or genetic stressors. They respond to damage by eliciting various responses that can range from regenerative to inflammatory depending on the type of injury. Thus, although microglia are unlikely to be instigators in RD, it may well be the case that microglia influence the severity of responses to ocular damage depending on mutations. Mutations in several genes central to the immune system lead to PR degeneration in mouse models. *Aire^tm1.1Doi^* show early onset PR degeneration with 20% of ONL thickness loss at 10 weeks with rapid progression to 60% ONL thickness loss by 18 weeks [422]. *C3ar1^tm1Cge^* mutants show very slow PR degeneration with about 20% loss at 14 months [423]. *Cd46^tm1Atk^* show different rates of PR nuclei loss in male and female mice with 23% and 31% at 12 months of age, respectively [424]. Mutations in *Cx3cr1*, normally expressed in immune cells including microglia, were associated with PR cell loss. Homozygous *Cx3cr1^tm1Litt^* [425] and *Cx3cr1^tm1Zm^* [426] mice on the same C57BL/6J background showed similar rates of PR degeneration with 30% and 40% loss, respectively, at 16–18 months of age. However, *Cx3cr1^tm1Zm^* mice on the BALB/cJ background show complete nuclei loss at 4 months of age [426]. *Cxcr5^tm1Lipp^* causes late onset PR degeneration with 20% loss of ONL thickness at 17 months of age and RPE disorganization [427], whereas ablation of *Irf3* and *Igfbp3* showed mild PR degeneration at 2–4 months of age, about 10–14% [428,429]. *Ccl2* and *Ccr2* mutations also led to PR degeneration and fundus lesions, ONL loss in some areas and development of neovascular lesions, resembling phenotypes of AMD [430]. *Cfh^tm1Mbo^* was shown to have an impairment of rod and cone function by ERG and 29% decreased thickness of Bruch’s membrane; however, rod opsin was distributed normally and no significant reduction in the number of PR cells was observed [431]. *Cfh^tm1.1Son^^g^* demonstrated retinal whitening and cotton wool spots by fundus imaging [432]. Other genes involved in immune function that also showed PR degeneration as conditional knockouts encode transforming growth factor beta receptor II (*Tgfbr2*) [433] and aryl hydrocarbon receptor (*Ahr*) [434].

### 5.3. Omitted Models with PR Abnormalities that May be of Interest

Based on the exclusion criteria described in the Methods section, a number of models with PR abnormalities caused by single gene mutations were not included in our final Table S1. Since we narrowly defined PR degeneration models as post-developmental loss of PR nuclei, some models, which were described with only OS alterations or ERG differences, were not included. For example, mice bearing a spontaneous point mutation in the *Ttc26^hop^* [435] that leads to the generation of a stop codon, Tyr430Ter, were reported to show OS shortening at one year of age with no PR loss. Likewise, ectopic expression of cone opsins in rod OSs led to scotopic ERG abnormalities but not PR degeneration in *Samd7^tmlTFur^* mice at 12 months of age [436]. The many allelic variants that cause ERG abnormalities without PR cell loss are listed in the MGI database and can be accessed through a phenotype query.

### 5.4. Factors Leading to Phenotypic Variability

#### 5.4.1. Effects of Allelic Heterogeneity

Allelic heterogeneity is frequently a cause of phenotypic variability. For mouse models, this is often encountered when comparing a knockout model with spontaneous or induced mutations that still allow a protein to be produced. The latter would primarily be hypomorphic alleles due to amino acid substitutions, some splicing mutations that leave alternate splice forms intact and some C-terminal truncating mutations, which may retain some protein function. Often the knockout allele will be the more severe, presumably because in addition to the loss of protein function, the loss of the protein itself may cause secondary defects such as the failure to form a molecular complex that normally needs the native protein to form.

Mutations in the voltage gated calcium channel, *Cacna1f*, cause congenital stationary night blindness in humans due to abnormal neurotransmitter release in PR synapses. A null mutation in the *Cacna1f* gene (ΔEx14–17) leads to an absent b-wave, abnormal PR synapses, lack of Ca^2+^ response in PR terminals and PR degeneration to 8 rows in the ONL at 8 months [314]. In contrast, an Ile756Thr amino acid substitution found in human patients and introduced into mouse, led to a different phenotype with reduced b-wave, some intact ribbon synapses, a strong abnormal Ca^2+^ response, and a more severe degeneration (3–4 rows at 8 months of age [314]). Here the human allele represents a gain-of-function mutation that in addition to the loss of the original enzyme activity results in a new activity, or causes cell stress, which then induces additional phenotypes and makes the disease presentation more severe.

Within an allelic series of amino acid substitutions there are also frequently gradations of phenotypic severity. If a protein has several functional domains, mutations in different domains may lead to distinct phenotypes. In addition, some mutations can lead to an abnormal tertiary structure of the protein. Such structural changes can lead to a failure to interact with binding partners or substrates/ligands or change the nature of such interactions [437]. Structural changes can also affect export of the protein from the endoplasmic reticulum (ER) and result in ER stress and eventually apoptosis of the cell [438].

One of the larger allelic series available is for human PRPH2 with more than 150 disease causing mutations reported [439]. Although only the secondary structure of the protein is available, some clustering of disease phenotypes is apparent. For example, the area around amino acids 190–220 on the intradiscal loop 2 is enriched for mutations causing autosomal dominant retinitis pigmentosa. This area is thought to interact with ROM1. Mutations leading to macular degeneration are more frequently present between amino acids 142 and 172. However, some macular degeneration and autosomal dominant retinitis pigmentosa mutations are also found elsewhere in the protein [439,440]. Once a 3D structure is available, we may find that the disease specific mutations may well be in spatial proximity and a clearer picture of the genotype-phenotype relation may be revealed.

Allelic heterogeneity can also arise from the intron/exon structure of the gene itself. Many genes produce several distinct transcripts through alternative splicing of their exons [441]. These differing transcripts can each produce proteins, which possess unique functions. For example, the *Rpgrip1* gene produces two splice variants that code for proteins that differ at their C-terminus, a full-length transcript and a shorter transcript encompassing exons 1–13 plus three additional C-terminal amino acids. An insertion between exons 14 and 15 of the full-length transcript leads to PRs with vertically stacked OS discs [442], whereas, a chemically induced mutation in the splice acceptor site in intron 6 that leads to a loss of both splice variant forms results in a failure to develop OSs altogether [443].

Despite the promise of genotype–phenotype correlation analyses to aid in the functional annotation of retinal proteins as well as in the diagnosis and prognosis of retinal degenerative diseases, few allelic series are yet available. In humans the analysis is complicated by the fact that environment and genetic background effects can confound the allelic effect. In animal models, large allelic series are not yet available.

Until recently allelic heterogeneity posed a problem for the generation of mouse models for human retinal diseases because only transgenesis and the generation of knockout models by homologous recombination were available. The removal of the gene products using knockouts can only model recessive or haploinsufficiency diseases, and often the complete lack of the protein will lead to embryonic lethality.

Transgenic models are associated with their own set of problems. Depending on the transgene integration site, the expression of the transgene can be reduced or cellularly restricted. Integration into an unrelated gene can disrupt expression of that gene and cause a phenotype that is not related to the transgene. The use of directed transgene insertion into safe sites, such as the Rosa26 locus (*Gt(ROSA)26Sor*) provides a workaround for some of these problems, although the choice of a promoter that faithfully mimics the native expression is still a difficult process. For these reasons, transgenic mouse models were not included in this review.

With the advent of CRISPR/Cas9 technology to produce precise cuts in genomic DNA, and the ability to perform gene editing through homology directed repair, it is now feasible to recreate human mutations in the mouse and directly probe for the phenotypic effects of allelic heterogeneity [444]. Comitato et al. present an interesting phenotype comparison of transgenic and knock-in rhodopsin P23H models [445].

#### 5.4.2. Effects of Genetic Interactions

Gene interaction, or epistasis, is frequently observed during genetic analysis when two or more alleles at different loci combine to alter the onset, type, or severity of disease phenotypes. Such phenotype altering interactions arise from the organization of proteins and RNAs into macromolecular complexes and/or biochemical and regulatory pathways and networks. For example, consider hypomorphic mutations in two proteins that are components of a linear enzymatic pathway. Individually the reduced activity may not greatly impact the flux through the pathway, but combined in the same cell, the pathway flux may be reduced and become severe enough to induce a disease phenotype due to a lack of sufficient pathway product. Alternatively, a mutation may impair Pathway A, so that a disease phenotype arises. A second mutation may arise in a Pathway B that allows it to compensate for the malfunction in Pathway A and thus reduce the severity of the original disease phenotype. Mutations of this latter type of interacting mutations are called suppressor mutations and are extremely useful because they directly identify potential drug targets whose manipulation may be used to treat disease.

In general, identification of genetic interactors can be useful for placing the primary mutated gene in a biological context and help to define its cellular and organismal function. Often, the known function of a gene and its biology can suggest candidate interacting genes. Similar to the first hypothetical interaction case above, mutations in two proteins involved in iron homeostasis, ceruloplasmin (CP), a ferroxidase associated with transferrin transport across the plasma membrane, and hephaestin (HEPH), implicated in iron transport across cells, individually do not show obvious PR degeneration. Combined in a double mutant mouse model, however, they lead to iron overload in the retina and subsequent RPE abnormalities and PR degeneration [446]. Another example involves two proteins necessary for retinoid recycling, ABCA4 and RDH8. Mutations in each alone do not show any phenotype; combined they cause all-*trans*-retinoid accumulation and PR degeneration [296]. Since previous studies had suggested that activation of TLR3 may lead to inflammation and mediating apoptosis [447], the authors explored the role of *Tlr3* in their *Abca4*/*Rdh8* double mutant model. Importantly, adding a targeted mutation of *Tlr3* to make a triple mutant mouse resulted in rescue of PR cells [448]. Here then the *Tlr3* mutation acts as a suppressor of the degenerative phenotype of the *Abca4*/*Rdh8* double mutant.

Additional interacting gene pairs have been found that affect PR degeneration, among them *Mertk^tmlGrl^*; *Tyro3^tmlGrl^* [449], *Cep290^rd16^*; *Bbs4*^tm1Vcs^ [450], *Cep290^rd16^*; *Mkks*^tm1Vcs^ [451], *Rpgr^tm1Tili^*; *Cep290^rd16^* [452], *Cngb1^tm1.1Biel^*; *Cnga3^tm1Biel^*; *Hcn1^tm2Kndl^* [453], *Crb1^tm1.1Wij^*; *Crb2^tm1.1Wij^* [454], *Dio3^tm1Stg^*; *Dio2^tm1Vag^* [455], and *Ercc6^tm1Gvh^*; and *Xpa^tm1Hvs^* [456].

In addition to testing candidate interacting genes, methods have been developed to identify such interactors in an unbiased fashion that is illustrated below.

Effects of genetic background. For the calcium channel gene *Cacna1f* mentioned above, there is a third allele available. Chang et al. [457] reported the phenotype of the *nob2* mutation, an out-of-frame insertion of a transposable element into the *Cacna1f* gene, which is predicted to cause a truncation after 32 amino acids. The authors demonstrated by western blot that this is a null mutation and no protein is detected. Compared to the ΔEx14–17 null mutation, however, the phenotype of *nob2* is much milder with no apparent PR degradation [457]. The most likely explanation for this discrepancy can be deduced from the fact that the *nob2* mutation arose on the AxB6 recombinant inbred strain, a strain whose DNA is composed of alternate segments derived from C57BL/6J and A/J. It is likely that the A/J strain carries one or more modifier loci that suppress the PR degeneration induced by a *Cacna1f* null mutation.

Upon outcrossing an inbred strain carrying a mutation that leads to a particular phenotype with a different inbred strain, it is frequently observed that the phenotype of the offspring differs from that of the parents. This was often encountered in the past when knockout alleles were created in embryonic stem cells derived from strain 129/Sv and the founder animals were then made congenic on the C57BL/6 background. An early example is a study of a homozygous *Rho* knockout that was shown to lose PR nuclei significantly faster on the 129Sv background than on the C57BL/6 background [458]. Corresponding differences were also found in the number of apoptotic nuclei and in ERG responses. It was concluded that the B6 strain carries protective alleles of modifier genes that lead to a slower rate of PR degeneration [458]. Alternatively, it is also possible that 129Sv carries modifier alleles that accelerate degeneration.

Other inbred strains have also been reported to modify retinal phenotypes. For example, a targeted mutation of *Rp1* (*Rp1^tm1Eap^*) only showed moderate PR degeneration as an incipient congenic (N6) on the A/J strain background, but not on C57BL/6J or DBA/1J backgrounds [459]. ONL dysplasia and excess blue cone formation caused by loss of *Nr2e3* in C57BL/6J are suppressed by the genetic backgrounds of CAST/EiJ, AKR/J, and NOD.NON-*H2^nb1^* strains [460].

In principle, all inbred strains will carry modifier alleles. However, which strain modifies a particular mutation will depend on the primary mutation. It should be emphasized that an inbred strain represents a single genotype. In order to model the phenotypic spectrum of a human disease-causing mutation, many inbred strain backgrounds would have to be examined. Recently, advanced genetically diverse mouse populations have become available, such as the collaborative cross (CC) or the diversity outcross (DO) populations, that allow for more efficient modeling of human populations compared to the classical inbred strains [461,462].

Modifier screens. Modifier screens are a tool to identify genes that modify phenotypic traits caused by a particular mutation. The disease modifying properties of inbred strains have been used for many decades to identify the underlying modifier genes by using genetic crosses, marker assisted genetic mapping of modifying loci, and positional cloning or more recently high throughput whole exome or whole genome sequencing approaches. For example, when B6.Cg-*Nr2e3^rd7^* homozygotes are outcrossed to CAST/EiJ, AKR/J, or NOD.NON-*H2^nb1^* and then the F1 mice intercrossed, homozygous *Nr2e3^rd7^* mice of the F2 generation are found that unlike the parental B6.Cg-*Nr2e3^rd7^* homozygotes have fewer spots on fundus examination and no PR layer dysplasia in histological sections [460]. This phenotypic variability is caused by the genetic interaction between the *Nr2e3^rd7^* disease allele and variants of so-called modifier genes that are specific to the outcross partner strain. Several quantitative trait loci (QTL) on chromosomes 7, 8, 11, and 19 were mapped [460]. Generation of a congenic line carrying the Chr11 modifier, along with further fine mapping, reduced the critical genomic interval to 3.3 cM. Several candidate genes were sequenced and a single nucleotide polymorphism was found in a nuclear receptor gene, *Nr1d1,* that is predicted to lead to an Arg409Gln amino acid change. Causality was confirmed by phenotypic rescue of the *rd7*-associated phenotypes by in vivo electroporation of a wild-type *Nr1d1* expression construct [463].

Several other modifiers have been mapped and identified based on inbred strain differences. For example, mapping crosses have been carried out for *rd3* (BALB/cJ and C57BL/6J, [464]), *rd1* (C3H/HeOu and FVB/N, [465]), *Crb1* (C57BL/6N and C57BL/6JOlaHsd, Chr15, [466]), *Mfrp* (B6.C3Ga and CAST/EiJ, Chr 1, 6, and 11 [467]), and *Tub* and *Tulp1* (C57BL/6J and AKR/J, *Mtap1a*, [468]).

Although not yet widely used as a means to explore retinal biology, a very efficient way to identify modifier genes is the use of a sensitized mutagenesis screen in which a male mouse carrying a mutation of interest is given a chemical mutagen and its offspring are examined for any change in the original phenotype. Offspring carrying a potential mutation is backcrossed to the unmutagenized parental inbred strain to test for heritability and to reduce the mutational load. Mutations are identified using whole exome sequencing of the pheno-deviant mouse. This approach avoids the limited genetic diversity of inbred strains since in principle all genes can be mutated. An example of the utility of mutagenesis to search for modifier genes is the identification of a suppressor mutation in *Frmd4b* that prevents the PR dysplasia and external limiting membrane fragmentation observed in *Nr2e3^rd7^* mutant mice [469].

#### 5.4.3. Effects of Environment on PR Degeneration

PR cell loss has been shown to be induced by a number of environmental factors such as light, diet, and smoking in combination with particular genotypes. Perhaps not surprisingly, light exposure in some models bearing mutations in genes that function directly or in an ancillary fashion in the visual transduction pathway trend toward hastening PR degeneration [470,471]. For example, transgenic mice bearing the rhodopsin VPP mutation, widely used in visual transduction studies, is susceptible to light-exacerbated PR degeneration [472]. Likewise, mice carrying a homozygous *Prom1* null mutation are particularly susceptible to light-induced degeneration. At eye opening, with exposure to light, degeneration initiates at P14, and all PRs are gone by P20, whereas dark rearing from P8 to P30 leads to significant preservation of PRs [471]. Dark-rearing has also been demonstrated to delay PR degeneration in *Slc6a6^tm1Dhau^* (10% loss vs. 90% loss in normal vivarium lighting at three weeks of age) [473] or have no effect in C57BL/6-*Mitf^mi-vit^*/J homozygotes [474]. In some situations, light may actually trigger the disease phenotype, as is the case in *Sag* knockout mice [198,199], with three Class B1 Rhodopsin missense mutations, *Tvrm1* and *Tvrm4* [157] or *Tvrm144* [18], and in null mutation models of *Rdh12* [475], *Asic2* [476], *Myo7a* [477], *Whrn* [478], or *Akt2* [479]. *Sag* mutants must be reared in the dark to observe any PR cells. Under normal vivarium lighting conditions, the other light-sensitive mouse models do not show PR degeneration or only a slight shortening of OS at one year of age, as in the those carrying *Rho* alleles *Tvrm1, Tvrm4,* or *Tvrm144,* and in retinol dehydrogenase (*Rdh12*) mutant mice. However, exposure to bright light or rearing under cyclic moderate-lighting, even subjecting mice to fundus examination, leads to PR degeneration. A comprehensive list of animal models and the effects of dark-rearing or light exposure can be found in reference [470].

Like light exposure, smoking and high fat intake have been proposed to have a negative impact on retinal function by increasing oxidative stress and inflammation in PR and RPE cells [480]. Smoking has been implicated as a major risk factor in the development of age-related macular degeneration in humans [481,482], and the results have been replicated in mouse models as well. Smoking leads to increased oxidative stress and inflammation in B6 mice [483] and in the presence of *Nfe2l2* deficiency [484]. Likewise, combinations of smoking and high fat intake in the presence of an *ApoB* mutation that promotes production of the APOB100 isoform [485] leads to significant loss of PRs [484]. Further, high-fat diet intake for certain genotypes, such as mutations of *Ldlr* [486] or certain alleles of *Apoe* [487], has been shown to compromise PR integrity in mice.

The majority of pharmacological or dietary interventions that have been reported in the relationship to PR degeneration in mouse models are associated with the goal of increasing vitamin A derivative availability [488,489,490] or reducing oxidative stress [491,492] in the retina. Heritable mutations in enzymes, such as LRAT or RPE65, required for processing of vitamin A within the retina are known to cause early onset RD due a deficiency of the 11-*cis*-retinal chromophore. Efficacy of treatment with 9-*cis*-retinal derivatives of mice with null mutations in *Lrat* and *Rpe65* mice is thoroughly discussed in a review by Perusek and Maeda [488,489]. Administration of antioxidants has in some cases improved PR survival. *Rs1^tm1Web^* homozygous females or hemizygous males fed a diet high in DHA [493] or *Pde6b^rd10^* mice fed lutein and zeaxanthin [494] showed a significant PR preservation. Further, injections of a mixture of antioxidants—alpha tocopheral, ascorbic acid, alpha-lipoic acid, and/or Mn(III)tetrakis porphyrin—were able to slow the loss of cone/rod PRs in *Pde6b^rd1^* [495], and *Pde6b^rd10^* mice and in mice with a rhodopsin Q344ter mutation [492]. Environmental enhancement of *Pde6b^rd10^* mice was able to significantly reduce PR loss presumably by reducing retinal oxidative stress [496].

### 5.5. Relationship to Human Disease Genes

**Figure 7:**
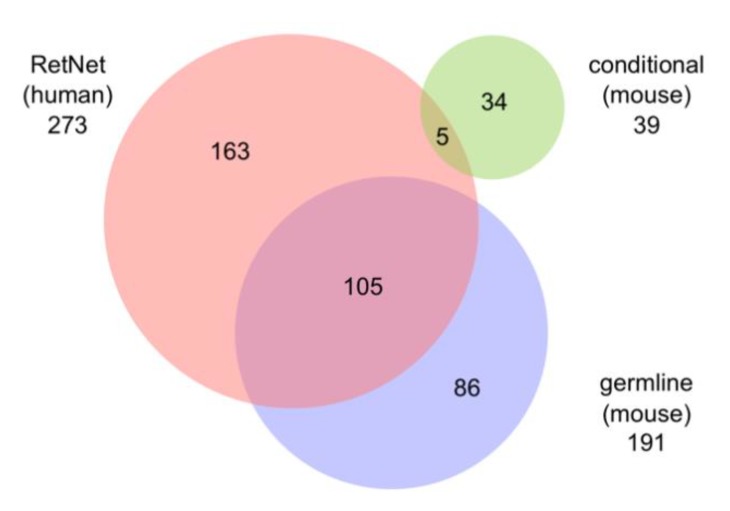
Comparison of the number of RD genes identified in human (RetNet) and mouse as listed in Table S1 and summarized in Figure 3. The total number of genes in the RetNet database that cause monogenic disease and have mouse homologs is indicated, as is the total for which conditional or germline mutations have been associated with PR cell loss in mice, as described in this review. Numbers within the overlapping areas of the diagram represent genes present in both RetNet and Table S1; the remaining numbers represent genes that are unique to the indicated category.

Of the 273 retinal degenerative disease genes in RetNet [3] for which mouse homologs exist, mouse models are available for 110 or 40% of them, including both germline and conditional mutants (Figure 7). Through our survey, we found 120 additional genes, in which mutations lead to PR degeneration. These genes could serve as candidates for yet to be identified human retinal diseases. The available mouse models, for the most part, recapitulate the human disease phenotype well and permit mechanistic and therapeutic studies. However, apparent failures of mouse models do occur. When mutations in *MFRP* were first identified in humans [497], mice were thought to be a poor model because unlike humans [498], mice were previously reported to develop PR degeneration [499], and the microphthalmia and hyperopia found in human patients had not been reported in homozygous *Mfrp^rd6^* mice. In subsequent years, numerous human patients have been identified that do show a degenerative phenotype [500] and hyperopia was detected both in a mouse model carrying a human *MFRP* c.498_499insC allele [501] and the original *Mfrp^rd6^* mouse (our unpublished observations). An important family of deaf–blindness diseases, Usher syndrome, was also thought to be poorly recapitulated in mice, because early models like the shaker-1 mouse had only the characteristic hearing loss, but no retinal degeneration [502]. Later, however, it was found that moderate light exposure does result in photoreceptor degeneration in shaker-1 mice [477]. In addition, a knock-in of the Acadian *USH1C* c.216G>A mutation into the mouse *Ush1c* gene recapitulates both deafness and retinal degeneration phenotypes [503]. In many cases, discordance between the human and mouse phenotypes can be attributed to insufficient information about variation in the human disease, or to allelic effects (knockout vs. hypomorph or gain of function, expression of alternatively splice isoforms), or strain background (modifier genes) in the mouse models. Such shortcomings in mouse models can often be addressed by testing multiple models, including human disease alleles, and by using multiple genetic backgrounds.

Although humans and mice share about 98% of their genes, species differences do exist and need to be considered when selecting a model. Examples of vision-related genes that mice lack are *EYS*, *ARMS2*, and *CETP*. Species differences are the result of different evolutionary histories; humans and mice have encountered different pathogens, resulting in adaptations of our respective immune systems. Mice have different nutritional requirements, resulting in differences in lipid metabolism. Additionally, mouse eyes are adapted to a nocturnal life, resulting in a rod dominated retina with no macula. Nevertheless, mice possess all of the same retinal cell types necessary for vision and the vast majority of the same genes, and even when missing genes are introduced into mice they result in relevant phenotypes. For example, in a transgenic mouse model for Stargardt-like macular degeneration 3 due to a mutation in *Elovl4*, PR cell loss occurs in the central retina in a pattern that resembles the human disease [504]. For the many retinal diseases still in need of models, including complex diseases such as AMD or diabetic retinopathy, it remains the case that valuable new insights into disease mechanism and basic eye biology can still be obtained from mouse studies.

## 6. Discussion

### 6.1. Variability in Measuring PR Cell Loss

We noted an extremely large variability in PR cell loss data presented in the various reports, which were based on several different types of measures, such as ONL thickness obtained from toluidine-stained plastic sections, hematoxylin and eosin-stained paraffin sections, or DAPI-stained cryosections; counts of rows of ONL nuclei in the same preparations; counts of the total number of ONL nuclei in a fixed retinal area; spider plots assessing ONL thickness over the full perimeter; ONL thickness from OCT B-scans. Cone numbers were assessed in sections or whole mounts stained with cone opsins, peanut agglutinin lectin, or cone arrestin. Methods to count cone cell nuclei efficiently might benefit from studies that examine the effect of mutations in genes that specifically affect cones. Perhaps more challenging is that many studies provided insufficient sample sizes or number of time points to assess the progression of PR cell loss. Some of this variability reflects the evolution of methods and quantitative approaches over several decades, and some may be attributed to the different choices each laboratory makes depending on what works best given resources and interests. However, to aid future efforts to compare PR cell loss between studies, we recommend that at least three ages be assessed, one prior to the onset of PR cell loss, and at least two within the age range where PR cells are declining exponentially (that is, between 95% and 5% of wild-type values), and that the data be quantified relative to gender-matched controls at these same ages.

Although a decrease in PR cell numbers as estimated above is widely accepted as evidence for RD, an alternative explanation may apply in some instances. *Mcoln1^tm1Sasl^* homozygotes exhibit an apparent decrease in PR cells to 54% of wild-type at one month of age, a value that remained unchanged at two and six months [505]. TUNEL analysis revealed no increase in apoptosis at any age compared to controls, and both histology and OCT indicated a decrease in total retinal thickness. It is possible that the rapid initial decrease to a stable value may result from retinal thinning as the eye enlarges due to myopia during postnatal development, an occasionally observed feature of *MCOLN1*-associated disease in humans [506]. Methods are available to measure axial length in mice [507], which might be used to determine whether the observed change in nuclear layer thickness is due to ocular enlargement. Models in Table S1 with a similar phenotype include *Guca1a^tm1.1Hunt^* and *Ctnna1^Tvrm5^*.

### 6.2. Correlation of PR Cell Loss with Gene Function

As a fortuitous consequence of our inquiry, in some cases, the progress of PR cell loss could be correlated with gene function within categories. For example, in the ciliary function and trafficking section we see trends in the onset and progression of RD depending on the role and location of gene products within the PR. Mice with mutations in genes involved in ciliogenesis or in transition zone protein complexes, typically result in a PR degeneration that begins at 2 weeks during OS biogenesis and progresses rapidly through OS maturation. Null mutations in genes that encode components of the IFT machinery tend to result in premature death or embryonic lethality, and conditional ablation of these genes in the retina typically leads to early and rapid PR loss. Models with disruptions in protein complexes with roles in BBSome assembly or regulation, protein/lipid trafficking, axoneme extension, or disc morphogenesis tend to have a moderate to slow degeneration. Lastly, mice with mutations that disturb basal body and pericentriolar anchoring and integrity such as the Usher periciliary membrane complex undergo a very slow form of RD, which results in partial PR function throughout the life of the mouse. It remains possible that the slower progression of PR cell loss, especially when associated with members of protein complexes, may be the result of genetic redundancy where multiple genes encode proteins that have similar biochemical functions.

It was also interesting to note that disruption of chloride channels appeared to be particularly deleterious to PR survival. Many of the models we identified with early and rapid progression of PR cell loss, including those affecting a subset of the ciliary genes discussed above or the OS components *Prom1, Prph2,* and *Rho*, appear to be required for OS assembly. Chloride channel defects resulted in similar progression, raising the possibility that chloride homeostasis is important for OS development. This idea is supported by evidence that chloride transport by the chloride channel ANO1 is required for ciliogenesis [508] and that control of intracellular chloride ion levels by this channel regulates the membrane organization of phosphatidylinositol 4,5-bisphosphate [509], a prominent lipid that regulates ciliary development [131,510]. Characterization of early OS development in mouse models defective in chloride channels CLCN2, CLCN3, or CLCN7 may provide mechanistic clues on the role of intracellular chloride in ciliogenesis.

Finally, our analysis revealed links between PR cell loss and a network of 13 genes known to participate in the cellular response to DNA damage, four of which have been directly associated with transcription-coupled DNA repair. Based on canonical indicators of DNA repair, such as the colocalization of phosphorylated histone H2AX and TRP53BP1 (also known as 53BP1) at DSBs, it has been reported that rod PR cells in mice lack a robust canonical DNA damage response, [241,511]. An attenuated response may reflect an adaptation to improve rod cell survival [241,511]. Nevertheless, mechanisms are present in rod cells to repair DNA damage, as evidenced by the robust levels of DNA repair factors [241] and by our results indicating that a network of DNA damage response genes is required for maintaining PR cell viability. Together, these results support the idea that a non-canonical DNA damage response pathway exists in rod PR cells [512]. Further study of the DNA damage response genes linked to PR cell loss in mice may be useful for elucidating this pathway.

## 7. Conclusions

This review highlights mouse models of monogenic retinal degenerative diseases that cause rod or cone PR cell loss. The models include germline mutations and conditional alleles, in which characterization of retinal phenotypes in germline mutations was not possible due to embryonic or perinatal lethality. By providing an extensive list of these models as well as a means of comparing their progression, we hope to benefit researchers who seek to optimize their experimental approaches.

## Supplementary Materials

The following are available online at https://www.mdpi.com/2073-4409/9/4/931/s1, Figure S1: OCT and fundus images of C57BL/6J control mice at various ages, Table S1: Monogenic mouse RD models with PR cell loss, Table S2: Description of gene/protein symbols used in the text and figures.

## References

[1] Cremers F.P.M., Boon C.J.F., Bujakowska K., Zeitz C. (2018). Special Issue Introduction: Inherited Retinal Disease: Novel Candidate Genes, Genotype-Phenotype Correlations, and Inheritance Models. Genes (Basel).

[2] Hanany M., Rivolta C., Sharon D. (2020). Worldwide carrier frequency and genetic prevalence of autosomal recessive inherited retinal diseases. Proc. Natl. Acad. Sci. USA.

[3] RetNet—Retinal Information Network. https://sph.uth.edu/retnet/home.htm.

[4] Picaud S., Dalkara D., Marazova K., Goureau O., Roska B., Sahel J.A. (2019). The primate model for understanding and restoring vision. Proc. Natl. Acad. Sci. USA.

[5] Bunel M., Chaudieu G., Hamel C., Lagoutte L., Manes G., Botherel N., Brabet P., Pilorge P., Andre C., Quignon P. (2019). Natural models for retinitis pigmentosa: Progressive retinal atrophy in dog breeds. Hum. Genet..

[6] Fletcher E.L., Jobling A.I., Vessey K.A., Luu C., Guymer R.H., Baird P.N. (2011). Animal models of retinal disease. Prog. Mol. Biol. Transl. Sci..

[7] Veleri S., Lazar C.H., Chang B., Sieving P.A., Banin E., Swaroop A. (2015). Biology and therapy of inherited retinal degenerative disease: Insights from mouse models. Dis. Model. Mech..

[8] Angueyra J.M., Kindt K.S. (2018). Leveraging Zebrafish to Study Retinal Degenerations. Front. Cell Dev. Biol..

[9] Lehmann M., Knust E., Hebbar S. (2019). Drosophila melanogaster: A Valuable Genetic Model Organism to Elucidate the Biology of Retinitis Pigmentosa. Methods Mol. Biol..

[10] Travis G.H., Brennan M.B., Danielson P.E., Kozak C.A., Sutcliffe J.G. (1989). Identification of a photoreceptor-specific mRNA encoded by the gene responsible for retinal degeneration slow (rds). Nature.

[11] Dryja T.P., McGee T.L., Reichel E., Hahn L.B., Cowley G.S., Yandell D.W., Sandberg M.A., Berson E.L. (1990). A point mutation of the rhodopsin gene in one form of retinitis pigmentosa. Nature.

[12] Bowes C., Li T., Danciger M., Baxter L.C., Applebury M.L., Farber D.B. (1990). Retinal degeneration in the rd mouse is caused by a defect in the beta subunit of rod cGMP-phosphodiesterase. Nature.

[13] Wheway G., Parry D.A., Johnson C.A. (2014). The role of primary cilia in the development and disease of the retina. Organogenesis.

[14] Baehr W., Hanke-Gogokhia C., Sharif A., Reed M., Dahl T., Frederick J.M., Ying G. (2019). Insights into photoreceptor ciliogenesis revealed by animal models. Prog. Retin. Eye Res..

[15] Chang B., Hawes N.L., Hurd R.E., Davisson M.T., Nusinowitz S., Heckenlively J.R. (2002). Retinal degeneration mutants in the mouse. Vis. Res..

[16] Chang B., Hawes N.L., Hurd R.E., Wang J., Howell D., Davisson M.T., Roderick T.H., Nusinowitz S., Heckenlively J.R. (2005). Mouse models of ocular diseases. Vis. NeuroSci..

[17] Won J., Shi L.Y., Hicks W., Wang J., Hurd R., Naggert J.K., Chang B., Nishina P.M. (2011). Mouse model resources for vision research. J. Ophthalmol..

[18] Won J., Shi L.Y., Hicks W., Wang J., Naggert J.K., Nishina P.M. (2012). Translational vision research models program. Adv. Exp. Med. Biol..

[19] Chang B. (2013). Mouse models for studies of retinal degeneration and diseases. Methods Mol. Biol..

[20] Chang B. (2016). Mouse Models as Tools to Identify Genetic Pathways for Retinal Degeneration, as Exemplified by Leber’s Congenital Amaurosis. Methods Mol. Biol..

[21] Krebs M.P., Collin G.B., Hicks W.L., Yu M., Charette J.R., Shi L.Y., Wang J., Naggert J.K., Peachey N.S., Nishina P.M. (2017). Mouse models of human ocular disease for translational research. PLoS ONE.

[22] Do M.T., Yau K.W. (2010). Intrinsically photosensitive retinal ganglion cells. Physiol. Rev..

[23] Goldberg A.F., Moritz O.L., Williams D.S. (2016). Molecular basis for photoreceptor outer segment architecture. Prog. Retin. Eye Res..

[24] Rachel R.A., Li T., Swaroop A. (2012). Photoreceptor sensory cilia and ciliopathies: Focus on CEP290, RPGR and their interacting proteins. Cilia.

[25] Wang J., O’Sullivan M.L., Mukherjee D., Punal V.M., Farsiu S., Kay J.N. (2017). Anatomy and spatial organization of Muller glia in mouse retina. J. Comp. Neurol..

[26] Reichenbach A., Bringmann A. (2019). Glia of the human retina. Glia.

[27] Strauss O. (2005). The retinal pigment epithelium in visual function. Physiol. Rev..

[28] Verbakel S.K., van Huet R.A.C., Boon C.J.F., den Hollander A.I., Collin R.W.J., Klaver C.C.W., Hoyng C.B., Roepman R., Klevering B.J. (2018). Non-syndromic retinitis pigmentosa. Prog. Retin. Eye Res..

[29] Kumaran N., Moore A.T., Weleber R.G., Michaelides M. (2017). Leber congenital amaurosis/early-onset severe retinal dystrophy: Clinical features, molecular genetics and therapeutic interventions. Br. J. Ophthalmol..

[30] Parisi M.A. (2019). The molecular genetics of Joubert syndrome and related ciliopathies: The challenges of genetic and phenotypic heterogeneity. Transl. Sci. Rare Dis..

[31] Suspitsin E.N., Imyanitov E.N. (2016). Bardet-Biedl Syndrome. Mol. Syndromol..

[32] Mathur P., Yang J. (2015). Usher syndrome: Hearing loss, retinal degeneration and associated abnormalities. Biochim. Biophys. Acta.

[33] Kumaran N., Michaelides M., Smith A.J., Ali R.R., Bainbridge J.W.B. (2018). Retinal gene therapy. Br. Med. Bull..

[34] Mouse Genome Database (MGD) at the Mouse Genome Informatics website, The Jackson Laboratory, Bar Harbor, Maine. http://www.informatics.jax.org.

[35] PubMed [Internet] Bethesda (MD): National Library of Medicine (US), National Center for Biotechnology Information. https://www.ncbi.nlm.nih.gov/pubmed/.

[36] Clarke G., Collins R.A., Leavitt B.R., Andrews D.F., Hayden M.R., Lumsden C.J., McInnes R.R. (2000). A one-hit model of cell death in inherited neuronal degenerations. Nature.

[37] Krebs M.P., Xiao M., Sheppard K., Hicks W., Nishina P.M. (2016). Bright-Field Imaging and Optical Coherence Tomography of the Mouse Posterior Eye. Methods Mol. Biol..

[38] Krebs M.P. (2017). Using Vascular Landmarks to Orient 3D Optical Coherence Tomography Images of the Mouse Eye. Curr. Protoc. Mouse Biol..

[39] Dyer M.A., Donovan S.L., Zhang J., Gray J., Ortiz A., Tenney R., Kong J., Allikmets R., Sohocki M.M. (2004). Retinal degeneration in Aipl1-deficient mice: A new genetic model of Leber congenital amaurosis. Brain Res. Mol. Brain Res..

[40] Liu X., Bulgakov O.V., Wen X.H., Woodruff M.L., Pawlyk B., Yang J., Fain G.L., Sandberg M.A., Makino C.L., Li T. (2004). AIPL1, the protein that is defective in Leber congenital amaurosis, is essential for the biosynthesis of retinal rod cGMP phosphodiesterase. Proc. Natl. Acad. Sci. USA.

[41] Dickinson M.E., Flenniken A.M., Ji X., Teboul L., Wong M.D., White J.K., Meehan T.F., Weninger W.J., Westerberg H., Adissu H. (2016). High-throughput discovery of novel developmental phenotypes. Nature.

[42] Hanke-Gogokhia C., Wu Z., Gerstner C.D., Frederick J.M., Zhang H., Baehr W. (2016). Arf-like Protein 3 (ARL3) Regulates Protein Trafficking and Ciliogenesis in Mouse Photoreceptors. J. Biol. Chem..

[43] International Mouse Phenotyping Consortium: Home—IMPC. https://www.mousephenotype.org/.

[44] Bujakowska K.M., Liu Q., Pierce E.A. (2017). Photoreceptor Cilia and Retinal Ciliopathies. Cold Spring Harb. Perspect. Biol..

[45] Gilliam J.C., Chang J.T., Sandoval I.M., Zhang Y., Li T., Pittler S.J., Chiu W., Wensel T.G. (2012). Three-dimensional architecture of the rod sensory cilium and its disruption in retinal neurodegeneration. Cell.

[46] Rohlich P. (1975). The sensory cilium of retinal rods is analogous to the transitional zone of motile cilia. Cell Tiss. Res..

[47] Goncalves J., Pelletier L. (2017). The Ciliary Transition Zone: Finding the Pieces and Assembling the Gate. Mol. Cells.

[48] Seo S., Datta P. (2017). Photoreceptor outer segment as a sink for membrane proteins: Hypothesis and implications in retinal ciliopathies. Hum. Mol. Genet..

[49] Khanna H. (2015). Photoreceptor Sensory Cilium: Traversing the Ciliary Gate. Cells.

[50] Garcia-Gonzalo F.R., Corbit K.C., Sirerol-Piquer M.S., Ramaswami G., Otto E.A., Noriega T.R., Seol A.D., Robinson J.F., Bennett C.L., Josifova D.J. (2011). A transition zone complex regulates mammalian ciliogenesis and ciliary membrane composition. Nat. Genet..

[51] Sedmak T., Wolfrum U. (2011). Intraflagellar transport proteins in ciliogenesis of photoreceptor cells. Biol. Cell.

[52] Salinas R.Y., Pearring J.N., Ding J.D., Spencer W.J., Hao Y., Arshavsky V.Y. (2017). Photoreceptor discs form through peripherin-dependent suppression of ciliary ectosome release. J. Cell Biol..

[53] LaVail M.M. (1973). Kinetics of rod outer segment renewal in the developing mouse retina. J. Cell Biol..

[54] De Robertis E. (1956). Morphogenesis of the retinal rods; an electron microscope study. J. Biophys. Biochem. Cytol..

[55] Insinna C., Besharse J.C. (2008). Intraflagellar transport and the sensory outer segment of vertebrate photoreceptors. Dev. Dyn..

[56] Rosenbaum J.L., Cole D.G., Diener D.R. (1999). Intraflagellar transport: The eyes have it. J. Cell Biol..

[57] Chuang J.Z., Zhao Y., Sung C.H. (2007). SARA-regulated vesicular targeting underlies formation of the light-sensing organelle in mammalian rods. Cell.

[58] Chuang J.Z., Hsu Y.C., Sung C.H. (2015). Ultrastructural visualization of trans-ciliary rhodopsin cargoes in mammalian rods. Cilia.

[59] Steinberg R.H., Fisher S.K., Anderson D.H. (1980). Disc morphogenesis in vertebrate photoreceptors. J. Comp. Neurol..

[60] Ding J.D., Salinas R.Y., Arshavsky V.Y. (2015). Discs of mammalian rod photoreceptors form through the membrane evagination mechanism. J. Cell Biol..

[61] Burgoyne T., Meschede I.P., Burden J.J., Bailly M., Seabra M.C., Futter C.E. (2015). Rod disc renewal occurs by evagination of the ciliary plasma membrane that makes cadherin-based contacts with the inner segment. Proc. Natl. Acad. Sci. USA.

[62] Young R.W. (1967). The renewal of photoreceptor cell outer segments. J. Cell Biol..

[63] Jin H., White S.R., Shida T., Schulz S., Aguiar M., Gygi S.P., Bazan J.F., Nachury M.V. (2010). The conserved Bardet-Biedl syndrome proteins assemble a coat that traffics membrane proteins to cilia. Cell.

[64] Liew G.M., Ye F., Nager A.R., Murphy J.P., Lee J.S., Aguiar M., Breslow D.K., Gygi S.P., Nachury M.V. (2014). The intraflagellar transport protein IFT27 promotes BBSome exit from cilia through the GTPase ARL6/BBS3. Dev. Cell.

[65] Taub D.G., Liu Q. (2016). The Role of Intraflagellar Transport in the Photoreceptor Sensory Cilium. Adv. Exp. Med. Biol..

[66] Pazour G.J., Baker S.A., Deane J.A., Cole D.G., Dickert B.L., Rosenbaum J.L., Witman G.B., Besharse J.C. (2002). The intraflagellar transport protein, IFT88, is essential for vertebrate photoreceptor assembly and maintenance. J. Cell Biol..

[67] Jensen V.L., Leroux M.R. (2017). Gates for soluble and membrane proteins, and two trafficking systems (IFT and LIFT), establish a dynamic ciliary signaling compartment. Curr. Opin. Cell Biol..

[68] Baehr W. (2014). Membrane protein transport in photoreceptors: The function of PDEdelta: The Proctor lecture. Investig. Ophthalmol. Vis. Sci..

[69] Hsu Y., Garrison J.E., Kim G., Schmitz A.R., Searby C.C., Zhang Q., Datta P., Nishimura D.Y., Seo S., Sheffield V.C. (2017). BBSome function is required for both the morphogenesis and maintenance of the photoreceptor outer segment. PLoS Genet..

[70] Jiang L., Wei Y., Ronquillo C.C., Marc R.E., Yoder B.K., Frederick J.M., Baehr W. (2015). Heterotrimeric kinesin-2 (KIF3) mediates transition zone and axoneme formation of mouse photoreceptors. J. Biol. Chem..

[71] Ronquillo C.C., Hanke-Gogokhia C., Revelo M.P., Frederick J.M., Jiang L., Baehr W. (2016). Ciliopathy-associated IQCB1/NPHP5 protein is required for mouse photoreceptor outer segment formation. FASEB J..

[72] Hanke-Gogokhia C., Wu Z., Sharif A., Yazigi H., Frederick J.M., Baehr W. (2017). The guanine nucleotide exchange factor Arf-like protein 13b is essential for assembly of the mouse photoreceptor transition zone and outer segment. J. Biol. Chem..

[73] Cantagrel V., Silhavy J.L., Bielas S.L., Swistun D., Marsh S.E., Bertrand J.Y., Audollent S., Attie-Bitach T., Holden K.R., Dobyns W.B. (2008). Mutations in the cilia gene ARL13B lead to the classical form of Joubert syndrome. Am. J. Hum. Genet..

[74] Caspary T., Larkins C.E., Anderson K.V. (2007). The graded response to Sonic Hedgehog depends on cilia architecture. Dev. Cell.

[75] Eblimit A., Agrawal S.A., Thomas K., Anastassov I.A., Abulikemu T., Moayedi Y., Mardon G., Chen R. (2018). Conditional loss of Spata7 in photoreceptors causes progressive retinal degeneration in mice. Exp. Eye Res..

[76] Singla V., Reiter J.F. (2006). The primary cilium as the cell’s antenna: Signaling at a sensory organelle. Science.

[77] Garcia-Gonzalo F.R., Reiter J.F. (2017). Open Sesame: How Transition Fibers and the Transition Zone Control Ciliary Composition. Cold Spring Harb. Perspect. Biol..

[78] Shi X., Garcia G., Van De Weghe J.C., McGorty R., Pazour G.J., Doherty D., Huang B., Reiter J.F. (2017). Super-resolution microscopy reveals that disruption of ciliary transition-zone architecture causes Joubert syndrome. Nat. Cell Biol..

[79] Williams C.L., Li C., Kida K., Inglis P.N., Mohan S., Semenec L., Bialas N.J., Stupay R.M., Chen N., Blacque O.E. (2011). MKS and NPHP modules cooperate to establish basal body/transition zone membrane associations and ciliary gate function during ciliogenesis. J. Cell Biol..

[80] Knorz V.J., Spalluto C., Lessard M., Purvis T.L., Adigun F.F., Collin G.B., Hanley N.A., Wilson D.I., Hearn T. (2010). Centriolar association of ALMS1 and likely centrosomal functions of the ALMS motif-containing proteins C10orf90 and KIAA1731. Mol. Biol. Cell.

[81] Hearn T., Renforth G.L., Spalluto C., Hanley N.A., Piper K., Brickwood S., White C., Connolly V., Taylor J.F., Russell-Eggitt I. (2002). Mutation of ALMS1, a large gene with a tandem repeat encoding 47 amino acids, causes Alstrom syndrome. Nat. Genet..

[82] Collin G.B., Marshall J.D., Ikeda A., So W.V., Russell-Eggitt I., Maffei P., Beck S., Boerkoel C.F., Sicolo N., Martin M. (2002). Mutations in ALMS1 cause obesity, type 2 diabetes and neurosensory degeneration in Alstrom syndrome. Nat. Genet..

[83] Collin G.B., Cyr E., Bronson R., Marshall J.D., Gifford E.J., Hicks W., Murray S.A., Zheng Q.Y., Smith R.S., Nishina P.M. (2005). Alms1-disrupted mice recapitulate human Alstrom syndrome. Hum. Mol. Genet..

[84] Brun A., Yu X., Obringer C., Ajoy D., Haser E., Stoetzel C., Roux M.J., Messaddeq N., Dollfus H., Marion V. (2019). In vivo phenotypic and molecular characterization of retinal degeneration in mouse models of three ciliopathies. Exp. Eye Res..

[85] May-Simera H.L., Gumerson J.D., Gao C., Campos M., Cologna S.M., Beyer T., Boldt K., Kaya K.D., Patel N., Kretschmer F. (2016). Loss of MACF1 Abolishes Ciliogenesis and Disrupts Apicobasal Polarity Establishment in the Retina. Cell Rep..

[86] Gerding W.M., Schreiber S., Schulte-Middelmann T., de Castro Marques A., Atorf J., Akkad D.A., Dekomien G., Kremers J., Dermietzel R., Gal A. (2011). Ccdc66 null mutation causes retinal degeneration and dysfunction. Hum. Mol. Genet..

[87] Insolera R., Shao W., Airik R., Hildebrandt F., Shi S.H. (2014). SDCCAG8 regulates pericentriolar material recruitment and neuronal migration in the developing cortex. Neuron.

[88] Veleri S., Manjunath S.H., Fariss R.N., May-Simera H., Brooks M., Foskett T.A., Gao C., Longo T.A., Liu P., Nagashima K. (2014). Ciliopathy-associated gene Cc2d2a promotes assembly of subdistal appendages on the mother centriole during cilia biogenesis. Nat. Commun..

[89] Tallila J., Jakkula E., Peltonen L., Salonen R., Kestila M. (2008). Identification of CC2D2A as a Meckel syndrome gene adds an important piece to the ciliopathy puzzle. Am. J. Hum. Genet..

[90] Gorden N.T., Arts H.H., Parisi M.A., Coene K.L., Letteboer S.J., van Beersum S.E., Mans D.A., Hikida A., Eckert M., Knutzen D. (2008). CC2D2A is mutated in Joubert syndrome and interacts with the ciliopathy-associated basal body protein CEP290. Am. J. Hum. Genet..

[91] Mejecase C., Hummel A., Mohand-Said S., Andrieu C., El Shamieh S., Antonio A., Condroyer C., Boyard F., Foussard M., Blanchard S. (2019). Whole exome sequencing resolves complex phenotype and identifies CC2D2A mutations underlying non-syndromic rod-cone dystrophy. Clin. Genet..

[92] Lewis W.R., Bales K.L., Revell D.Z., Croyle M.J., Engle S.E., Song C.J., Malarkey E.B., Uytingco C.R., Shan D., Antonellis P.J. (2019). Mks6 mutations reveal tissue- and cell type-specific roles for the cilia transition zone. FASEB J..

[93] Sorusch N., Bauss K., Plutniok J., Samanta A., Knapp B., Nagel-Wolfrum K., Wolfrum U. (2017). Characterization of the ternary Usher syndrome SANS/ush2a/whirlin protein complex. Hum. Mol. Genet..

[94] Liu X., Bulgakov O.V., Darrow K.N., Pawlyk B., Adamian M., Liberman M.C., Li T. (2007). Usherin is required for maintenance of retinal photoreceptors and normal development of cochlear hair cells. Proc. Natl. Acad. Sci. USA.

[95] Yang J., Liu X., Zhao Y., Adamian M., Pawlyk B., Sun X., McMillan D.R., Liberman M.C., Li T. (2010). Ablation of whirlin long isoform disrupts the USH2 protein complex and causes vision and hearing loss. PLoS Genet..

[96] Khattree N., Ritter L.M., Goldberg A.F. (2013). Membrane curvature generation by a C-terminal amphipathic helix in peripherin-2/rds, a tetraspanin required for photoreceptor sensory cilium morphogenesis. J. Cell Sci..

[97] Molday R.S., Hicks D., Molday L. (1987). Peripherin. A rim-specific membrane protein of rod outer segment discs. Investig. Ophthalmol. Vis. Sci..

[98] Wood C.R., Huang K., Diener D.R., Rosenbaum J.L. (2013). The cilium secretes bioactive ectosomes. Curr. Biol..

[99] Wood C.R., Rosenbaum J.L. (2015). Ciliary ectosomes: Transmissions from the cell’s antenna. Trends Cell Biol..

[100] Stuck M.W., Conley S.M., Naash M.I. (2014). The Y141C knockin mutation in RDS leads to complex phenotypes in the mouse. Hum. Mol. Genet..

[101] Chakraborty D., Conley S.M., Zulliger R., Naash M.I. (2016). The K153Del PRPH2 mutation differentially impacts photoreceptor structure and function. Hum. Mol. Genet..

[102] Sanyal S., De Ruiter A., Hawkins R.K. (1980). Development and degeneration of retina in rds mutant mice: Light microscopy. J. Comp. Neurol..

[103] Sanyal S., Hawkins R.K. (1986). Development and degeneration of retina in rds mutant mice: Effects of light on the rate of degeneration in albino and pigmented homozygous and heterozygous mutant and normal mice. Vis. Res..

[104] McNally N., Kenna P.F., Rancourt D., Ahmed T., Stitt A., Colledge W.H., Lloyd D.G., Palfi A., O’Neill B., Humphries M.M. (2002). Murine model of autosomal dominant retinitis pigmentosa generated by targeted deletion at codon 307 of the rds-peripherin gene. Hum. Mol. Genet..

[105] Chakraborty D., Conley S.M., Al-Ubaidi M.R., Naash M.I. (2014). Initiation of rod outer segment disc formation requires RDS. PLoS ONE.

[106] Kajiwara K., Berson E.L., Dryja T.P. (1994). Digenic retinitis pigmentosa due to mutations at the unlinked peripherin/RDS and ROM1 loci. Science.

[107] Clarke G., Goldberg A.F., Vidgen D., Collins L., Ploder L., Schwarz L., Molday L.L., Rossant J., Szel A., Molday R.S. (2000). Rom-1 is required for rod photoreceptor viability and the regulation of disk morphogenesis. Nat. Genet..

[108] Sato H., Suzuki T., Ikeda K., Masuya H., Sezutsu H., Kaneda H., Kobayashi K., Miura I., Kurihara Y., Yokokura S. (2010). A monogenic dominant mutation in Rom1 generated by N-ethyl-N-nitrosourea mutagenesis causes retinal degeneration in mice. Mol. Vis..

[109] Spencer W.J., Pearring J.N., Salinas R.Y., Loiselle D.R., Skiba N.P., Arshavsky V.Y. (2016). Progressive Rod-Cone Degeneration (PRCD) Protein Requires N-Terminal S-Acylation and Rhodopsin Binding for Photoreceptor Outer Segment Localization and Maintaining Intracellular Stability. Biochemistry.

[110] Allon G., Mann I., Remez L., Sehn E., Rizel L., Nevet M.J., Perlman I., Wolfrum U., Ben-Yosef T. (2019). PRCD is Concentrated at the Base of Photoreceptor Outer Segments and is Involved in Outer Segment Disc Formation. Hum. Mol. Genet..

[111] Zangerl B., Goldstein O., Philp A.R., Lindauer S.J., Pearce-Kelling S.E., Mullins R.F., Graphodatsky A.S., Ripoll D., Felix J.S., Stone E.M. (2006). Identical mutation in a novel retinal gene causes progressive rod-cone degeneration in dogs and retinitis pigmentosa in humans. Genomics.

[112] Spencer W.J., Ding J.D., Lewis T.R., Yu C., Phan S., Pearring J.N., Kim K.Y., Thor A., Mathew R., Kalnitsky J. (2019). PRCD is essential for high-fidelity photoreceptor disc formation. Proc. Natl. Acad. Sci. USA.

[113] Pedersen L.B., Rosenbaum J.L. (2008). Intraflagellar transport (IFT) role in ciliary assembly, resorption and signalling. Curr. Top. Dev. Biol..

[114] Cortellino S., Wang C., Wang B., Bassi M.R., Caretti E., Champeval D., Calmont A., Jarnik M., Burch J., Zaret K.S. (2009). Defective ciliogenesis, embryonic lethality and severe impairment of the Sonic Hedgehog pathway caused by inactivation of the mouse complex A intraflagellar transport gene Ift122/Wdr10, partially overlapping with the DNA repair gene Med1/Mbd4. Dev. Biol..

[115] Murcia N.S., Richards W.G., Yoder B.K., Mucenski M.L., Dunlap J.R., Woychik R.P. (2000). The Oak Ridge Polycystic Kidney (orpk) disease gene is required for left-right axis determination. Development.

[116] Stottmann R.W., Tran P.V., Turbe-Doan A., Beier D.R. (2009). Ttc21b is required to restrict sonic hedgehog activity in the developing mouse forebrain. Dev. Biol..

[117] Gorivodsky M., Mukhopadhyay M., Wilsch-Braeuninger M., Phillips M., Teufel A., Kim C., Malik N., Huttner W., Westphal H. (2009). Intraflagellar transport protein 172 is essential for primary cilia formation and plays a vital role in patterning the mammalian brain. Dev. Biol..

[118] Rix S., Calmont A., Scambler P.J., Beales P.L. (2011). An Ift80 mouse model of short rib polydactyly syndromes shows defects in hedgehog signalling without loss or malformation of cilia. Hum. Mol. Genet..

[119] Berbari N.F., Kin N.W., Sharma N., Michaud E.J., Kesterson R.A., Yoder B.K. (2011). Mutations in Traf3ip1 reveal defects in ciliogenesis, embryonic development, and altered cell size regulation. Dev. Biol..

[120] Bangs F., Anderson K.V. (2017). Primary Cilia and Mammalian Hedgehog Signaling. Cold Spring Harb. Perspect. Biol..

[121] Ko H.W., Liu A., Eggenschwiler J.T. (2009). Analysis of hedgehog signaling in mouse intraflagellar transport mutants. Methods Cell Biol..

[122] Gupta P.R., Pendse N., Greenwald S.H., Leon M., Liu Q., Pierce E.A., Bujakowska K.M. (2018). Ift172 conditional knock-out mice exhibit rapid retinal degeneration and protein trafficking defects. Hum. Mol. Genet..

[123] Keady B.T., Le Y.Z., Pazour G.J. (2011). IFT20 is required for opsin trafficking and photoreceptor outer segment development. Mol. Biol. Cell.

[124] Resh M.D. (2006). Trafficking and signaling by fatty-acylated and prenylated proteins. Nat. Chem. Biol..

[125] Schwarz N., Hardcastle A.J., Cheetham M.E. (2012). Arl3 and RP2 mediated assembly and traffic of membrane associated cilia proteins. Vis. Res..

[126] Li L., Khan N., Hurd T., Ghosh A.K., Cheng C., Molday R., Heckenlively J.R., Swaroop A., Khanna H. (2013). Ablation of the X-linked retinitis pigmentosa 2 (Rp2) gene in mice results in opsin mislocalization and photoreceptor degeneration. Investig. Ophthalmol. Vis. Sci..

[127] Zhang H., Hanke-Gogokhia C., Jiang L., Li X., Wang P., Gerstner C.D., Frederick J.M., Yang Z., Baehr W. (2015). Mistrafficking of prenylated proteins causes retinitis pigmentosa 2. FASEB J..

[128] Wright Z.C., Singh R.K., Alpino R., Goldberg A.F., Sokolov M., Ramamurthy V. (2016). ARL3 regulates trafficking of prenylated phototransduction proteins to the rod outer segment. Hum. Mol. Genet..

[129] Schrick J.J., Vogel P., Abuin A., Hampton B., Rice D.S. (2006). ADP-ribosylation factor-like 3 is involved in kidney and photoreceptor development. Am. J. Pathol..

[130] Rao K.N., Zhang W., Li L., Anand M., Khanna H. (2016). Prenylated retinal ciliopathy protein RPGR interacts with PDE6delta and regulates ciliary localization of Joubert syndrome-associated protein INPP5E. Hum. Mol. Genet..

[131] Xu W., Jin M., Hu R., Wang H., Zhang F., Yuan S., Cao Y. (2017). The Joubert Syndrome Protein Inpp5e Controls Ciliogenesis by Regulating Phosphoinositides at the Apical Membrane. J. Am. Soc. Nephrol..

[132] Gillespie P.G., Prusti R.K., Apel E.D., Beavo J.A. (1989). A soluble form of bovine rod photoreceptor phosphodiesterase has a novel 15-kDa subunit. J. Biol. Chem..

[133] Thompson D.A., Khan N.W., Othman M.I., Chang B., Jia L., Grahek G., Wu Z., Hiriyanna S., Nellissery J., Li T. (2012). Rd9 is a naturally occurring mouse model of a common form of retinitis pigmentosa caused by mutations in RPGR-ORF15. PLoS ONE.

[134] Hong D.H., Pawlyk B.S., Shang J., Sandberg M.A., Berson E.L., Li T. (2000). A retinitis pigmentosa GTPase regulator (RPGR)-deficient mouse model for X-linked retinitis pigmentosa (RP3). Proc. Natl. Acad. Sci. USA.

[135] Zhang H., Li S., Doan T., Rieke F., Detwiler P.B., Frederick J.M., Baehr W. (2007). Deletion of PrBP/delta impedes transport of GRK1 and PDE6 catalytic subunits to photoreceptor outer segments. Proc. Natl. Acad. Sci. USA.

[136] Ramamurthy V., Niemi G.A., Reh T.A., Hurley J.B. (2004). Leber congenital amaurosis linked to AIPL1: A mouse model reveals destabilization of cGMP phosphodiesterase. Proc. Natl. Acad. Sci. USA.

[137] Yadav R.P., Artemyev N.O. (2017). AIPL1: A specialized chaperone for the phototransduction effector. Cell Signal..

[138] Scheidecker S., Etard C., Pierce N.W., Geoffroy V., Schaefer E., Muller J., Chennen K., Flori E., Pelletier V., Poch O. (2014). Exome sequencing of Bardet-Biedl syndrome patient identifies a null mutation in the BBSome subunit BBIP1 (BBS18). J. Med. Genet..

[139] Eichers E.R., Abd-El-Barr M.M., Paylor R., Lewis R.A., Bi W., Lin X., Meehan T.P., Stockton D.W., Wu S.M., Lindsay E. (2006). Phenotypic characterization of Bbs4 null mice reveals age-dependent penetrance and variable expressivity. Hum. Genet..

[140] Mykytyn K., Mullins R.F., Andrews M., Chiang A.P., Swiderski R.E., Yang B., Braun T., Casavant T., Stone E.M., Sheffield V.C. (2004). Bardet-Biedl syndrome type 4 (BBS4)-null mice implicate Bbs4 in flagella formation but not global cilia assembly. Proc. Natl. Acad. Sci. USA.

[141] Koch K.W., Dell’Orco D. (2015). Protein and Signaling Networks in Vertebrate Photoreceptor Cells. Front. Mol. Neurosci..

[142] Pinto L.H., Vitaterna M.H., Shimomura K., Siepka S.M., McDearmon E.L., Fenner D., Lumayag S.L., Omura C., Andrews A.W., Baker M. (2005). Generation, characterization, and molecular cloning of the Noerg-1 mutation of rhodopsin in the mouse. Vis. Neurosci..

[143] Liu H., Wang M., Xia C.H., Du X., Flannery J.G., Ridge K.D., Beutler B., Gong X. (2010). Severe retinal degeneration caused by a novel rhodopsin mutation. Investig. Ophthalmol. Vis. Sci..

[144] Sakami S., Maeda T., Bereta G., Okano K., Golczak M., Sumaroka A., Roman A.J., Cideciyan A.V., Jacobson S.G., Palczewski K. (2011). Probing mechanisms of photoreceptor degeneration in a new mouse model of the common form of autosomal dominant retinitis pigmentosa due to P23H opsin mutations. J. Biol. Chem..

[145] Chiang W.C., Kroeger H., Sakami S., Messah C., Yasumura D., Matthes M.T., Coppinger J.A., Palczewski K., LaVail M.M., Lin J.H. (2015). Robust Endoplasmic Reticulum-Associated Degradation of Rhodopsin Precedes Retinal Degeneration. Mol. Neurobiol..

[146] Comitato A., Schiroli D., Montanari M., Marigo V. (2020). Calpain Activation Is the Major Cause of Cell Death in Photoreceptors Expressing a Rhodopsin Misfolding Mutation. Mol. Neurobiol..

[147] Athanasiou D., Aguila M., Bevilacqua D., Novoselov S.S., Parfitt D.A., Cheetham M.E. (2013). The cell stress machinery and retinal degeneration. FEBS Lett..

[148] Athanasiou D., Aguila M., Bellingham J., Kanuga N., Adamson P., Cheetham M.E. (2017). The role of the ER stress-response protein PERK in rhodopsin retinitis pigmentosa. Hum. Mol. Genet..

[149] Zhang N., Kolesnikov A.V., Jastrzebska B., Mustafi D., Sawada O., Maeda T., Genoud C., Engel A., Kefalov V.J., Palczewski K. (2013). Autosomal recessive retinitis pigmentosa E150K opsin mice exhibit photoreceptor disorganization. J. Clin. Investig..

[150] Sakami S., Kolesnikov A.V., Kefalov V.J., Palczewski K. (2014). P23H opsin knock-in mice reveal a novel step in retinal rod disc morphogenesis. Hum. Mol. Genet..

[151] Hollingsworth T.J., Gross A.K. (2013). The severe autosomal dominant retinitis pigmentosa rhodopsin mutant Ter349Glu mislocalizes and induces rapid rod cell death. J. Biol. Chem..

[152] Humphries M.M., Rancourt D., Farrar G.J., Kenna P., Hazel M., Bush R.A., Sieving P.A., Sheils D.M., McNally N., Creighton P. (1997). Retinopathy induced in mice by targeted disruption of the rhodopsin gene. Nat. Genet..

[153] Lem J., Krasnoperova N.V., Calvert P.D., Kosaras B., Cameron D.A., Nicolo M., Makino C.L., Sidman R.L. (1999). Morphological, physiological, and biochemical changes in rhodopsin knockout mice. Proc. Natl. Acad. Sci. USA.

[154] Faber S., Roepman R. (2019). Balancing the Photoreceptor Proteome: Proteostasis Network Therapeutics for Inherited Retinal Disease. Genes.

[155] Sancho-Pelluz J., Cui X., Lee W., Tsai Y.T., Wu W.H., Justus S., Washington I., Hsu C.W., Park K.S., Koch S. (2019). Mechanisms of neurodegeneration in a preclinical autosomal dominant retinitis pigmentosa knock-in model with a Rho(D190N) mutation. Cell Mol. Life Sci..

[156] Park P.S. (2014). Constitutively active rhodopsin and retinal disease. Adv. Pharmacol..

[157] Budzynski E., Gross A.K., McAlear S.D., Peachey N.S., Shukla M., He F., Edwards M., Won J., Hicks W.L., Wensel T.G. (2010). Mutations of the opsin gene (Y102H and I307N) lead to light-induced degeneration of photoreceptors and constitutive activation of phototransduction in mice. J. Biol. Chem..

[158] Daniele L.L., Insinna C., Chance R., Wang J., Nikonov S.S., Pugh E.N. (2011). A mouse M-opsin monochromat: Retinal cone photoreceptors have increased M-opsin expression when S-opsin is knocked out. Vis. Res..

[159] Zhang Y., Deng W.T., Du W., Zhu P., Li J., Xu F., Sun J., Gerstner C.D., Baehr W., Boye S.L. (2017). Gene-based Therapy in a Mouse Model of Blue Cone Monochromacy. Sci. Rep..

[160] Carter-Dawson L.D., LaVail M.M., Sidman R.L. (1978). Differential effect of the rd mutation on rods and cones in the mouse retina. Investig. Ophthalmol. Vis. Sci..

[161] Calvert P.D., Krasnoperova N.V., Lyubarsky A.L., Isayama T., Nicolo M., Kosaras B., Wong G., Gannon K.S., Margolskee R.F., Sidman R.L. (2000). Phototransduction in transgenic mice after targeted deletion of the rod transducin alpha -subunit. Proc. Natl. Acad. Sci. USA.

[162] Barber A.C., Hippert C., Duran Y., West E.L., Bainbridge J.W., Warre-Cornish K., Luhmann U.F., Lakowski J., Sowden J.C., Ali R.R. (2013). Repair of the degenerate retina by photoreceptor transplantation. Proc. Natl. Acad. Sci. USA.

[163] Miyamoto M., Aoki M., Sugimoto S., Kawasaki K., Imai R. (2010). IRD1 and IRD2 mice, naturally occurring models of hereditary retinal dysfunction, show late-onset and progressive retinal degeneration. Curr. Eye Res..

[164] Mejecase C., Laurent-Coriat C., Mayer C., Poch O., Mohand-Said S., Prevot C., Antonio A., Boyard F., Condroyer C., Michiels C. (2016). Identification of a Novel Homozygous Nonsense Mutation Confirms the Implication of GNAT1 in Rod-Cone Dystrophy. PLoS ONE.

[165] Carrigan M., Duignan E., Humphries P., Palfi A., Kenna P.F., Farrar G.J. (2016). A novel homozygous truncating GNAT1 mutation implicated in retinal degeneration. Br. J. Ophthalmol..

[166] Zenteno J.C., Garcia-Montano L.A., Cruz-Aguilar M., Ronquillo J., Rodas-Serrano A., Aguilar-Castul L., Matsui R., Vencedor-Meraz C.I., Arce-Gonzalez R., Graue-Wiechers F. (2020). Extensive genic and allelic heterogeneity underlying inherited retinal dystrophies in Mexican patients molecularly analyzed by next-generation sequencing. Mol. Genet. Genomic Med..

[167] Deng W.T., Sakurai K., Liu J., Dinculescu A., Li J., Pang J., Min S.H., Chiodo V.A., Boye S.L., Chang B. (2009). Functional interchangeability of rod and cone transducin alpha-subunits. Proc. Natl. Acad. Sci. USA.

[168] Chang B., Hawes N.L., Hurd R.E., Wang J., Davisson M.T., Nusinowitz S., Heckenlively J.R. A New Mouse Model of Retinal Degeneration (rd17). Proceedings of ARVO Annual Meeting Abstract.

[169] Lobanova E.S., Finkelstein S., Herrmann R., Chen Y.M., Kessler C., Michaud N.A., Trieu L.H., Strissel K.J., Burns M.E., Arshavsky V.Y. (2008). Transducin gamma-subunit sets expression levels of alpha- and beta-subunits and is crucial for rod viability. J. Neurosci..

[170] Kolesnikov A.V., Rikimaru L., Hennig A.K., Lukasiewicz P.D., Fliesler S.J., Govardovskii V.I., Kefalov V.J., Kisselev O.G. (2011). G-protein betagamma-complex is crucial for efficient signal amplification in vision. J. Neurosci..

[171] Lobanova E.S., Finkelstein S., Skiba N.P., Arshavsky V.Y. (2013). Proteasome overload is a common stress factor in multiple forms of inherited retinal degeneration. Proc. Natl. Acad. Sci. USA.

[172] Jobling A.I., Vessey K.A., Waugh M., Mills S.A., Fletcher E.L. (2013). A naturally occurring mouse model of achromatopsia: Characterization of the mutation in cone transducin and subsequent retinal phenotype. Investig. Ophthalmol. Vis. Sci..

[173] Chang B., Dacey M.S., Hawes N.L., Hitchcock P.F., Milam A.H., Atmaca-Sonmez P., Nusinowitz S., Heckenlively J.R. (2006). Cone photoreceptor function loss-3, a novel mouse model of achromatopsia due to a mutation in Gnat2. Investig. Ophthalmol. Vis. Sci..

[174] Ronning K.E., Allina G.P., Miller E.B., Zawadzki R.J., Pugh E.N., Herrmann R., Burns M.E. (2018). Loss of cone function without degeneration in a novel Gnat2 knock-out mouse. Exp. Eye Res..

[175] Hirji N., Aboshiha J., Georgiou M., Bainbridge J., Michaelides M. (2018). Achromatopsia: Clinical features, molecular genetics, animal models and therapeutic options. Ophthalmic Genet..

[176] Michaelides M., Aligianis I.A., Holder G.E., Simunovic M., Mollon J.D., Maher E.R., Hunt D.M., Moore A.T. (2003). Cone dystrophy phenotype associated with a frameshift mutation (M280fsX291) in the alpha-subunit of cone specific transducin (GNAT2). Br. J. Ophthalmol..

[177] Du J., An J., Linton J.D., Wang Y., Hurley J.B. (2018). How Excessive cGMP Impacts Metabolic Proteins in Retinas at the Onset of Degeneration. Adv. Exp. Med. Biol..

[178] Tolone A., Belhadj S., Rentsch A., Schwede F., Paquet-Durand F. (2019). The cGMP Pathway and Inherited Photoreceptor Degeneration: Targets, Compounds, and Biomarkers. Genes.

[179] Sothilingam V., Garcia Garrido M., Jiao K., Buena-Atienza E., Sahaboglu A., Trifunovic D., Balendran S., Koepfli T., Muhlfriedel R., Schon C. (2015). Retinitis pigmentosa: Impact of different Pde6a point mutations on the disease phenotype. Hum. Mol. Genet..

[180] Sakamoto K., McCluskey M., Wensel T.G., Naggert J.K., Nishina P.M. (2009). New mouse models for recessive retinitis pigmentosa caused by mutations in the Pde6a gene. Hum. Mol. Genet..

[181] Power M., Das S., Schutze K., Marigo V., Ekstrom P., Paquet-Durand F. (2020). Cellular mechanisms of hereditary photoreceptor degeneration—Focus on cGMP. Prog. Retin. Eye Res..

[182] Tsang S.H., Gouras P., Yamashita C.K., Kjeldbye H., Fisher J., Farber D.B., Goff S.P. (1996). Retinal degeneration in mice lacking the gamma subunit of the rod cGMP phosphodiesterase. Science.

[183] Thiadens A.A., Somervuo V., van den Born L.I., Roosing S., van Schooneveld M.J., Kuijpers R.W., van Moll-Ramirez N., Cremers F.P., Hoyng C.B., Klaver C.C. (2010). Progressive loss of cones in achromatopsia: An imaging study using spectral-domain optical coherence tomography. Investig. Ophthalmol. Vis. Sci..

[184] Brennenstuhl C., Tanimoto N., Burkard M., Wagner R., Bolz S., Trifunovic D., Kabagema-Bilan C., Paquet-Durand F., Beck S.C., Huber G. (2015). Targeted ablation of the Pde6h gene in mice reveals cross-species differences in cone and rod phototransduction protein isoform inventory. J. Biol. Chem..

[185] Kohl S., Coppieters F., Meire F., Schaich S., Roosing S., Brennenstuhl C., Bolz S., van Genderen M.M., Riemslag F.C., European Retinal Disease C. (2012). A nonsense mutation in PDE6H causes autosomal-recessive incomplete achromatopsia. Am. J. Hum. Genet..

[186] Pedurupillay C.R., Landsend E.C., Vigeland M.D., Ansar M., Frengen E., Misceo D., Stromme P. (2016). Segregation of Incomplete Achromatopsia and Alopecia Due to PDE6H and LPAR6 Variants in a Consanguineous Family from Pakistan. Genes.

[187] Weitz D., Ficek N., Kremmer E., Bauer P.J., Kaupp U.B. (2002). Subunit stoichiometry of the CNG channel of rod photoreceptors. Neuron.

[188] Zheng J., Trudeau M.C., Zagotta W.N. (2002). Rod cyclic nucleotide-gated channels have a stoichiometry of three CNGA1 subunits and one CNGB1 subunit. Neuron.

[189] Huttl S., Michalakis S., Seeliger M., Luo D.G., Acar N., Geiger H., Hudl K., Mader R., Haverkamp S., Moser M. (2005). Impaired channel targeting and retinal degeneration in mice lacking the cyclic nucleotide-gated channel subunit CNGB1. J. Neurosci..

[190] Vinberg F., Wang T., Molday R.S., Chen J., Kefalov V.J. (2015). A new mouse model for stationary night blindness with mutant Slc24a1 explains the pathophysiology of the associated human disease. Hum. Mol. Genet..

[191] Yang R.B., Robinson S.W., Xiong W.H., Yau K.W., Birch D.G., Garbers D.L. (1999). Disruption of a retinal guanylyl cyclase gene leads to cone-specific dystrophy and paradoxical rod behavior. J. Neurosci..

[192] Bouzia Z., Georgiou M., Hull S., Robson A.G., Fujinami K., Rotsos T., Pontikos N., Arno G., Webster A.R., Hardcastle A.J. (2020). GUCY2D-Associated Leber Congenital Amaurosis: A Retrospective Natural History Study in Preparation for Trials of Novel Therapies. Am. J. Ophthalmol..

[193] Baehr W., Karan S., Maeda T., Luo D.G., Li S., Bronson J.D., Watt C.B., Yau K.W., Frederick J.M., Palczewski K. (2007). The function of guanylate cyclase 1 and guanylate cyclase 2 in rod and cone photoreceptors. J. Biol. Chem..

[194] Mendez A., Burns M.E., Sokal I., Dizhoor A.M., Baehr W., Palczewski K., Baylor D.A., Chen J. (2001). Role of guanylate cyclase-activating proteins (GCAPs) in setting the flash sensitivity of rod photoreceptors. Proc. Natl. Acad. Sci. USA.

[195] Buch P.K., Mihelec M., Cottrill P., Wilkie S.E., Pearson R.A., Duran Y., West E.L., Michaelides M., Ali R.R., Hunt D.M. (2011). Dominant cone-rod dystrophy: A mouse model generated by gene targeting of the GCAP1/Guca1a gene. PLoS ONE.

[196] Marino V., Dal Cortivo G., Oppici E., Maltese P.E., D’Esposito F., Manara E., Ziccardi L., Falsini B., Magli A., Bertelli M. (2018). A novel p.(Glu111Val) missense mutation in GUCA1A associated with cone-rod dystrophy leads to impaired calcium sensing and perturbed second messenger homeostasis in photoreceptors. Hum. Mol. Genet..

[197] Chen C.K., Burns M.E., Spencer M., Niemi G.A., Chen J., Hurley J.B., Baylor D.A., Simon M.I. (1999). Abnormal photoresponses and light-induced apoptosis in rods lacking rhodopsin kinase. Proc. Natl. Acad. Sci. USA.

[198] Xu J., Dodd R.L., Makino C.L., Simon M.I., Baylor D.A., Chen J. (1997). Prolonged photoresponses in transgenic mouse rods lacking arrestin. Nature.

[199] Chen J., Simon M.I., Matthes M.T., Yasumura D., LaVail M.M. (1999). Increased susceptibility to light damage in an arrestin knockout mouse model of Oguchi disease (stationary night blindness). Investig. Ophthalmol. Vis. Sci..

[200] Charette J.R., Samuels I.S., Yu M., Stone L., Hicks W., Shi L.Y., Krebs M.P., Naggert J.K., Nishina P.M., Peachey N.S. (2016). A Chemical Mutagenesis Screen Identifies Mouse Models with ERG Defects. Adv. Exp. Med. Biol..

[201] Rajappa M., Goyal A., Kaur J. (2010). Inherited metabolic disorders involving the eye: A clinico-biochemical perspective. Eye.

[202] Poll-The B.T., Maillette de Buy Wenniger-Prick C.J. (2011). The eye in metabolic diseases: Clues to diagnosis. Eur. J. Paediatr. Neurol..

[203] Wright A.F., Chakarova C.F., Abd El-Aziz M.M., Bhattacharya S.S. (2010). Photoreceptor degeneration: Genetic and mechanistic dissection of a complex trait. Nat. Rev. Genet..

[204] Fliesler S.J., Anderson R.E. (1983). Chemistry and metabolism of lipids in the vertebrate retina. Prog. Lipid Res..

[205] Giusto N.M., Pasquare S.J., Salvador G.A., Ilincheta de Boschero M.G. (2010). Lipid second messengers and related enzymes in vertebrate rod outer segments. J. Lipid Res..

[206] Niu S.L., Mitchell D.C., Litman B.J. (2002). Manipulation of cholesterol levels in rod disk membranes by methyl-beta-cyclodextrin: Effects on receptor activation. J. Biol. Chem..

[207] Bretillon L., Thuret G., Gregoire S., Acar N., Joffre C., Bron A.M., Gain P., Creuzot-Garcher C.P. (2008). Lipid and fatty acid profile of the retina, retinal pigment epithelium/choroid, and the lacrimal gland, and associations with adipose tissue fatty acids in human subjects. Exp. Eye Res..

[208] Fliesler S.J., Bretillon L. (2010). The ins and outs of cholesterol in the vertebrate retina. J. Lipid Res..

[209] German O.L., Agnolazza D.L., Politi L.E., Rotstein N.P. (2015). Light, lipids and photoreceptor survival: Live or let die?. Photochem. Photobiol. Sci..

[210] Shindou H., Koso H., Sasaki J., Nakanishi H., Sagara H., Nakagawa K.M., Takahashi Y., Hishikawa D., Iizuka-Hishikawa Y., Tokumasu F. (2017). Docosahexaenoic acid preserves visual function by maintaining correct disc morphology in retinal photoreceptor cells. J. Biol. Chem..

[211] Lobanova E.S., Schuhmann K., Finkelstein S., Lewis T.R., Cady M.A., Hao Y., Keuthan C., Ash J.D., Burns M.E., Shevchenko A. (2019). Disrupted Blood-Retina Lysophosphatidylcholine Transport Impairs Photoreceptor Health But Not Visual Signal Transduction. J. Neurosci..

[212] Pham T.L., He J., Kakazu A.H., Jun B., Bazan N.G., Bazan H.E.P. (2017). Defining a mechanistic link between pigment epithelium-derived factor, docosahexaenoic acid, and corneal nerve regeneration. J. Biol. Chem..

[213] Comitato A., Subramanian P., Turchiano G., Montanari M., Becerra S.P., Marigo V. (2018). Pigment epithelium-derived factor hinders photoreceptor cell death by reducing intracellular calcium in the degenerating retina. Cell Death Dis..

[214] Bernstein P.S., Tammur J., Singh N., Hutchinson A., Dixon M., Pappas C.M., Zabriskie N.A., Zhang K., Petrukhin K., Leppert M. (2001). Diverse macular dystrophy phenotype caused by a novel complex mutation in the ELOVL4 gene. Investig. Ophthalmol. Vis. Sci..

[215] Vasireddy V., Jablonski M.M., Mandal M.N., Raz-Prag D., Wang X.F., Nizol L., Iannaccone A., Musch D.C., Bush R.A., Salem N. (2006). Elovl4 5-bp-deletion knock-in mice develop progressive photoreceptor degeneration. Investig. Ophthalmol. Vis. Sci..

[216] Friedman J.S., Chang B., Krauth D.S., Lopez I., Waseem N.H., Hurd R.E., Feathers K.L., Branham K.E., Shaw M., Thomas G.E. (2010). Loss of lysophosphatidylcholine acyltransferase 1 leads to photoreceptor degeneration in rd11 mice. Proc. Natl. Acad. Sci. USA.

[217] Perkovic T., Duh D., Peterlin B., Gregoric J. (2000). The Str mouse as a model for incontinentia pigmenti. Pflugers Arch..

[218] Coleman J.A., Zhu X., Djajadi H.R., Molday L.L., Smith R.S., Libby R.T., John S.W., Molday R.S. (2014). Phospholipid flippase ATP8A2 is required for normal visual and auditory function and photoreceptor and spiral ganglion cell survival. J. Cell Sci..

[219] Bryde S., Hennrich H., Verhulst P.M., Devaux P.F., Lenoir G., Holthuis J.C. (2010). CDC50 proteins are critical components of the human class-1 P4-ATPase transport machinery. J. Biol. Chem..

[220] van der Velden L.M., Wichers C.G., van Breevoort A.E., Coleman J.A., Molday R.S., Berger R., Klomp L.W., van de Graaf S.F. (2010). Heteromeric interactions required for abundance and subcellular localization of human CDC50 proteins and class 1 P4-ATPases. J. Biol. Chem..

[221] Zhang L., Yang Y., Li S., Zhang S., Zhu X., Tai Z., Yang M., Liu Y., Guo X., Chen B. (2017). Loss of Tmem30a leads to photoreceptor degeneration. Sci. Rep..

[222] Wong-Riley M.T. (2010). Energy metabolism of the visual system. Eye Brain.

[223] Du J., Rountree A., Cleghorn W.M., Contreras L., Lindsay K.J., Sadilek M., Gu H., Djukovic D., Raftery D., Satrustegui J. (2016). Phototransduction Influences Metabolic Flux and Nucleotide Metabolism in Mouse Retina. J. Biol. Chem..

[224] Joyal J.S., Sun Y., Gantner M.L., Shao Z., Evans L.P., Saba N., Fredrick T., Burnim S., Kim J.S., Patel G. (2016). Retinal lipid and glucose metabolism dictates angiogenesis through the lipid sensor Ffar1. Nat. Med..

[225] Zhang L., Sun Z., Zhao P., Huang L., Xu M., Yang Y., Chen X., Lu F., Zhang X., Wang H. (2018). Whole-exome sequencing revealed HKDC1 as a candidate gene associated with autosomal-recessive retinitis pigmentosa. Hum. Mol. Genet..

[226] Xia C.H., Lu E., Liu H., Du X., Beutler B., Gong X. (2011). The role of Vldlr in intraretinal angiogenesis in mice. Investig. Ophthalmol. Vis. Sci..

[227] Hu W., Jiang A., Liang J., Meng H., Chang B., Gao H., Qiao X. (2008). Expression of VLDLR in the retina and evolution of subretinal neovascularization in the knockout mouse model’s retinal angiomatous proliferation. Investig. Ophthalmol. Vis. Sci..

[228] Chen Y., Hu Y., Moiseyev G., Zhou K.K., Chen D., Ma J.X. (2009). Photoreceptor degeneration and retinal inflammation induced by very low-density lipoprotein receptor deficiency. Microvasc. Res..

[229] Lin J.B., Kubota S., Ban N., Yoshida M., Santeford A., Sene A., Nakamura R., Zapata N., Kubota M., Tsubota K. (2016). NAMPT-Mediated NAD(+) Biosynthesis Is Essential for Vision In Mice. Cell Rep..

[230] Greenwald S.H., Charette J.R., Staniszewska M., Shi L.Y., Brown S.D.M., Stone L., Liu Q., Hicks W.L., Collin G.B., Bowl M.R. (2016). Mouse Models of NMNAT1-Leber Congenital Amaurosis (LCA9) Recapitulate Key Features of the Human Disease. Am. J. Pathol..

[231] Bosl M.R., Stein V., Hubner C., Zdebik A.A., Jordt S.E., Mukhopadhyay A.K., Davidoff M.S., Holstein A.F., Jentsch T.J. (2001). Male germ cells and photoreceptors, both dependent on close cell-cell interactions, degenerate upon ClC-2 Cl(-) channel disruption. EMBO J..

[232] Ng L., Lyubarsky A., Nikonov S.S., Ma M., Srinivas M., Kefas B., St Germain D.L., Hernandez A., Pugh E.N., Forrest D. (2010). Type 3 deiodinase, a thyroid-hormone-inactivating enzyme, controls survival and maturation of cone photoreceptors. J. Neurosci..

[233] Ng L., Hurley J.B., Dierks B., Srinivas M., Salto C., Vennstrom B., Reh T.A., Forrest D. (2001). A thyroid hormone receptor that is required for the development of green cone photoreceptors. Nat. Genet..

[234] Gianesini C., Hiragaki S., Laurent V., Hicks D., Tosini G. (2016). Cone Viability Is Affected by Disruption of Melatonin Receptors Signaling. Investig. Ophthalmol. Vis. Sci..

[235] Baba K., Pozdeyev N., Mazzoni F., Contreras-Alcantara S., Liu C., Kasamatsu M., Martinez-Merlos T., Strettoi E., Iuvone P.M., Tosini G. (2009). Melatonin modulates visual function and cell viability in the mouse retina via the MT1 melatonin receptor. Proc. Natl. Acad. Sci. USA.

[236] Chen Y., Mehta G., Vasiliou V. (2009). Antioxidant defenses in the ocular surface. Ocul. Surf..

[237] Nita M., Grzybowski A. (2016). The Role of the Reactive Oxygen Species and Oxidative Stress in the Pathomechanism of the Age-Related Ocular Diseases and Other Pathologies of the Anterior and Posterior Eye Segments in Adults. Oxid. Med. Cell Longev..

[238] Chen H., Lukas T.J., Du N., Suyeoka G., Neufeld A.H. (2009). Dysfunction of the retinal pigment epithelium with age: Increased iron decreases phagocytosis and lysosomal activity. Investig. Ophthalmol. Vis. Sci..

[239] Blasiak J., Glowacki S., Kauppinen A., Kaarniranta K. (2013). Mitochondrial and nuclear DNA damage and repair in age-related macular degeneration. Int. J. Mol. Sci..

[240] Tan B.L., Norhaizan M.E., Liew W.P., Sulaiman Rahman H. (2018). Antioxidant and Oxidative Stress: A Mutual Interplay in Age-Related Diseases. Front. Pharmacol..

[241] Frohns A., Frohns F., Naumann S.C., Layer P.G., Lobrich M. (2014). Inefficient double-strand break repair in murine rod photoreceptors with inverted heterochromatin organization. Curr. Biol..

[242] Blasiak J., Petrovski G., Vereb Z., Facsko A., Kaarniranta K. (2014). Oxidative stress, hypoxia, and autophagy in the neovascular processes of age-related macular degeneration. Biomed. Res. Int..

[243] Tokarz P., Kaarniranta K., Blasiak J. (2013). Role of antioxidant enzymes and small molecular weight antioxidants in the pathogenesis of age-related macular degeneration (AMD). Biogerontology.

[244] Hashizume K., Hirasawa M., Imamura Y., Noda S., Shimizu T., Shinoda K., Kurihara T., Noda K., Ozawa Y., Ishida S. (2008). Retinal dysfunction and progressive retinal cell death in SOD1-deficient mice. Am. J. Pathol..

[245] Biswal M.R., Ildefonso C.J., Mao H., Seo S.J., Wang Z., Li H., Le Y.Z., Lewin A.S. (2016). Conditional Induction of Oxidative Stress in RPE: A Mouse Model of Progressive Retinal Degeneration. Adv. Exp. Med. Biol..

[246] Cronin T., Raffelsberger W., Lee-Rivera I., Jaillard C., Niepon M.L., Kinzel B., Clerin E., Petrosian A., Picaud S., Poch O. (2010). The disruption of the rod-derived cone viability gene leads to photoreceptor dysfunction and susceptibility to oxidative stress. Cell Death Differ..

[247] Jaillard C., Mouret A., Niepon M.L., Clerin E., Yang Y., Lee-Rivera I., Ait-Ali N., Millet-Puel G., Cronin T., Sedmak T. (2012). Nxnl2 splicing results in dual functions in neuronal cell survival and maintenance of cell integrity. Hum. Mol. Genet..

[248] Yokota T., Igarashi K., Uchihara T., Jishage K., Tomita H., Inaba A., Li Y., Arita M., Suzuki H., Mizusawa H. (2001). Delayed-onset ataxia in mice lacking alpha -tocopherol transfer protein: Model for neuronal degeneration caused by chronic oxidative stress. Proc. Natl. Acad. Sci. USA.

[249] Mukherjee A.B., Appu A.P., Sadhukhan T., Casey S., Mondal A., Zhang Z., Bagh M.B. (2019). Emerging new roles of the lysosome and neuronal ceroid lipofuscinoses. Mol. Neurodegener..

[250] Schulze H., Kolter T., Sandhoff K. (2009). Principles of lysosomal membrane degradation: Cellular topology and biochemistry of lysosomal lipid degradation. Biochim. Biophys. Acta.

[251] Birch D.G. (1999). Retinal degeneration in retinitis pigmentosa and neuronal ceroid lipofuscinosis: An overview. Mol. Genet. Metab..

[252] Ostergaard J.R. (2016). Juvenile neuronal ceroid lipofuscinosis (Batten disease): Current insights. Degener. Neurol. Neuromuscul. Dis..

[253] Leinonen H., Keksa-Goldsteine V., Ragauskas S., Kohlmann P., Singh Y., Savchenko E., Puranen J., Malm T., Kalesnykas G., Koistinaho J. (2017). Retinal Degeneration In A Mouse Model Of CLN5 Disease Is Associated With Compromised Autophagy. Sci. Rep..

[254] Bartsch U., Galliciotti G., Jofre G.F., Jankowiak W., Hagel C., Braulke T. (2013). Apoptotic photoreceptor loss and altered expression of lysosomal proteins in the nclf mouse model of neuronal ceroid lipofuscinosis. Investig. Ophthalmol. Vis. Sci..

[255] Jankowiak W., Brandenstein L., Dulz S., Hagel C., Storch S., Bartsch U. (2016). Retinal Degeneration in Mice Deficient in the Lysosomal Membrane Protein CLN7. Investig. Ophthalmol. Vis. Sci..

[256] Chang B., Bronson R.T., Hawes N.L., Roderick T.H., Peng C., Hageman G.S., Heckenlively J.R. (1994). Retinal degeneration in motor neuron degeneration: A mouse model of ceroid lipofuscinosis. Investig. Ophthalmol. Vis. Sci..

[257] Hafler B.P., Klein Z.A., Jimmy Zhou Z., Strittmatter S.M. (2014). Progressive retinal degeneration and accumulation of autofluorescent lipopigments in Progranulin deficient mice. Brain Res..

[258] Heldermon C.D., Hennig A.K., Ohlemiller K.K., Ogilvie J.M., Herzog E.D., Breidenbach A., Vogler C., Wozniak D.F., Sands M.S. (2007). Development of sensory, motor and behavioral deficits in the murine model of Sanfilippo syndrome type B. PLoS ONE.

[259] Gelfman C.M., Vogel P., Issa T.M., Turner C.A., Lee W.S., Kornfeld S., Rice D.S. (2007). Mice lacking alpha/beta subunits of GlcNAc-1-phosphotransferase exhibit growth retardation, retinal degeneration, and secretory cell lesions. Investig. Ophthalmol. Vis. Sci..

[260] Kevany B.M., Palczewski K. (2010). Phagocytosis of retinal rod and cone photoreceptors. Physiology.

[261] Prasad D., Rothlin C.V., Burrola P., Burstyn-Cohen T., Lu Q., Garcia de Frutos P., Lemke G. (2006). TAM receptor function in the retinal pigment epithelium. Mol. Cell Neurosci..

[262] Duncan J.L., LaVail M.M., Yasumura D., Matthes M.T., Yang H., Trautmann N., Chappelow A.V., Feng W., Earp H.S., Matsushima G.K. (2003). An RCS-like retinal dystrophy phenotype in mer knockout mice. Investig. Ophthalmol. Vis. Sci..

[263] Houssier M., Raoul W., Lavalette S., Keller N., Guillonneau X., Baragatti B., Jonet L., Jeanny J.C., Behar-Cohen F., Coceani F. (2008). CD36 deficiency leads to choroidal involution via COX2 down-regulation in rodents. PLoS Med..

[264] Ying G., Boldt K., Ueffing M., Gerstner C.D., Frederick J.M., Baehr W. (2018). The small GTPase RAB28 is required for phagocytosis of cone outer segments by the murine retinal pigmented epithelium. J. Biol. Chem..

[265] Lin W., Xu G. (2019). Autophagy: A Role in the Apoptosis, Survival, Inflammation, and Development of the Retina. Ophthalmic Res..

[266] Seranova E., Connolly K.J., Zatyka M., Rosenstock T.R., Barrett T., Tuxworth R.I., Sarkar S. (2017). Dysregulation of autophagy as a common mechanism in lysosomal storage diseases. Essays Biochem..

[267] Byrne S., Jansen L., JM U.K.-I., Siddiqui A., Lidov H.G., Bodi I., Smith L., Mein R., Cullup T., Dionisi-Vici C. (2016). EPG5-related Vici syndrome: A paradigm of neurodevelopmental disorders with defective autophagy. Brain.

[268] Smucker W.D., Kontak J.R. (1990). Adverse drug reactions causing hospital admission in an elderly population: Experience with a decision algorithm. J. Am. Board Fam. Pract..

[269] Kim J.Y., Zhao H., Martinez J., Doggett T.A., Kolesnikov A.V., Tang P.H., Ablonczy Z., Chan C.C., Zhou Z., Green D.R. (2013). Noncanonical autophagy promotes the visual cycle. Cell.

[270] Mohlin C., Taylor L., Ghosh F., Johansson K. (2014). Autophagy and ER-stress contribute to photoreceptor degeneration in cultured adult porcine retina. Brain Res..

[271] Chen Y., Sawada O., Kohno H., Le Y.Z., Subauste C., Maeda T., Maeda A. (2013). Autophagy protects the retina from light-induced degeneration. J. Biol. Chem..

[272] Falk M.J. (2010). Neurodevelopmental manifestations of mitochondrial disease. J. Dev. Behav. Pediatr..

[273] Davies V.J., Powell K.A., White K.E., Yip W., Hogan V., Hollins A.J., Davies J.R., Piechota M., Brownstein D.G., Moat S.J. (2008). A missense mutation in the murine Opa3 gene models human Costeff syndrome. Brain.

[274] Findlay A.S., Carter R.N., Starbuck B., McKie L., Novakova K., Budd P.S., Keighren M.A., Marsh J.A., Cross S.H., Simon M.M. (2018). Mouse Idh3a mutations cause retinal degeneration and reduced mitochondrial function. Dis. Model. Mech..

[275] Bruschi M., Petretto A., Caicci F., Bartolucci M., Calzia D., Santucci L., Manni L., Ramenghi L.A., Ghiggeri G., Traverso C.E. (2018). Proteome of Bovine Mitochondria and Rod Outer Segment Disks: Commonalities and Differences. J. Proteome Res..

[276] Calzia D., Barabino S., Bianchini P., Garbarino G., Oneto M., Caicci F., Diaspro A., Tacchetti C., Manni L., Candiani S. (2013). New findings in ATP supply in rod outer segments: Insights for retinopathies. Biol. Cell.

[277] Funk R.H., Schumann U., Engelmann K., Becker K.A., Roehlecke C. (2014). Blue light induced retinal oxidative stress: Implications for macular degeneration. World J. Ophthalmol..

[278] Calzia D., Garbarino G., Caicci F., Manni L., Candiani S., Ravera S., Morelli A., Traverso C.E., Panfoli I. (2014). Functional expression of electron transport chain complexes in mouse rod outer segments. Biochimie.

[279] Roehlecke C., Schumann U., Ader M., Brunssen C., Bramke S., Morawietz H., Funk R.H. (2013). Stress reaction in outer segments of photoreceptors after blue light irradiation. PLoS ONE.

[280] Wanders R.J., Waterham H.R., Ferdinandusse S. (2015). Metabolic Interplay between Peroxisomes and Other Subcellular Organelles Including Mitochondria and the Endoplasmic Reticulum. Front. Cell Dev. Biol..

[281] Folz S.J., Trobe J.D. (1991). The peroxisome and the eye. Surv. Ophthalmol..

[282] Das Y., Roose N., De Groef L., Fransen M., Moons L., Van Veldhoven P.P., Baes M. (2019). Differential distribution of peroxisomal proteins points to specific roles of peroxisomes in the murine retina. Mol. Cell Biochem..

[283] Hiebler S., Masuda T., Hacia J.G., Moser A.B., Faust P.L., Liu A., Chowdhury N., Huang N., Lauer A., Bennett J. (2014). The Pex1-G844D mouse: A model for mild human Zellweger spectrum disorder. Mol. Genet. Metab..

[284] Pang J.J., Chang B., Hawes N.L., Hurd R.E., Davisson M.T., Li J., Noorwez S.M., Malhotra R., McDowell J.H., Kaushal S. (2005). Retinal degeneration 12 (rd12): A new, spontaneously arising mouse model for human Leber congenital amaurosis (LCA). Mol. Vis..

[285] Wright C.B., Chrenek M.A., Feng W., Getz S.E., Duncan T., Pardue M.T., Feng Y., Redmond T.M., Boatright J.H., Nickerson J.M. (2014). The Rpe65 rd12 allele exerts a semidominant negative effect on vision in mice. Investig. Ophthalmol. Vis. Sci..

[286] Redmond T.M., Yu S., Lee E., Bok D., Hamasaki D., Chen N., Goletz P., Ma J.X., Crouch R.K., Pfeifer K. (1998). Rpe65 is necessary for production of 11-cis-vitamin A in the retinal visual cycle. Nat. Genet..

[287] Tanabu R., Sato K., Monai N., Yamauchi K., Gonome T., Xie Y., Takahashi S., Ishiguro S.I., Nakazawa M. (2019). The findings of optical coherence tomography of retinal degeneration in relation to the morphological and electroretinographic features in RPE65-/- mice. PLoS ONE.

[288] Woodruff M.L., Wang Z., Chung H.Y., Redmond T.M., Fain G.L., Lem J. (2003). Spontaneous activity of opsin apoprotein is a cause of Leber congenital amaurosis. Nat. Genet..

[289] Samardzija M., von Lintig J., Tanimoto N., Oberhauser V., Thiersch M., Reme C.E., Seeliger M., Grimm C., Wenzel A. (2008). R91W mutation in Rpe65 leads to milder early-onset retinal dystrophy due to the generation of low levels of 11-cis-retinal. Hum. Mol. Genet..

[290] Choi E.H., Suh S., Sander C.L., Hernandez C.J.O., Bulman E.R., Khadka N., Dong Z., Shi W., Palczewski K., Kiser P.D. (2018). Insights into the pathogenesis of dominant retinitis pigmentosa associated with a D477G mutation in RPE65. Hum. Mol. Genet..

[291] Radu R.A., Yuan Q., Hu J., Peng J.H., Lloyd M., Nusinowitz S., Bok D., Travis G.H. (2008). Accelerated accumulation of lipofuscin pigments in the RPE of a mouse model for ABCA4-mediated retinal dystrophies following Vitamin A supplementation. Investig. Ophthalmol. Vis. Sci..

[292] Weng J., Mata N.L., Azarian S.M., Tzekov R.T., Birch D.G., Travis G.H. (1999). Insights into the function of Rim protein in photoreceptors and etiology of Stargardt’s disease from the phenotype in abcr knockout mice. Cell.

[293] Molday L.L., Wahl D., Sarunic M.V., Molday R.S. (2018). Localization and functional characterization of the p.Asn965Ser (N965S) ABCA4 variant in mice reveal pathogenic mechanisms underlying Stargardt macular degeneration. Hum. Mol. Genet..

[294] Zhang N., Tsybovsky Y., Kolesnikov A.V., Rozanowska M., Swider M., Schwartz S.B., Stone E.M., Palczewska G., Maeda A., Kefalov V.J. (2015). Protein misfolding and the pathogenesis of ABCA4-associated retinal degenerations. Hum. Mol. Genet..

[295] Wu L., Ueda K., Nagasaki T., Sparrow J.R. (2014). Light damage in Abca4 and Rpe65rd12 mice. Investig. Ophthalmol. Vis. Sci..

[296] Maeda A., Maeda T., Golczak M., Palczewski K. (2008). Retinopathy in mice induced by disrupted all-trans-retinal clearance. J. Biol. Chem..

[297] Chen Y., Okano K., Maeda T., Chauhan V., Golczak M., Maeda A., Palczewski K. (2012). Mechanism of all-trans-retinal toxicity with implications for stargardt disease and age-related macular degeneration. J. Biol. Chem..

[298] Okano K., Maeda A., Chen Y., Chauhan V., Tang J., Palczewska G., Sakai T., Tsuneoka H., Palczewski K., Maeda T. (2012). Retinal cone and rod photoreceptor cells exhibit differential susceptibility to light-induced damage. J. Neurochem..

[299] Batten M.L., Imanishi Y., Maeda T., Tu D.C., Moise A.R., Bronson D., Possin D., Van Gelder R.N., Baehr W., Palczewski K. (2004). Lecithin-retinol acyltransferase is essential for accumulation of all-trans-retinyl esters in the eye and in the liver. J. Biol. Chem..

[300] Ruiz A., Ghyselinck N.B., Mata N., Nusinowitz S., Lloyd M., Dennefeld C., Chambon P., Bok D. (2007). Somatic ablation of the Lrat gene in the mouse retinal pigment epithelium drastically reduces its retinoid storage. Investig. Ophthalmol. Vis. Sci..

[301] Liou G.I., Fei Y., Peachey N.S., Matragoon S., Wei S., Blaner W.S., Wang Y., Liu C., Gottesman M.E., Ripps H. (1998). Early onset photoreceptor abnormalities induced by targeted disruption of the interphotoreceptor retinoid-binding protein gene. J. Neurosci..

[302] Shen J., Shi D., Suzuki T., Xia Z., Zhang H., Araki K., Wakana S., Takeda N., Yamamura K., Jin S. (2016). Severe ocular phenotypes in Rbp4-deficient mice in the C57BL/6 genetic background. Lab. Investig..

[303] Ruiz A., Mark M., Jacobs H., Klopfenstein M., Hu J., Lloyd M., Habib S., Tosha C., Radu R.A., Ghyselinck N.B. (2012). Retinoid content, visual responses, and ocular morphology are compromised in the retinas of mice lacking the retinol-binding protein receptor, STRA6. Investig. Ophthalmol. Vis. Sci..

[304] Amengual J., Zhang N., Kemerer M., Maeda T., Palczewski K., Von Lintig J. (2014). STRA6 is critical for cellular vitamin A uptake and homeostasis. Hum. Mol. Genet..

[305] Wu S.M. (1994). Synaptic transmission in the outer retina. Annu. Rev. Physiol..

[306] Wu S.M. (2010). Synaptic organization of the vertebrate retina: General principles and species-specific variations: The Friedenwald lecture. Investig. Ophthalmol. Vis. Sci..

[307] Mercer A.J., Thoreson W.B. (2011). The dynamic architecture of photoreceptor ribbon synapses: Cytoskeletal, extracellular matrix, and intramembrane proteins. Vis. Neurosci..

[308] Furukawa T., Ueno A., Omori Y. (2019). Molecular mechanisms underlying selective synapse formation of vertebrate retinal photoreceptor cells. Cell Mol. Life Sci..

[309] Pardue M.T., Peachey N.S. (2014). Mouse b-wave mutants. Doc. Ophthalmol..

[310] Pillers D.A., Weleber R.G., Woodward W.R., Green D.G., Chapman V.M., Ray P.N. (1995). mdxCv3 mouse is a model for electroretinography of Duchenne/Becker muscular dystrophy. Investig. Ophthalmol. Vis. Sci..

[311] Satz J.S., Philp A.R., Nguyen H., Kusano H., Lee J., Turk R., Riker M.J., Hernandez J., Weiss R.M., Anderson M.G. (2009). Visual impairment in the absence of dystroglycan. J. Neurosci..

[312] Bytyqi A.H., Lockridge O., Duysen E., Wang Y., Wolfrum U., Layer P.G. (2004). Impaired formation of the inner retina in an AChE knockout mouse results in degeneration of all photoreceptors. Eur. J. Neurosci..

[313] Grisaru D., Sternfeld M., Eldor A., Glick D., Soreq H. (1999). Structural roles of acetylcholinesterase variants in biology and pathology. Eur. J. Biochem..

[314] Regus-Leidig H., Atorf J., Feigenspan A., Kremers J., Maw M.A., Brandstatter J.H. (2014). Photoreceptor degeneration in two mouse models for congenital stationary night blindness type 2. PLoS ONE.

[315] Kerov V., Laird J.G., Joiner M.L., Knecht S., Soh D., Hagen J., Gardner S.H., Gutierrez W., Yoshimatsu T., Bhattarai S. (2018). alpha2delta-4 Is Required for the Molecular and Structural Organization of Rod and Cone Photoreceptor Synapses. J. Neurosci..

[316] Ruether K., Grosse J., Matthiessen E., Hoffmann K., Hartmann C. (2000). Abnormalities of the photoreceptor-bipolar cell synapse in a substrain of C57BL/10 mice. Investig. Ophthalmol. Vis. Sci..

[317] Haeseleer F., Imanishi Y., Maeda T., Possin D.E., Maeda A., Lee A., Rieke F., Palczewski K. (2004). Essential role of Ca2+-binding protein 4, a Cav1.4 channel regulator, in photoreceptor synaptic function. Nat. Neurosci..

[318] Ishiba Y., Higashide T., Mori N., Kobayashi A., Kubota S., McLaren M.J., Satoh H., Wong F., Inana G. (2007). Targeted inactivation of synaptic HRG4 (UNC119) causes dysfunction in the distal photoreceptor and slow retinal degeneration, revealing a new function. Exp. Eye Res..

[319] Haeseleer F. (2008). Interaction and colocalization of CaBP4 and Unc119 (MRG4) in photoreceptors. Investig. Ophthalmol. Vis. Sci..

[320] Giblin J.P., Comes N., Strauss O., Gasull X. (2016). Ion Channels in the Eye: Involvement in Ocular Pathologies. Adv. Protein Chem. Struct. Biol..

[321] Edwards M.M., Marin de Evsikova C., Collin G.B., Gifford E., Wu J., Hicks W.L., Whiting C., Varvel N.H., Maphis N., Lamb B.T. (2010). Photoreceptor degeneration, azoospermia, leukoencephalopathy, and abnormal RPE cell function in mice expressing an early stop mutation in CLCN2. Investig. Ophthalmol. Vis. Sci..

[322] Stobrawa S.M., Breiderhoff T., Takamori S., Engel D., Schweizer M., Zdebik A.A., Bosl M.R., Ruether K., Jahn H., Draguhn A. (2001). Disruption of ClC-3, a chloride channel expressed on synaptic vesicles, leads to a loss of the hippocampus. Neuron.

[323] Rajan I., Read R., Small D.L., Perrard J., Vogel P. (2011). An alternative splicing variant in Clcn7-/- mice prevents osteopetrosis but not neural and retinal degeneration. Vet. Pathol..

[324] Dickerson L.W., Bonthius D.J., Schutte B.C., Yang B., Barna T.J., Bailey M.C., Nehrke K., Williamson R.A., Lamb F.S. (2002). Altered GABAergic function accompanies hippocampal degeneration in mice lacking ClC-3 voltage-gated chloride channels. Brain Res..

[325] Kornak U., Kasper D., Bosl M.R., Kaiser E., Schweizer M., Schulz A., Friedrich W., Delling G., Jentsch T.J. (2001). Loss of the ClC-7 chloride channel leads to osteopetrosis in mice and man. Cell.

[326] Kasper D., Planells-Cases R., Fuhrmann J.C., Scheel O., Zeitz O., Ruether K., Schmitt A., Poet M., Steinfeld R., Schweizer M. (2005). Loss of the chloride channel ClC-7 leads to lysosomal storage disease and neurodegeneration. EMBO J..

[327] Weinert S., Jabs S., Hohensee S., Chan W.L., Kornak U., Jentsch T.J. (2014). Transport activity and presence of ClC-7/Ostm1 complex account for different cellular functions. EMBO Rep..

[328] Weber P., Bartsch U., Schachner M., Montag D. (1998). Na,K-ATPase subunit beta1 knock-in prevents lethality of beta2 deficiency in mice. J. Neurosci..

[329] Heller-Stilb B., van Roeyen C., Rascher K., Hartwig H.G., Huth A., Seeliger M.W., Warskulat U., Haussinger D. (2002). Disruption of the taurine transporter gene (taut) leads to retinal degeneration in mice. FASEB J..

[330] Bok D., Galbraith G., Lopez I., Woodruff M., Nusinowitz S., BeltrandelRio H., Huang W., Zhao S., Geske R., Montgomery C. (2003). Blindness and auditory impairment caused by loss of the sodium bicarbonate cotransporter NBC3. Nat. Genet..

[331] Jin Z.B., Huang X.F., Lv J.N., Xiang L., Li D.Q., Chen J., Huang C., Wu J., Lu F., Qu J. (2014). SLC7A14 linked to autosomal recessive retinitis pigmentosa. Nat. Commun..

[332] Jadeja S., Barnard A.R., McKie L., Cross S.H., White J.K., Sanger Mouse Genetics P., Robertson M., Budd P.S., MacLaren R.E., Jackson I.J. (2015). Mouse slc9a8 mutants exhibit retinal defects due to retinal pigmented epithelium dysfunction. Investig. Ophthalmol. Vis. Sci..

[333] Hori K., Katayama N., Kachi S., Kondo M., Kadomatsu K., Usukura J., Muramatsu T., Mori S., Miyake Y. (2000). Retinal dysfunction in basigin deficiency. Investig. Ophthalmol. Vis. Sci..

[334] Veleri S., Nellissery J., Mishra B., Manjunath S.H., Brooks M.J., Dong L., Nagashima K., Qian H., Gao C., Sergeev Y.V. (2017). REEP6 mediates trafficking of a subset of Clathrin-coated vesicles and is critical for rod photoreceptor function and survival. Hum. Mol. Genet..

[335] Ettaiche M., Deval E., Pagnotta S., Lazdunski M., Lingueglia E. (2009). Acid-sensing ion channel 3 in retinal function and survival. Investig. Ophthalmol. Vis. Sci..

[336] Li L., Jiao X., D’Atri I., Ono F., Nelson R., Chan C.C., Nakaya N., Ma Z., Ma Y., Cai X. (2018). Mutation in the intracellular chloride channel CLCC1 associated with autosomal recessive retinitis pigmentosa. PLoS Genet..

[337] Wong B.H., Chan J.P., Cazenave-Gassiot A., Poh R.W., Foo J.C., Galam D.L., Ghosh S., Nguyen L.N., Barathi V.A., Yeo S.W. (2016). Mfsd2a Is a Transporter for the Essential omega-3 Fatty Acid Docosahexaenoic Acid (DHA) in Eye and Is Important for Photoreceptor Cell Development. J. Biol. Chem..

[338] Mahimkar R.M., Visaya O., Pollock A.S., Lovett D.H. (2005). The disintegrin domain of ADAM9: A ligand for multiple beta1 renal integrins. Biochem. J..

[339] Parry D.A., Toomes C., Bida L., Danciger M., Towns K.V., McKibbin M., Jacobson S.G., Logan C.V., Ali M., Bond J. (2009). Loss of the metalloprotease ADAM9 leads to cone-rod dystrophy in humans and retinal degeneration in mice. Am. J. Hum. Genet..

[340] Vijayasarathy C., Ziccardi L., Sieving P.A. (2012). Biology of retinoschisin. Adv. Exp. Med. Biol..

[341] Jablonski M.M., Dalke C., Wang X., Lu L., Manly K.F., Pretsch W., Favor J., Pardue M.T., Rinchik E.M., Williams R.W. (2005). An ENU-induced mutation in Rs1h causes disruption of retinal structure and function. Mol. Vis..

[342] Han J., Farmer S.R., Kirkland J.L., Corkey B.E., Yoon R., Pirtskhalava T., Ido Y., Guo W. (2002). Octanoate attenuates adipogenesis in 3T3-L1 preadipocytes. J. Nutr..

[343] Zeng Y., Takada Y., Kjellstrom S., Hiriyanna K., Tanikawa A., Wawrousek E., Smaoui N., Caruso R., Bush R.A., Sieving P.A. (2004). RS-1 Gene Delivery to an Adult Rs1h Knockout Mouse Model Restores ERG b-Wave with Reversal of the Electronegative Waveform of X-Linked Retinoschisis. Investig. Ophthalmol. Vis. Sci..

[344] Quinn P.M., Pellissier L.P., Wijnholds J. (2017). The CRB1 Complex: Following the Trail of Crumbs to a Feasible Gene Therapy Strategy. Front. Neurosci..

[345] Mehalow A.K., Kameya S., Smith R.S., Hawes N.L., Denegre J.M., Young J.A., Bechtold L., Haider N.B., Tepass U., Heckenlively J.R. (2003). CRB1 is essential for external limiting membrane integrity and photoreceptor morphogenesis in the mammalian retina. Hum. Mol. Genet..

[346] van de Pavert S.A., Kantardzhieva A., Malysheva A., Meuleman J., Versteeg I., Levelt C., Klooster J., Geiger S., Seeliger M.W., Rashbass P. (2004). Crumbs homologue 1 is required for maintenance of photoreceptor cell polarization and adhesion during light exposure. J. Cell Sci..

[347] Quinn P.M.J., Wijnholds J. (2019). Retinogenesis of the Human Fetal Retina: An Apical Polarity Perspective. Genes.

[348] Moore B.A., Leonard B.C., Sebbag L., Edwards S.G., Cooper A., Imai D.M., Straiton E., Santos L., Reilly C., Griffey S.M. (2018). Identification of genes required for eye development by high-throughput screening of mouse knockouts. Commun. Biol..

[349] Watson J.R., Owen D., Mott H.R. (2017). Cdc42 in actin dynamics: An ordered pathway governed by complex equilibria and directional effector handover. Small GTPases.

[350] Yokokura S., Wada Y., Nakai S., Sato H., Yao R., Yamanaka H., Ito S., Sagara Y., Takahashi M., Nakamura Y. (2005). Targeted disruption of FSCN2 gene induces retinopathy in mice. Investig. Ophthalmol. Vis. Sci..

[351] Liu X., Zhao M., Xie Y., Li P., Wang O., Zhou B., Yang L., Nie Y., Cheng L., Song X. (2018). Null Mutation of the Fascin2 Gene by TALEN Leading to Progressive Hearing Loss and Retinal Degeneration in C57BL/6J Mice. G3.

[352] Saksens N.T., Krebs M.P., Schoenmaker-Koller F.E., Hicks W., Yu M., Shi L., Rowe L., Collin G.B., Charette J.R., Letteboer S.J. (2016). Mutations in CTNNA1 cause butterfly-shaped pigment dystrophy and perturbed retinal pigment epithelium integrity. Nat. Genet..

[353] Maddox D.M., Collin G.B., Ikeda A., Pratt C.H., Ikeda S., Johnson B.A., Hurd R.E., Shopland L.S., Naggert J.K., Chang B. (2015). A Mutation in Syne2 Causes Early Retinal Defects in Photoreceptors, Secondary Neurons, and Muller Glia. Investig. Ophthalmol. Vis. Sci..

[354] Yu J., Lei K., Zhou M., Craft C.M., Xu G., Xu T., Zhuang Y., Xu R., Han M. (2011). KASH protein Syne-2/Nesprin-2 and SUN proteins SUN1/2 mediate nuclear migration during mammalian retinal development. Hum. Mol. Genet..

[355] Gordon M.D., Nusse R. (2006). Wnt signaling: Multiple pathways, multiple receptors, and multiple transcription factors. J. Biol. Chem..

[356] Liu C., Nathans J. (2008). An essential role for frizzled 5 in mammalian ocular development. Development.

[357] Wang Z., Liu C.H., Sun Y., Gong Y., Favazza T.L., Morss P.C., Saba N.J., Fredrick T.W., He X., Akula J.D. (2016). Pharmacologic Activation of Wnt Signaling by Lithium Normalizes Retinal Vasculature in a Murine Model of Familial Exudative Vitreoretinopathy. Am. J. Pathol..

[358] Berger W., van de Pol D., Bachner D., Oerlemans F., Winkens H., Hameister H., Wieringa B., Hendriks W., Ropers H.H. (1996). An animal model for Norrie disease (ND): Gene targeting of the mouse ND gene. Hum. Mol. Genet..

[359] Junge H.J., Yang S., Burton J.B., Paes K., Shu X., French D.M., Costa M., Rice D.S., Ye W. (2009). TSPAN12 regulates retinal vascular development by promoting Norrin- but not Wnt-induced FZD4/beta-catenin signaling. Cell.

[360] Hackam A.S. (2005). The Wnt signaling pathway in retinal degenerations. IUBMB Life.

[361] Hawes N.L., Chang B., Hageman G.S., Nusinowitz S., Nishina P.M., Schneider B.S., Smith R.S., Roderick T.H., Davisson M.T., Heckenlively J.R. (2000). Retinal degeneration 6 (rd6): A new mouse model for human retinitis punctata albescens. Investig. Ophthalmol. Vis. Sci..

[362] Hawkes W.G. (1990). Bibliography: Nurses and smoking. J. N. Y. State Nurses Assoc..

[363] Chavali V.R., Khan N.W., Cukras C.A., Bartsch D.U., Jablonski M.M., Ayyagari R. (2011). A CTRP5 gene S163R mutation knock-in mouse model for late-onset retinal degeneration. Hum. Mol. Genet..

[364] Cai Z., Simons D.L., Fu X.Y., Feng G.S., Wu S.M., Zhang X. (2011). Loss of Shp2-mediated mitogen-activated protein kinase signaling in Muller glial cells results in retinal degeneration. Mol. Cell Biol..

[365] Chavez-Solano M., Ibarra-Sanchez A., Trevino M., Gonzalez-Espinosa C., Lamas M. (2016). Fyn kinase genetic ablation causes structural abnormalities in mature retina and defective Muller cell function. Mol. Cell Neurosci..

[366] Ji X., Liu Y., Hurd R., Wang J., Fitzmaurice B., Nishina P.M., Chang B. (2016). Retinal Pigment Epithelium Atrophy 1 (rpea1): A New Mouse Model With Retinal Detachment Caused by a Disruption of Protein Kinase C, theta. Investig. Ophthalmol. Vis. Sci..

[367] Ivanovic I., Anderson R.E., Le Y.Z., Fliesler S.J., Sherry D.M., Rajala R.V. (2011). Deletion of the p85alpha regulatory subunit of phosphoinositide 3-kinase in cone photoreceptor cells results in cone photoreceptor degeneration. Investig. Ophthalmol. Vis. Sci..

[368] Rajala R.V., Ranjo-Bishop M., Wang Y., Rajala A., Anderson R.E. (2015). The p110alpha isoform of phosphoinositide 3-kinase is essential for cone photoreceptor survival. Biochimie.

[369] Yi X., Schubert M., Peachey N.S., Suzuma K., Burks D.J., Kushner J.A., Suzuma I., Cahill C., Flint C.L., Dow M.A. (2005). Insulin receptor substrate 2 is essential for maturation and survival of photoreceptor cells. J. Neurosci..

[370] Kitahara H., Kajikawa S., Ishii Y., Yamamoto S., Hamashima T., Azuma E., Sato H., Matsushima T., Shibuya M., Shimada Y. (2018). The Novel Pathogenesis of Retinopathy Mediated by Multiple RTK Signals is Uncovered in Newly Developed Mouse Model. EBioMedicine.

[371] Mongan M., Wang J., Liu H., Fan Y., Jin C., Kao W.Y., Xia Y. (2011). Loss of MAP3K1 enhances proliferation and apoptosis during retinal development. Development.

[372] Toyofuku T., Nojima S., Ishikawa T., Takamatsu H., Tsujimura T., Uemura A., Matsuda J., Seki T., Kumanogoh A. (2012). Endosomal sorting by Semaphorin 4A in retinal pigment epithelium supports photoreceptor survival. Genes Dev..

[373] Carter-Dawson L.D., LaVail M.M. (1979). Rods and cones in the mouse retina. II. Autoradiographic analysis of cell generation using tritiated thymidine. J. Comp. Neurol..

[374] Swaroop A., Kim D., Forrest D. (2010). Transcriptional regulation of photoreceptor development and homeostasis in the mammalian retina. Nat. Rev. Neurosci..

[375] Brzezinski J.A., Reh T.A. (2015). Photoreceptor cell fate specification in vertebrates. Development.

[376] Khidr L., Chen P.L. (2006). RB, the conductor that orchestrates life, death and differentiation. Oncogene.

[377] Roger J.E., Hiriyanna A., Gotoh N., Hao H., Cheng D.F., Ratnapriya R., Kautzmann M.A., Chang B., Swaroop A. (2014). OTX2 loss causes rod differentiation defect in CRX-associated congenital blindness. J. Clin. Investig..

[378] Tran N.M., Zhang A., Zhang X., Huecker J.B., Hennig A.K., Chen S. (2014). Mechanistically distinct mouse models for CRX-associated retinopathy. PLoS Genet..

[379] Omori Y., Kitamura T., Yoshida S., Kuwahara R., Chaya T., Irie S., Furukawa T. (2015). Mef2d is essential for the maturation and integrity of retinal photoreceptor and bipolar cells. Genes Cells.

[380] Kiyama T., Chen C.K., Wang S.W., Pan P., Ju Z., Wang J., Takada S., Klein W.H., Mao C.A. (2018). Essential roles of mitochondrial biogenesis regulator Nrf1 in retinal development and homeostasis. Mol. Neurodegener..

[381] Wu F., Li R., Umino Y., Kaczynski T.J., Sapkota D., Li S., Xiang M., Fliesler S.J., Sherry D.M., Gannon M. (2013). Onecut1 is essential for horizontal cell genesis and retinal integrity. J. Neurosci..

[382] Goding C.R., Arnheiter H. (2019). MITF-the first 25 years. Genes Dev..

[383] Zelinger L., Swaroop A. (2018). RNA Biology in Retinal Development and Disease. Trends Genet..

[384] Barabino A., Plamondon V., Abdouh M., Chatoo W., Flamier A., Hanna R., Zhou S., Motoyama N., Hebert M., Lavoie J. (2016). Loss of Bmi1 causes anomalies in retinal development and degeneration of cone photoreceptors. Development.

[385] Susaki K., Kaneko J., Yamano Y., Nakamura K., Inami W., Yoshikawa T., Ozawa Y., Shibata S., Matsuzaki O., Okano H. (2009). Musashi-1, an RNA-binding protein, is indispensable for survival of photoreceptors. Exp. Eye Res..

[386] McKinnon P.J. (2013). Maintaining genome stability in the nervous system. Nat. Neurosci..

[387] Gregersen L.H., Svejstrup J.Q. (2018). The Cellular Response to Transcription-Blocking DNA Damage. Trends Biochem. Sci..

[388] Lans H., Hoeijmakers J.H.J., Vermeulen W., Marteijn J.A. (2019). The DNA damage response to transcription stress. Nat. Rev. Mol. Cell Biol..

[389] Sanchez A., De Vivo A., Uprety N., Kim J., Stevens S.M., Kee Y. (2016). BMI1-UBR5 axis regulates transcriptional repression at damaged chromatin. Proc. Natl. Acad. Sci. USA.

[390] Calses P.C., Dhillon K.K., Tucker N., Chi Y., Huang J.W., Kawasumi M., Nghiem P., Wang Y., Clurman B.E., Jacquemont C. (2017). DGCR8 Mediates Repair of UV-Induced DNA Damage Independently of RNA Processing. Cell Rep..

[391] Burger K., Schlackow M., Potts M., Hester S., Mohammed S., Gullerova M. (2017). Nuclear phosphorylated Dicer processes double-stranded RNA in response to DNA damage. J. Cell Biol..

[392] Montaldo N.P., Bordin D.L., Brambilla A., Rosinger M., Fordyce Martin S.L., Bjoras K.O., Bradamante S., Aas P.A., Furrer A., Olsen L.C. (2019). Alkyladenine DNA glycosylase associates with transcription elongation to coordinate DNA repair with gene expression. Nat. Commun..

[393] Faridounnia M., Folkers G.E., Boelens R. (2018). Function and Interactions of ERCC1-XPF in DNA Damage Response. Molecules.

[394] de Araujo P.R., Gorthi A., da Silva A.E., Tonapi S.S., Vo D.T., Burns S.C., Qiao M., Uren P.J., Yuan Z.M., Bishop A.J. (2016). Musashi1 Impacts Radio-Resistance in Glioblastoma by Controlling DNA-Protein Kinase Catalytic Subunit. Am. J. Pathol..

[395] Onn L., Portillo M., Ilic S., Cleitman G., Stein D., Kaluski S., Shirat I., Slobodnik Z., Einav M., Erdel F. (2020). SIRT6 is a DNA double-strand break sensor. Elife.

[396] Calderwood S.K. (2016). A critical role for topoisomerase IIb and DNA double strand breaks in transcription. Transcription.

[397] Aleksandrov R., Dotchev A., Poser I., Krastev D., Georgiev G., Panova G., Babukov Y., Danovski G., Dyankova T., Hubatsch L. (2018). Protein Dynamics in Complex DNA Lesions. Mol. Cell.

[398] Nishi R., Wijnhoven P.W.G., Kimura Y., Matsui M., Konietzny R., Wu Q., Nakamura K., Blundell T.L., Kessler B.M. (2018). The deubiquitylating enzyme UCHL3 regulates Ku80 retention at sites of DNA damage. Sci. Rep..

[399] Xu H., Lin Z., Li F., Diao W., Dong C., Zhou H., Xie X., Wang Z., Shen Y., Long J. (2015). Dimerization of elongator protein 1 is essential for Elongator complex assembly. Proc. Natl. Acad. Sci. USA.

[400] Li Y., Hao H., Swerdel M.R., Cho H.Y., Lee K.B., Hart R.P., Lyu Y.L., Cai L. (2017). Top2b is involved in the formation of outer segment and synapse during late-stage photoreceptor differentiation by controlling key genes of photoreceptor transcriptional regulatory network. J. Neurosci. Res..

[401] Valdes-Sanchez L., De la Cerda B., Diaz-Corrales F.J., Massalini S., Chakarova C.F., Wright A.F., Bhattacharya S.S. (2013). ATR localizes to the photoreceptor connecting cilium and deficiency leads to severe photoreceptor degeneration in mice. Hum. Mol. Genet..

[402] Bian C., Zhang C., Luo T., Vyas A., Chen S.H., Liu C., Kassab M.A., Yang Y., Kong M., Yu X. (2019). NADP(+) is an endogenous PARP inhibitor in DNA damage response and tumor suppression. Nat. Commun..

[403] Sundermeier T.R., Sakami S., Sahu B., Howell S.J., Gao S., Dong Z., Golczak M., Maeda A., Palczewski K. (2017). MicroRNA-processing Enzymes Are Essential for Survival and Function of Mature Retinal Pigmented Epithelial Cells in Mice. J. Biol. Chem..

[404] Damiani D., Alexander J.J., O’Rourke J.R., McManus M., Jadhav A.P., Cepko C.L., Hauswirth W.W., Harfe B.D., Strettoi E. (2008). Dicer inactivation leads to progressive functional and structural degeneration of the mouse retina. J. Neurosci..

[405] Ueki Y., Ramirez G., Salcedo E., Stabio M.E., Lefcort F. (2016). Loss of Ikbkap Causes Slow, Progressive Retinal Degeneration in a Mouse Model of Familial Dysautonomia. eNeuro.

[406] Spoor M., Nagtegaal A.P., Ridwan Y., Borgesius N.Z., van Alphen B., van der Pluijm I., Hoeijmakers J.H., Frens M.A., Borst J.G. (2012). Accelerated loss of hearing and vision in the DNA-repair deficient Ercc1(delta/-) mouse. Mech. Ageing Dev..

[407] Gorgels T.G., van der Pluijm I., Brandt R.M., Garinis G.A., van Steeg H., van den Aardweg G., Jansen G.H., Ruijter J.M., Bergen A.A., van Norren D. (2007). Retinal degeneration and ionizing radiation hypersensitivity in a mouse model for Cockayne syndrome. Mol. Cell Biol..

[408] Eblimit A., Zaneveld S.A., Liu W., Thomas K., Wang K., Li Y., Mardon G., Chen R. (2018). NMNAT1 E257K variant, associated with Leber Congenital Amaurosis (LCA9), causes a mild retinal degeneration phenotype. Exp. Eye Res..

[409] Peshti V., Obolensky A., Nahum L., Kanfi Y., Rathaus M., Avraham M., Tinman S., Alt F.W., Banin E., Cohen H.Y. (2017). Characterization of physiological defects in adult SIRT6-/- mice. PLoS ONE.

[410] Lim D., Park C.W., Ryu K.Y., Chung H. (2019). Disruption of the polyubiquitin gene Ubb causes retinal degeneration in mice. Biochem. Biophys. Res. Commun..

[411] Semenova E., Wang X., Jablonski M.M., Levorse J., Tilghman S.M. (2003). An engineered 800 kilobase deletion of Uchl3 and Lmo7 on mouse chromosome 14 causes defects in viability, postnatal growth and degeneration of muscle and retina. Hum. Mol. Genet..

[412] Pinelli M., Carissimo A., Cutillo L., Lai C.H., Mutarelli M., Moretti M.N., Singh M.V., Karali M., Carrella D., Pizzo M. (2016). An atlas of gene expression and gene co-regulation in the human retina. Nucleic Acids Res..

[413] Hoshino A., Ratnapriya R., Brooks M.J., Chaitankar V., Wilken M.S., Zhang C., Starostik M.R., Gieser L., La Torre A., Nishio M. (2017). Molecular Anatomy of the Developing Human Retina. Dev. Cell.

[414] Graziotto J.J., Farkas M.H., Bujakowska K., Deramaudt B.M., Zhang Q., Nandrot E.F., Inglehearn C.F., Bhattacharya S.S., Pierce E.A. (2011). Three gene-targeted mouse models of RNA splicing factor RP show late-onset RPE and retinal degeneration. Investig. Ophthalmol. Vis. Sci..

[415] Xu M., Xie Y.A., Abouzeid H., Gordon C.T., Fiorentino A., Sun Z., Lehman A., Osman I.S., Dharmat R., Riveiro-Alvarez R. (2017). Mutations in the Spliceosome Component CWC27 Cause Retinal Degeneration with or without Additional Developmental Anomalies. Am. J. Hum. Genet..

[416] Chen B.J., Lam T.C., Liu L.Q., To C.H. (2017). Post-translational modifications and their applications in eye research (Review). Mol. Med. Rep..

[417] Christiansen J.R., Kolandaivelu S., Bergo M.O., Ramamurthy V. (2011). RAS-converting enzyme 1-mediated endoproteolysis is required for trafficking of rod phosphodiesterase 6 to photoreceptor outer segments. Proc. Natl. Acad. Sci. USA.

[418] Christiansen J.R., Pendse N.D., Kolandaivelu S., Bergo M.O., Young S.G., Ramamurthy V. (2016). Deficiency of Isoprenylcysteine Carboxyl Methyltransferase (ICMT) Leads to Progressive Loss of Photoreceptor Function. J. Neurosci..

[419] Bosch Grau M., Masson C., Gadadhar S., Rocha C., Tort O., Marques Sousa P., Vacher S., Bieche I., Janke C. (2017). Alterations in the balance of tubulin glycylation and glutamylation in photoreceptors leads to retinal degeneration. J. Cell Sci..

[420] Sun X., Park J.H., Gumerson J., Wu Z., Swaroop A., Qian H., Roll-Mecak A., Li T. (2016). Loss of RPGR glutamylation underlies the pathogenic mechanism of retinal dystrophy caused by TTLL5 mutations. Proc. Natl. Acad. Sci. USA.

[421] Blanks J.C., Spee C. (1992). Retinal degeneration in the pcd/pcd mutant mouse: Accumulation of spherules in the interphotoreceptor space. Exp. Eye Res..

[422] Chen J., Qian H., Horai R., Chan C.C., Falick Y., Caspi R.R. (2013). Comparative analysis of induced vs. spontaneous models of autoimmune uveitis targeting the interphotoreceptor retinoid binding protein. PLoS ONE.

[423] Yu M., Zou W., Peachey N.S., McIntyre T.M., Liu J. (2012). A novel role of complement in retinal degeneration. Investig. Ophthalmol. Vis. Sci..

[424] Lyzogubov V.V., Bora P.S., Wu X., Horn L.E., de Roque R., Rudolf X.V., Atkinson J.P., Bora N.S. (2016). The Complement Regulatory Protein CD46 Deficient Mouse Spontaneously Develops Dry-Type Age-Related Macular Degeneration-Like Phenotype. Am. J. Pathol..

[425] Jobling A.I., Waugh M., Vessey K.A., Phipps J.A., Trogrlic L., Greferath U., Mills S.A., Tan Z.L., Ward M.M., Fletcher E.L. (2018). The Role of the Microglial Cx3cr1 Pathway in the Postnatal Maturation of Retinal Photoreceptors. J. Neurosci..

[426] Combadiere C., Feumi C., Raoul W., Keller N., Rodero M., Pezard A., Lavalette S., Houssier M., Jonet L., Picard E. (2007). CX3CR1-dependent subretinal microglia cell accumulation is associated with cardinal features of age-related macular degeneration. J. Clin. Investig..

[427] Huang H., Liu Y., Wang L., Li W. (2017). Age-related macular degeneration phenotypes are associated with increased tumor necrosis-alpha and subretinal immune cells in aged Cxcr5 knockout mice. PLoS ONE.

[428] Zhang X., Zhu J., Chen X., Jie-Qiong Z., Li X., Luo L., Huang H., Liu W., Zhou X., Yan J. (2019). Interferon Regulatory Factor 3 Deficiency Induces Age-Related Alterations of the Retina in Young and Old Mice. Front. Cell Neurosci..

[429] Zhang Q., Jiang Y., Miller M.J., Peng B., Liu L., Soderland C., Tang J., Kern T.S., Pintar J., Steinle J.J. (2013). IGFBP-3 and TNF-alpha regulate retinal endothelial cell apoptosis. Investig. Ophthalmol. Vis. Sci..

[430] Ambati J., Anand A., Fernandez S., Sakurai E., Lynn B.C., Kuziel W.A., Rollins B.J., Ambati B.K. (2003). An animal model of age-related macular degeneration in senescent Ccl-2- or Ccr-2-deficient mice. Nat. Med..

[431] Coffey P.J., Gias C., McDermott C.J., Lundh P., Pickering M.C., Sethi C., Bird A., Fitzke F.W., Maass A., Chen L.L. (2007). Complement factor H deficiency in aged mice causes retinal abnormalities and visual dysfunction. Proc. Natl. Acad. Sci. USA.

[432] Ueda Y., Mohammed I., Song D., Gullipalli D., Zhou L., Sato S., Wang Y., Gupta S., Cheng Z., Wang H. (2017). Murine systemic thrombophilia and hemolytic uremic syndrome from a factor H point mutation. Blood.

[433] Ma W., Silverman S.M., Zhao L., Villasmil R., Campos M.M., Amaral J., Wong W.T. (2019). Absence of TGFbeta signaling in retinal microglia induces retinal degeneration and exacerbates choroidal neovascularization. Elife.

[434] Zhou Y., Li S., Huang L., Yang Y., Zhang L., Yang M., Liu W., Ramasamy K., Jiang Z., Sundaresan P. (2018). A splicing mutation in aryl hydrocarbon receptor associated with retinitis pigmentosa. Hum. Mol. Genet..

[435] Swiderski R.E., Nakano Y., Mullins R.F., Seo S., Banfi B. (2014). A mutation in the mouse ttc26 gene leads to impaired hedgehog signaling. PLoS Genet..

[436] Omori Y., Kubo S., Kon T., Furuhashi M., Narita H., Kominami T., Ueno A., Tsutsumi R., Chaya T., Yamamoto H. (2017). Samd7 is a cell type-specific PRC1 component essential for establishing retinal rod photoreceptor identity. Proc. Natl. Acad. Sci. USA.

[437] Behnen P., Felline A., Comitato A., Di Salvo M.T., Raimondi F., Gulati S., Kahremany S., Palczewski K., Marigo V., Fanelli F. (2018). A Small Chaperone Improves Folding and Routing of Rhodopsin Mutants Linked to Inherited Blindness. iScience.

[438] Kroeger H., Chiang W.C., Felden J., Nguyen A., Lin J.H. (2019). ER stress and unfolded protein response in ocular health and disease. FEBS J..

[439] Stuck M.W., Conley S.M., Naash M.I. (2016). PRPH2/RDS and ROM-1: Historical context, current views and future considerations. Prog. Retin. Eye Res..

[440] Boon C.J., den Hollander A.I., Hoyng C.B., Cremers F.P., Klevering B.J., Keunen J.E. (2008). The spectrum of retinal dystrophies caused by mutations in the peripherin/RDS gene. Prog. Retin. Eye Res..

[441] Liu M.M., Zack D.J. (2013). Alternative splicing and retinal degeneration. Clin. Genet..

[442] Zhao Y., Hong D.H., Pawlyk B., Yue G., Adamian M., Grynberg M., Godzik A., Li T. (2003). The retinitis pigmentosa GTPase regulator (RPGR)- interacting protein: Subserving RPGR function and participating in disk morphogenesis. Proc. Natl. Acad. Sci. USA.

[443] Won J., Gifford E., Smith R.S., Yi H., Ferreira P.A., Hicks W.L., Li T., Naggert J.K., Nishina P.M. (2009). RPGRIP1 is essential for normal rod photoreceptor outer segment elaboration and morphogenesis. Hum. Mol. Genet..

[444] Zhang C., Quan R., Wang J. (2018). Development and application of CRISPR/Cas9 technologies in genomic editing. Hum. Mol. Genet..

[445] Comitato A., Schiroli D., La Marca C., Marigo V. (2019). Differential Contribution of Calcium-Activated Proteases and ER-Stress in Three Mouse Models of Retinitis Pigmentosa Expressing P23H Mutant RHO. Adv. Exp. Med. Biol..

[446] Hahn P., Qian Y., Dentchev T., Chen L., Beard J., Harris Z.L., Dunaief J.L. (2004). Disruption of ceruloplasmin and hephaestin in mice causes retinal iron overload and retinal degeneration with features of age-related macular degeneration. Proc. Natl. Acad. Sci. USA.

[447] Kumar M.V., Nagineni C.N., Chin M.S., Hooks J.J., Detrick B. (2004). Innate immunity in the retina: Toll-like receptor (TLR) signaling in human retinal pigment epithelial cells. J. Neuroimmunol..

[448] Shiose S., Chen Y., Okano K., Roy S., Kohno H., Tang J., Pearlman E., Maeda T., Palczewski K., Maeda A. (2011). Toll-like receptor 3 is required for development of retinopathy caused by impaired all-trans-retinal clearance in mice. J. Biol. Chem..

[449] Vollrath D., Yasumura D., Benchorin G., Matthes M.T., Feng W., Nguyen N.M., Sedano C.D., Calton M.A., LaVail M.M. (2015). Tyro3 Modulates Mertk-Associated Retinal Degeneration. PLoS Genet..

[450] Zhang Y., Seo S., Bhattarai S., Bugge K., Searby C.C., Zhang Q., Drack A.V., Stone E.M., Sheffield V.C. (2014). BBS mutations modify phenotypic expression of CEP290-related ciliopathies. Hum. Mol. Genet..

[451] Rachel R.A., May-Simera H.L., Veleri S., Gotoh N., Choi B.Y., Murga-Zamalloa C., McIntyre J.C., Marek J., Lopez I., Hackett A.N. (2012). Combining Cep290 and Mkks ciliopathy alleles in mice rescues sensory defects and restores ciliogenesis. J. Clin. Investig..

[452] Rao K.N., Zhang W., Li L., Ronquillo C., Baehr W., Khanna H. (2016). Ciliopathy-associated protein CEP290 modifies the severity of retinal degeneration due to loss of RPGR. Hum. Mol. Genet..

[453] Schon C., Asteriti S., Koch S., Sothilingam V., Garcia Garrido M., Tanimoto N., Herms J., Seeliger M.W., Cangiano L., Biel M. (2016). Loss of HCN1 enhances disease progression in mouse models of CNG channel-linked retinitis pigmentosa and achromatopsia. Hum. Mol. Genet..

[454] Quinn P.M., Mulder A.A., Henrique Alves C., Desrosiers M., de Vries S.I., Klooster J., Dalkara D., Koster A.J., Jost C.R., Wijnholds J. (2019). Loss of CRB2 in Muller glial cells modifies a CRB1-associated retinitis pigmentosa phenotype into a Leber congenital amaurosis phenotype. Hum. Mol. Genet..

[455] Ng L., Liu H., St Germain D.L., Hernandez A., Forrest D. (2017). Deletion of the Thyroid Hormone-Activating Type 2 Deiodinase Rescues Cone Photoreceptor Degeneration but Not Deafness in Mice Lacking Type 3 Deiodinase. Endocrinology.

[456] van der Pluijm I., Garinis G.A., Brandt R.M., Gorgels T.G., Wijnhoven S.W., Diderich K.E., de Wit J., Mitchell J.R., van Oostrom C., Beems R. (2007). Impaired genome maintenance suppresses the growth hormone--insulin-like growth factor 1 axis in mice with Cockayne syndrome. PLoS Biol..

[457] Chang B., Heckenlively J.R., Bayley P.R., Brecha N.C., Davisson M.T., Hawes N.L., Hirano A.A., Hurd R.E., Ikeda A., Johnson B.A. (2006). The nob2 mouse, a null mutation in Cacna1f: Anatomical and functional abnormalities in the outer retina and their consequences on ganglion cell visual responses. Vis. Neurosci..

[458] Humphries M.M., Kiang S., McNally N., Donovan M.A., Sieving P.A., Bush R.A., Machida S., Cotter T., Hobson A., Farrar J. (2001). Comparative structural and functional analysis of photoreceptor neurons of Rho-/- mice reveal increased survival on C57BL/6J in comparison to 129Sv genetic background. Vis. Neurosci..

[459] Liu Q., Saveliev A., Pierce E.A. (2009). The severity of retinal degeneration in Rp1h gene-targeted mice is dependent on genetic background. Investig. Ophthalmol. Vis. Sci..

[460] Haider N.B., Zhang W., Hurd R., Ikeda A., Nystuen A.M., Naggert J.K., Nishina P.M. (2008). Mapping of genetic modifiers of Nr2e3 rd7/rd7 that suppress retinal degeneration and restore blue cone cells to normal quantity. Mamm. Genome.

[461] Threadgill D.W., Miller D.R., Churchill G.A., de Villena F.P. (2011). The collaborative cross: A recombinant inbred mouse population for the systems genetic era. ILAR J..

[462] Churchill G.A., Gatti D.M., Munger S.C., Svenson K.L. (2012). The Diversity Outbred mouse population. Mamm. Genome.

[463] Cruz N.M., Yuan Y., Leehy B.D., Baid R., Kompella U., DeAngelis M.M., Escher P., Haider N.B. (2014). Modifier genes as therapeutics: The nuclear hormone receptor Rev Erb alpha (Nr1d1) rescues Nr2e3 associated retinal disease. PLoS ONE.

[464] Danciger M., Ogando D., Yang H., Matthes M.T., Yu N., Ahern K., Yasumura D., Williams R.W., Lavail M.M. (2008). Genetic modifiers of retinal degeneration in the rd3 mouse. Investig. Ophthalmol. Vis. Sci..

[465] van Wyk M., Schneider S., Kleinlogel S. (2015). Variable phenotypic expressivity in inbred retinal degeneration mouse lines: A comparative study of C3H/HeOu and FVB/N rd1 mice. Mol. Vis..

[466] Luhmann U.F., Carvalho L.S., Holthaus S.M., Cowing J.A., Greenaway S., Chu C.J., Herrmann P., Smith A.J., Munro P.M., Potter P. (2015). The severity of retinal pathology in homozygous Crb1rd8/rd8 mice is dependent on additional genetic factors. Hum. Mol. Genet..

[467] Won J., Charette J.R., Philip V.M., Stearns T.M., Zhang W., Naggert J.K., Krebs M.P., Nishina P.M. (2014). Genetic modifier loci of mouse Mfrp(rd6) identified by quantitative trait locus analysis. Exp. Eye Res..

[468] Maddox D.M., Ikeda S., Ikeda A., Zhang W., Krebs M.P., Nishina P.M., Naggert J.K. (2012). An allele of microtubule-associated protein 1A (Mtap1a) reduces photoreceptor degeneration in Tulp1 and Tub Mutant Mice. Investig. Ophthalmol. Vis. Sci..

[469] Kong Y., Zhao L., Charette J.R., Hicks W.L., Stone L., Nishina P.M., Naggert J.K. (2018). An FRMD4B variant suppresses dysplastic photoreceptor lesions in models of enhanced S-cone syndrome and of Nrl deficiency. Hum. Mol. Genet..

[470] Paskowitz D.M., LaVail M.M., Duncan J.L. (2006). Light and inherited retinal degeneration. Br. J. Ophthalmol..

[471] Dellett M., Sasai N., Nishide K., Becker S., Papadaki V., Limb G.A., Moore A.T., Kondo T., Ohnuma S. (2014). Genetic background and light-dependent progression of photoreceptor cell degeneration in Prominin-1 knockout mice. Investig. Ophthalmol. Vis. Sci..

[472] Naash M.L., Peachey N.S., Li Z.Y., Gryczan C.C., Goto Y., Blanks J., Milam A.H., Ripps H. (1996). Light-induced acceleration of photoreceptor degeneration in transgenic mice expressing mutant rhodopsin. Investig. Ophthalmol. Vis. Sci..

[473] Rascher K., Servos G., Berthold G., Hartwig H.G., Warskulat U., Heller-Stilb B., Haussinger D. (2004). Light deprivation slows but does not prevent the loss of photoreceptors in taurine transporter knockout mice. Vis. Res..

[474] Smith S.B., Cope B.K., McCoy J.R. (1994). Effects of dark-rearing on the retinal degeneration of the C57BL/6-mivit/mivit mouse. Exp. Eye Res..

[475] Maeda A., Maeda T., Imanishi Y., Sun W., Jastrzebska B., Hatala D.A., Winkens H.J., Hofmann K.P., Janssen J.J., Baehr W. (2006). Retinol dehydrogenase (RDH12) protects photoreceptors from light-induced degeneration in mice. J. Biol. Chem..

[476] Ettaiche M., Guy N., Hofman P., Lazdunski M., Waldmann R. (2004). Acid-sensing ion channel 2 is important for retinal function and protects against light-induced retinal degeneration. J. Neurosci..

[477] Peng Y.W., Zallocchi M., Wang W.M., Delimont D., Cosgrove D. (2011). Moderate light-induced degeneration of rod photoreceptors with delayed transducin translocation in shaker1 mice. Investig. Ophthalmol. Vis. Sci..

[478] Tian M., Wang W., Delimont D., Cheung L., Zallocchi M., Cosgrove D., Peng Y.W. (2014). Photoreceptors in whirler mice show defective transducin translocation and are susceptible to short-term light/dark changes-induced degeneration. Exp. Eye Res..

[479] Li G., Anderson R.E., Tomita H., Adler R., Liu X., Zack D.J., Rajala R.V. (2007). Nonredundant role of Akt2 for neuroprotection of rod photoreceptor cells from light-induced cell death. J. Neurosci..

[480] Datta S., Cano M., Ebrahimi K., Wang L., Handa J.T. (2017). The impact of oxidative stress and inflammation on RPE degeneration in non-neovascular AMD. Prog. Retin. Eye Res..

[481] Khan J.C., Thurlby D.A., Shahid H., Clayton D.G., Yates J.R., Bradley M., Moore A.T., Bird A.C., Genetic Factors in AMD Study (2006). Smoking and age related macular degeneration: The number of pack years of cigarette smoking is a major determinant of risk for both geographic atrophy and choroidal neovascularisation. Br. J. Ophthalmol..

[482] Cano M., Thimmalappula R., Fujihara M., Nagai N., Sporn M., Wang A.L., Neufeld A.H., Biswal S., Handa J.T. (2010). Cigarette smoking, oxidative stress, the anti-oxidant response through Nrf2 signaling, and Age-related Macular Degeneration. Vis. Res..

[483] Fujihara M., Nagai N., Sussan T.E., Biswal S., Handa J.T. (2008). Chronic cigarette smoke causes oxidative damage and apoptosis to retinal pigmented epithelial cells in mice. PLoS ONE.

[484] Ebrahimi K.B., Cano M., Rhee J., Datta S., Wang L., Handa J.T. (2018). Oxidative Stress Induces an Interactive Decline in Wnt and Nrf2 Signaling in Degenerating Retinal Pigment Epithelium. Antioxid. Redox Signal..

[485] Farese R.V., Veniant M.M., Cham C.M., Flynn L.M., Pierotti V., Loring J.F., Traber M., Ruland S., Stokowski R.S., Huszar D. (1996). Phenotypic analysis of mice expressing exclusively apolipoprotein B48 or apolipoprotein B100. Proc. Natl. Acad. Sci. USA.

[486] Schmidt-Erfurth U., Rudolf M., Funk M., Hofmann-Rummelt C., Franz-Haas N.S., Aherrahrou Z., Schlotzer-Schrehardt U. (2008). Ultrastructural changes in a murine model of graded Bruch membrane lipoidal degeneration and corresponding VEGF164 detection. Investig. Ophthalmol. Vis. Sci..

[487] Malek G., Johnson L.V., Mace B.E., Saloupis P., Schmechel D.E., Rickman D.W., Toth C.A., Sullivan P.M., Bowes Rickman C. (2005). Apolipoprotein E allele-dependent pathogenesis: A model for age-related retinal degeneration. Proc. Natl. Acad. Sci. USA.

[488] Perusek L., Maeda T. (2013). Vitamin A derivatives as treatment options for retinal degenerative diseases. Nutrients.

[489] Perusek L., Maeda A., Maeda T. (2015). Supplementation with vitamin a derivatives to rescue vision in animal models of degenerative retinal diseases. Methods Mol. Biol..

[490] Guadagni V., Novelli E., Piano I., Gargini C., Strettoi E. (2015). Pharmacological approaches to retinitis pigmentosa: A laboratory perspective. Prog. Retin. Eye Res..

[491] Cai X., McGinnis J.F. (2012). Oxidative stress: The achilles’ heel of neurodegenerative diseases of the retina. Front. Biosci. Landmark. Ed..

[492] Komeima K., Rogers B.S., Campochiaro P.A. (2007). Antioxidants slow photoreceptor cell death in mouse models of retinitis pigmentosa. J. Cell Physiol..

[493] Ebert S., Weigelt K., Walczak Y., Drobnik W., Mauerer R., Hume D.A., Weber B.H., Langmann T. (2009). Docosahexaenoic acid attenuates microglial activation and delays early retinal degeneration. J. Neurochem..

[494] Yu M., Yan W., Beight C. (2018). Lutein and Zeaxanthin Isomers Reduce Photoreceptor Degeneration in the Pde6b (rd10) Mouse Model of Retinitis Pigmentosa. Biomed. Res. Int..

[495] Komeima K., Rogers B.S., Lu L., Campochiaro P.A. (2006). Antioxidants reduce cone cell death in a model of retinitis pigmentosa. Proc. Natl. Acad. Sci. USA.

[496] Barone I., Novelli E., Piano I., Gargini C., Strettoi E. (2012). Environmental enrichment extends photoreceptor survival and visual function in a mouse model of retinitis pigmentosa. PLoS ONE.

[497] Sundin O.H., Leppert G.S., Silva E.D., Yang J.M., Dharmaraj S., Maumenee I.H., Santos L.C., Parsa C.F., Traboulsi E.I., Broman K.W. (2005). Extreme hyperopia is the result of null mutations in MFRP, which encodes a Frizzled-related protein. Proc. Natl. Acad. Sci. USA.

[498] Sundin O.H. (2005). The mouse’s eye and Mfrp: Not quite human. Ophthalmic Genet..

[499] Kameya S., Hawes N.L., Chang B., Heckenlively J.R., Naggert J.K., Nishina P.M. (2002). Mfrp, a gene encoding a frizzled related protein, is mutated in the mouse retinal degeneration 6. Hum. Mol. Genet..

[500] Almoallem B., Arno G., De Zaeytijd J., Verdin H., Balikova I., Casteels I., de Ravel T., Hull S., Suzani M., Destree A. (2020). The majority of autosomal recessive nanophthalmos and posterior microphthalmia can be attributed to biallelic sequence and structural variants in MFRP and PRSS56. Sci. Rep..

[501] Chekuri A., Sahu B., Chavali V.R.M., Voronchikhina M., Soto-Hermida A., Suk J.J., Alapati A.N., Bartsch D.U., Ayala-Ramirez R., Zenteno J.C. (2019). Long-Term Effects of Gene Therapy in a Novel Mouse Model of Human MFRP-Associated Retinopathy. Hum. Gene Ther..

[502] Gibson F., Walsh J., Mburu P., Varela A., Brown K.A., Antonio M., Beisel K.W., Steel K.P., Brown S.D. (1995). A type VII myosin encoded by the mouse deafness gene shaker-1. Nature.

[503] Lentz J.J., Gordon W.C., Farris H.E., MacDonald G.H., Cunningham D.E., Robbins C.A., Tempel B.L., Bazan N.G., Rubel E.W., Oesterle E.C. (2010). Deafness and retinal degeneration in a novel USH1C knock-in mouse model. Dev. Neurobiol..

[504] Karan G., Lillo C., Yang Z., Cameron D.J., Locke K.G., Zhao Y., Thirumalaichary S., Li C., Birch D.G., Vollmer-Snarr H.R. (2005). Lipofuscin accumulation, abnormal electrophysiology, and photoreceptor degeneration in mutant ELOVL4 transgenic mice: A model for macular degeneration. Proc. Natl. Acad. Sci. USA.

[505] Grishchuk Y., Stember K.G., Matsunaga A., Olivares A.M., Cruz N.M., King V.E., Humphrey D.M., Wang S.L., Muzikansky A., Betensky R.A. (2016). Retinal Dystrophy and Optic Nerve Pathology in the Mouse Model of Mucolipidosis IV. Am. J. Pathol..

[506] Amir N., Zlotogora J., Bach G. (1987). Mucolipidosis type IV: Clinical spectrum and natural history. Pediatrics.

[507] Park H., Qazi Y., Tan C., Jabbar S.B., Cao Y., Schmid G., Pardue M.T. (2012). Assessment of axial length measurements in mouse eyes. Optom. Vis. Sci..

[508] Ruppersburg C.C., Hartzell H.C. (2014). The Ca2+-activated Cl- channel ANO1/TMEM16A regulates primary ciliogenesis. Mol. Biol. Cell.

[509] He M., Ye W., Wang W.J., Sison E.S., Jan Y.N., Jan L.Y. (2017). Cytoplasmic Cl(-) couples membrane remodeling to epithelial morphogenesis. Proc. Natl. Acad. Sci. USA.

[510] Gupta A., Fabian L., Brill J.A. (2018). Phosphatidylinositol 4,5-bisphosphate regulates cilium transition zone maturation in Drosophila melanogaster. J. Cell Sci..

[511] Bhatia V., Valdes-Sanchez L., Rodriguez-Martinez D., Bhattacharya S.S. (2018). Formation of 53BP1 foci and ATM activation under oxidative stress is facilitated by RNA:DNA hybrids and loss of ATM-53BP1 expression promotes photoreceptor cell survival in mice. F1000Res.

[512] Muller B., Ellinwood N.M., Lorenz B., Stieger K. (2018). Detection of DNA Double Strand Breaks by gammaH2AX Does Not Result in 53bp1 Recruitment in Mouse Retinal Tissues. Front. Neurosci..

