# Supplementary material for: Mouse Models of Inherited Retinal Degeneration with Photoreceptor Cell Loss
Source: Cells. 2020 Apr 10;9(4):931. doi: 10.3390/cells9040931 (PMC7227028; doi:10.3390/cells9040931)
Supplement: Supplementary file 1 [file cells-09-00931-s001.zip › Supplementary Files/Collin_et_al_Supplementary Captions.docx]

Table S1: Monogenic mouse RD models with PR cell loss. Each row represents an independent study of a mouse model. Tabular columns indicate biological processes assigned to each gene as described in the text (section, category), gene and allele names, the PMID of the study reporting PR cell loss, strain background, mutation type, genetic alteration, and ocular disease(s) associated with variants of the corresponding gene in humans. Subsequent columns provide information about the progression of PR cell loss, as measured by percentage degeneration of PR cells relative to controls at various ages as reported in the associated publications or estimated from the data presented, ERG observations, parameters from exponential fitting of the data, zygosity (homozygous, hom; heterozygous, het; X-linked males are hemizyous, hemi), and additional pertinent information, such as time points and the age at peak of apoptosis assays, and whether *rd1* or *rd8* was removed prior to characterization. Hyperlinks to MGI are included for genes and allele names, and to PubMed, for PMIDs.

Table S2: Description of gene/protein symbols used in the text and figures. Column 1 lists mouse gene symbols, which by convention are italicized with the first letter capitalized. Human genes symbols are italicized and capitalized in full. Corresponding protein symbols in both mouse and human are capitalized in full without italics. Column 2 indicates the MGI identification number for the gene. Two human genes on the list, *ARMS2* and *EYS*, are not present in mice. Column 3 provides a description of the gene product.

Figure S1: OCT and fundus images of C57BL/6J control mice at various ages. The images serve as controls to those in Figure 4. *Yellow bars* indicate full retinal thickness. Values correspond to mouse age at time of imaging (weeks).
