# Supplementary figures and images for: Mouse Models of Inherited Retinal Degeneration with Photoreceptor Cell Loss
Source: Cells. 2020 Apr 10;9(4):931. doi: 10.3390/cells9040931 (PMC7227028; doi:10.3390/cells9040931)

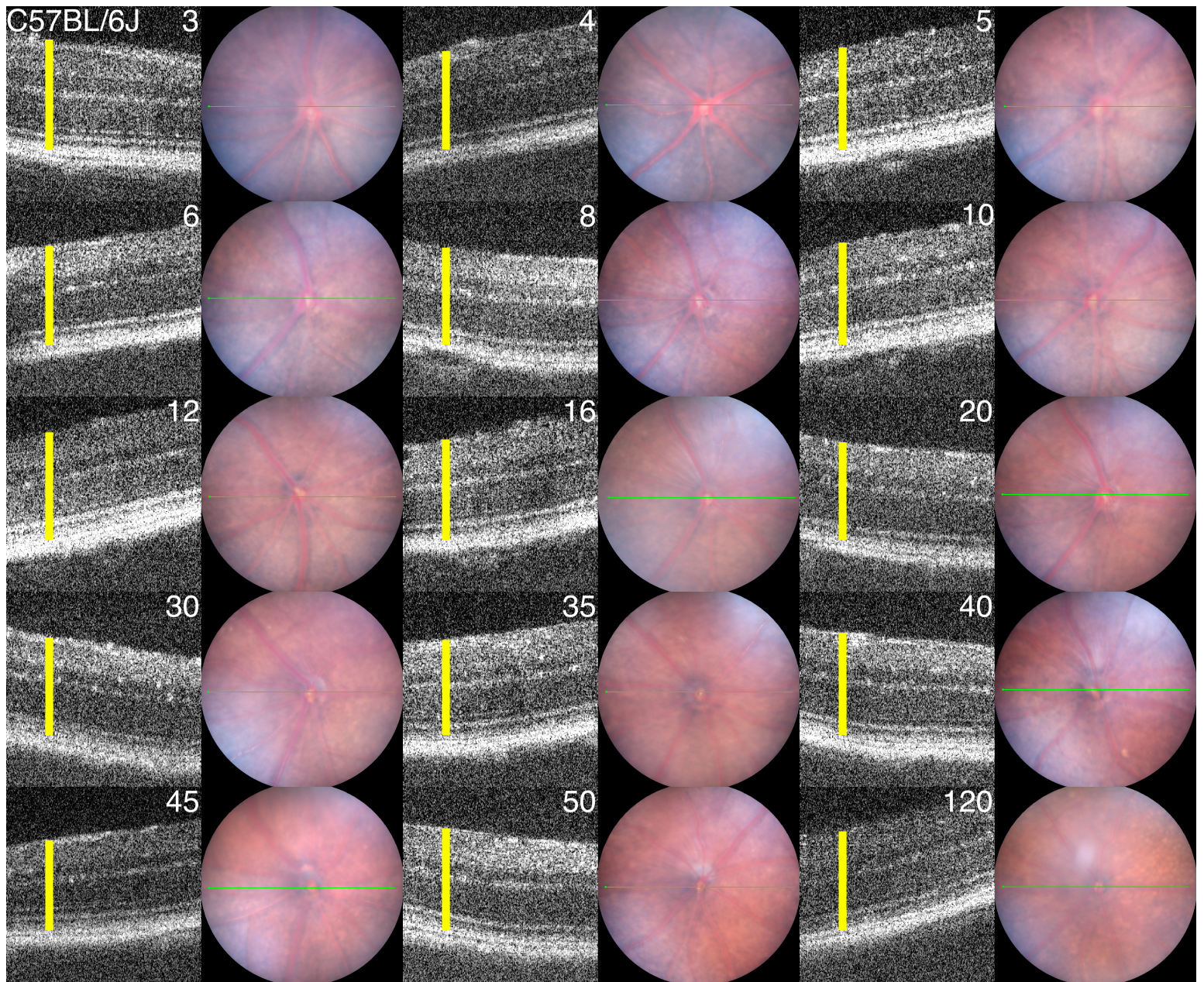

Supplement: Supplementary file 1 [file cells-09-00931-s001.zip › Supplementary Files/Collin_et_al_Figure S1.pdf]
